# Benchmarking Hierarchical Double-Ended Transition-State Search Methods for Metal-Catalyzed Reactions

**DOI:** 10.1021/acs.jctc.6c00992

**Published:** 2026-09-25

**Authors:** Shoubhik R. Maiti, David Buttar, Fernanda Duarte

**Affiliations:** † Department of Chemistry, 6396 University of Oxford, Mansfield Road, Oxford OX1 3TA, U.K.; ‡ Data Science and Modelling, 4978 Pharmaceutical Sciences, R&D, AstraZeneca, Macclesfield SK10 2NA, U.K.

## Abstract

Efficient and reliable identification of transition states (TS) is critical for reaction modeling. Among the available approaches, the combination of double-ended TS search with eigenvector-following, referred to as hierarchical TS search, is an effective tool for locating TSs starting from reactant and product geometries. However, systematic benchmarking of these methods on transition metal-catalyzed reactions remains limited. Here, we benchmark eight pipelined hierarchical methods using out-of-the-box settings on a diverse set of 30 single-step, closed-shell metal-catalyzed reactions, evaluating their success rate and computational efficiency. Most workflows achieve high success rates across the data set, indicating that modern double-ended methods are generally robust for locating TSs in elementary organometallic reactions, and the main differences between methods lie in their computational efficiency. The benchmark also reveals several key trends: (i) the final eigenvector-following step requires an accurate Hessian matrix or explicitly calculated *min-mode* for a high success rate, (ii) TS searches from interpolation-only paths (e.g., image-dependent pair potential, IDPP) and bracket methods (e.g., Dewar-Healy-Stewart, DHS) are often more efficient than popular methods, such as climbing image nudged elastic band (CI-NEB), (iii) different hierarchical workflows can converge to different TSs even when starting from the same two minima, (iv) larger structural changes between reactants and products are associated with lower success rates and higher computational costs. We also show that further parameter tuning can reduce computational expense without reducing the success rate on our data set. These conclusions are further supported by two case studies involving industrially relevant organometallic reactions.

## Graphical abstract

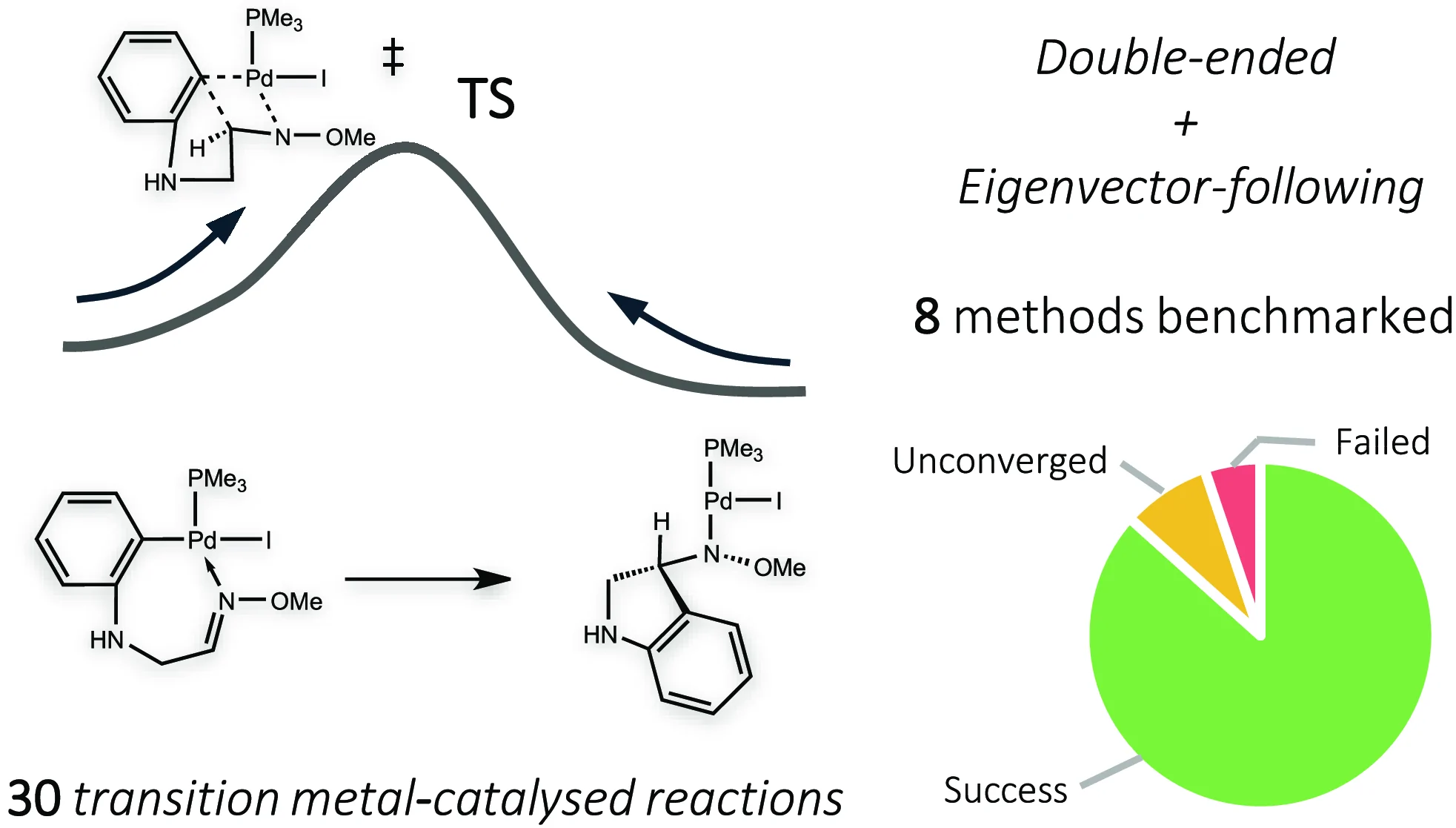

## 1 Introduction

A crucial step in elucidating reaction mechanisms and assessing their kinetic feasibility is the identification and characterization of stationary points on the potential energy surface (PES). The minimum energy path (MEP) connecting two minima, reactants and products, through the lowest energy transition state (TS) represents a natural choice for a reaction coordinate. Mathematically, a TS corresponds to a first-order saddle point (SP) on the PES, where the Hessian, i.e., the second derivative matrix of energy with respect to the nuclear positions, has exactly one negative eigenvalue, and the associated eigenvector indicates the TS mode.^1^ Within transition state theory (TST),^2^ the characterization of TSs and calculation of their activation barriers provide access to macroscopic reaction rates, which allows the identification of rate-limiting steps and comparison with experimental data.

While locating energy minima on a PES is relatively straightforward, locating TSs remains a challenging and time-consuming process, often requiring expert intuition or prior knowledge.^3^ To succeed, TS searches must efficiently direct the optimization toward a maximum along the correct coordinate while minimizing the energy along all orthogonal directions.^4^ The development of efficient TS search algorithms is an active area of research; current methods can be broadly classified into two categories: (i) single-ended and (ii) double-ended methods (Figure 1). Single-ended methods begin with a single geometry, while double-ended methods require both the reactant and product geometries as input.^4^

**Figure 1:**
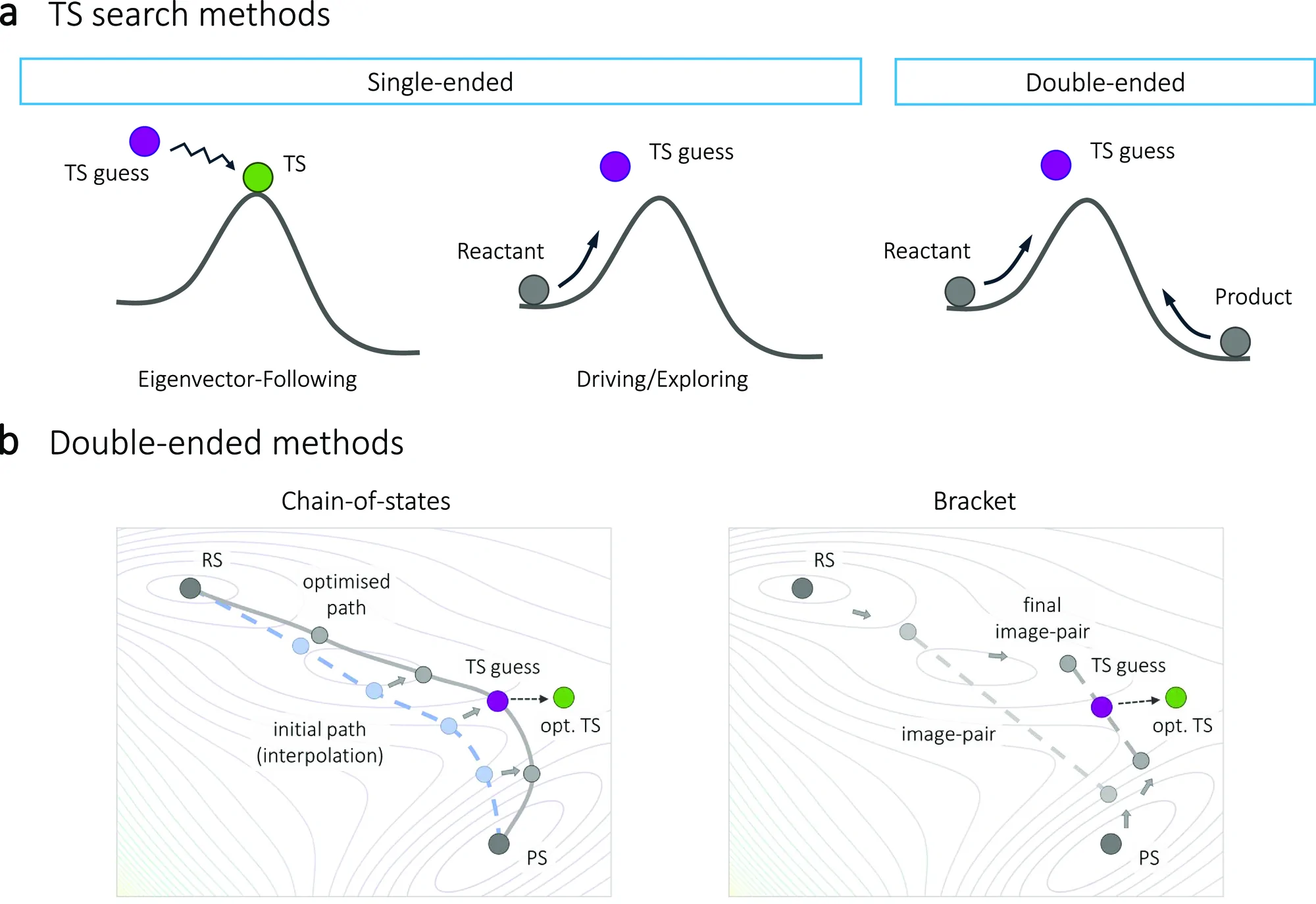
TS search methods. (a) Single-ended methods, classified as eigenvector-following or coordinate-driving, are illustrated on a 1D energy plot alongside double-ended methods. (b) Types of double-ended methods: chain-of-states and bracket methods, shown on a 2D Müller-Brown surface.^5^ The dotted line represents interpolation between reactant state (RS) and product state (PS), and the purple circle marks the TS guess obtained from the double-ended method. This guess is refined using an eigenvector-following approach to obtain the final TS (green circle).

In eigenvector-following single-ended methods, the energy is maximized along the Hessian mode with the lowest eigenvalue (*min-mode*), corresponding to the TS mode, and minimized along all orthogonal directions until the gradient falls below a convergence threshold.^4^ Although these methods can, in principle, start from any point in the PES, their efficiency and success strongly depend on the quality of the initial guess geometry—which must be near the TS structure.

A widely used class of eigenvector-following approaches is the quasi-Newton family, among which the Partitioned Rational Function Optimization (P-RFO) by Baker et al. is especially popular. It is implemented in QM software packages such as ORCA^6^ and Q-Chem.^7^ While P-RFO by construction has excellent convergence properties, its reliance on accurate Hessian information means that its computational cost scales poorly with system size.^4,8,9^ To address this limitation, Hessian-free alternatives have been developed. These include the hybrid eigenvector following (HEVF)^10,11^ and the independent implementation in the dimer method.^9,12^ Both methods use finite differentiation of gradients to estimate the *min-mode*. However, like quasi-Newton methods, they are unlikely to converge to the correct TS if the starting guess is poor.

Other single-ended methods can be broadly classified as either coordinate-driving or PES-exploration methods (Figure 1a), depending on whether the search is biased. Coordinate-driving methods require a single chemical structure^4^ and a set of coordinates (or atoms) that guide the system along a predefined reaction path. Examples include the commonly used relaxed scan method,^4^ single-ended growing string method (SE-GSM),^13^ reduced PES (RPES) methods,^14,15^ and artificial force induced reaction (AFIR).^16^ On the other hand, PES-exploration methods explore possible TSs close to the starting point in an unbiased manner; examples include the anharmonic downward distortion following (ADDF) method,^17^ and the ab initio nanoreactor (AINR) approach.^18^

Double-ended methods require the geometries of the two end-point minima connected by a continuous path that contains at least one TS. This choice constrains the exploration of the PES to the region between these two points, reducing the computational cost. With sufficient iterations and tight convergence criteria, double-ended methods can closely approach the true TS. However, the highest point obtained is not strictly a TS but rather a TS guess. This is because double-ended methods do not directly minimize the gradient, and the path tangent at the highest point is only an approximation to the true TS mode eigenvector.^19^ Therefore, further refinement with an eigenvector-following optimization, which minimizes the gradient, is typically required to reach the saddle point.^20^ Given the computational cost of tight convergence in double-ended methods, they are generally only converged enough to yield a reasonable TS guess. Combinations of double-ended methods with eigenvector-following, also referred to as “hierarchical TS search”,^20^ have been used to efficiently and reliably obtain TSs.^19,21,22^

In cases where the two starting minima are connected by multiple TSs and intermediate(s), the repeated application of hierarchical double-ended methods can be used to find each TS in the path, following the TS eigenvector downward on both sides to find and optimize the connected minima. Based on a distance metric,^22,23^ pairs of unconnected minima can be connected iteratively with the above procedure until a fully connected path is obtained. Examples of such workflows include the missing connection algorithm in OPTIM and PATHSAMPLE packages from Wales and co-workers,^22^ and AutoTS proposed by Friesner and others.^24^

Double-ended methods can be classified into two broad categories — (i) chain-of-states and (ii) bracket methods (Figure 1). The chain-of-states methods approximate the MEP between the initial and final geometries using a discrete path of intermediate configurations called “images”. Examples include the nudged elastic band (NEB)^25,26^ and its variants, climbing image NEB (CI-NEB),^27^ doubly-NEB (DNEB),^28^ string methods, including the finite-temperature string (FTS)^29^ and zero-temperature string (ZTS)^30,31^ variants, as well as the double-ended growing string method (DE-GSM).^21,32^ The chain-of-states methods start by generating a guess path of images using an interpolation scheme, e.g., linear interpolation in Cartesian or internal coordinates,^33^ finding the geodesic path,^34^ linear synchronous transit (LST),^35^ image-dependent pair-potential (IDPP),^36^ quasi-continuous interpolation (QCI)^37^ or flat-bottomed elastic network model (FB-ENM)^38,39^ schemes. The set of images is then relaxed toward the true MEP using gradient information.

In NEB-based methods, the images are connected by harmonic springs, preventing them from sliding down to local minima and ensuring they are evenly distributed along the path. This is achieved by only minimizing the component of atomic forces perpendicular to the path and the spring forces parallel to the path.^25^ In CI-NEB, after a certain residual force or iteration threshold is reached, the highest energy image is forced to “climb” upward in the tangent direction along the path (and relaxed along orthogonal directions) to come closer to a saddle point, i.e., TS.^27^ The path tangent at the climbing image is an approximation of the TS mode, and its accuracy is important in determining the success of the method.^40^

DNEB is a modification of the original NEB method, where a component of the spring forces perpendicular to the path, which does not interfere with the perpendicular component of atomic forces, is added back into the total force.^28^ This helps to straighten the band and improve efficiency.^41^ DNEB cannot be tightly converged to the MEP since the perpendicular spring force term increases as the path comes close to the MEP and nudges it away from convergence,^41^ but it is generally employed to suggest TS guess candidates for eigenvector-following refinement.

If the initial interpolated path is poor, then the convergence of NEB-based methods can be slow. To circumvent this issue, a different approach to generating images is used by DE-GSM;^21^ here, the path is “grown” from the end points by iteratively adding new images using an interpolation scheme (such as LST^21^). As the string is built up, it is simultaneously minimized by using the atomic forces parallel to the path, thus avoiding high-energy regions of the PES. To ensure the images are spaced evenly, they are repositioned every few steps. Chakraborty and co-workers showed that DE-GSM is faster at obtaining accurate TS guesses than NEB or ZTS.^21^ However, repositioning the images may lead to convergence issues.^42^ Later developments from Head-Gordon^43^ and Zimmerman^32^ introduced the use of internal coordinates for the minimization and interpolation as well as a “climbing image” method in DE-GSM.

One drawback of chain-of-states methods is the need to optimize multiple interacting images in each iteration, which can be computationally expensive when many images are required to accurately describe the MEP. A possible solution involves increasing the number of images in high-energy regions and reducing it in lower-energy regions, thereby improving the resolution of the PES near the TS. In NEB-like methods, this can be achieved by using energy-weighted springs, which make springs stiffer in higher-energy regions.^40^ Similarly, in string methods, energy-weighted parametrization ensures a higher density of points describing the path in higher energy regions.^31^ These strategies enable chain-of-states methods to operate with fewer images than otherwise necessary to accurately describe the path. The recently proposed double-ended direct MaxFlux (DMF) method iteratively updates energy evaluation points on the path so that they concentrate near the higher-energy regions. When tested in organic reactions, this method has been shown to be more efficient compared to traditional NEB methods.^44^

An alternative approach is the so-called “bracket” method, which uses only two images (an “image-pair”) at any given step and attempts to bracket the TS from both end points without approximating the MEP. Examples of this approach include the Dewar-Healy-Stewart (DHS) method,^45^ binary-image transition-state search (BITSS),^46^ elastic image pair (EIP)^47^ and its improved variant (i-EIP),^48^ and convergence of moving pairs against surface saddles (COMPASS).^49^

DHS searches for a TS between two images, the reactant and product states.^45^ Each DHS step consists of moving the lower energy image toward the higher energy image along the linear vector connecting the two. Then, the energy of the moved image is minimized while keeping the distance to the other image constant to prevent it from going downhill. The images climb upward toward each other until the distance between the image-pair is low enough to signal convergence. The higher energy of the image-pair can be taken as the TS guess.

The EIP method also optimizes two images, one on the reactant and another on the product side, simultaneously to approach the TS.^47^ The initial image-pair is generated on both sides of the estimated peak along an interpolated path between the reactant and product. The optimization of the image-pair is carried out using atomic forces perpendicular to the image-pair vector to prevent the two images from falling downhill. Adaptive harmonic spring forces are added between the two images to reduce the distance and keep the two images with similar energy.

While EIP has been shown to be more efficient and have fewer convergence issues than NEB,^47^ it performs poorly on flat PES and large systems. To address this problem, an improved version called i-EIP has been proposed by Lei and others.^48^ The i-EIP method avoids harmonic springs and instead uses microiterations to reduce the distance between the image pairs, minimizing the perpendicular component of the gradient while keeping the energies similar. At convergence, the midpoint of the line connecting the two images is used as the TS guess.^48^

Despite the availability of several double-ended methods, their comparative performances in transition metal (TM) systems remain largely unexplored. Previous benchmarks have focused on organic systems or surface processes.^40,50^ This situation is problematic since TM catalysts are important for many industrial processes. The ability to model the TSs will help in understanding the reactivity of TM catalysis and designing novel catalysts.^51,52^ While the performance of density functional theory (DFT) has been evaluated in predicting the activation barriers for TM-catalyzed reactions,^53^ to the best of our knowledge, no comparative benchmarks exist on TS search methods in these systems. Two main factors hinder systematic benchmarking. First, while there are many comprehensive transition-state data sets available for main-group reactions,^19,54,55^ this data is scarce in TM-catalyzed reactions.^53^ Second, metals in general show more flexible and varied coordination geometries compared to organic elements, leading to a complicated PES, which makes interpreting TS data challenging.

To address these challenges, we assess the out-of-the-box performance of eight hierarchical double-ended TS search methods from four software packages (all freely available for academics) on a set of 30 closed-shell TM-catalyzed reactions covering different TM centers and chemistries. It is important to note that each method has a set of settings and convergence criteria that affect its performance. For the end-user, tuning these settings to achieve optimal results is usually too time-consuming to be practical. Therefore, we have tested all methods with their default or recommended settings. Note that in this paper, we only consider molecular reactions, i.e., without periodic boundary conditions, and do not include reactions on surfaces.

## 2 Computational Methods

### 2.1 Double-ended TS Search Methods

The following hierarchical double-ended methods were tested using the default/recommended settings unless otherwise mentioned. We focused on widely used double-ended methods such as CI-NEB, DE-GSM, DHS and DNEB, all of which have also been previously benchmarked on a variety of systems.^19,40,41,50,56^ In addition, we test i-EIP, which has been successfully applied to transition metal reactions.^48^ In all cases, we consider the combination of double-ended method and eigenvector-following refinement together.

- **NEB-TS** ^40^ - This method refers to the energy-weighted CI-NEB (EW-CI-NEB) as implemented in ORCA software package v5.0.4. The initial path is generated with IDPP interpolation, and then EW-CI-NEB is used to converge the path and generate a TS guess. This guess is subsequently refined with a P-RFO optimization. The optimization starts from an updated Hessian based on the path tangent curvature. The ORCA implementation has been assessed on an extensive organic reaction set,^40^ and it has been used for successful TS searches in TM-catalyzed reactions.^57–59^ We also consider two modifications - **NEB-TS­(H)** where a calculated Hessian is used to start the P-RFO, and **IDPP-TS­(H)** where the NEB iterations are turned off (i.e., the highest energy point from the interpolated path is used as a guess).
- **GSM-EV-ES** ^32^ - The GSM-EV-ES method uses a climbing image DE-GSM calculation in internal coordinates, with an integrated quasi-Newton eigenvector-following TS refinement which uses an updated starting Hessian. It is implemented in the open-source C++ package molecularGSM, which in our case used ORCA to compute energies and gradients. This method has been extensively used in automated TS search workflows,^60–62^ as well as in modeling TM-catalyzed reactions.^63^
- **DHS-TS­(H)** ^45^ - The bracket method DHS has been implemented in our open-source Python package autodE.^3^ It also uses ORCA to obtain energies and gradients. The TS guess is refined with P-RFO optimization in ORCA starting from a calculated Hessian. This combination is referred to as DHS-TS­(H) hereafter. We chose this method because it is one of the oldest double-ended methods and it has shown good performance in a previous benchmark of surface reactions.^56^
- **i-EIP-TS­(H)** ^48^ - The i-EIP method was also implemented in autodE. The TS guess from i-EIP is refined with P-RFO starting from a calculated Hessian. This combination is referred to as i-EIP-TS­(H) hereafter. In its original publication, the method was found to be efficient in identifying TSs for five TM-catalyzed reactions.^48^
- **DNEB-HEV** ^28,37^ - The method starts from a QCI interpolation, and relaxes the path with DNEB. The TS guess from this path is refined with HEVF TS optimization. This has been referred to as the “revised connection algorithm” by Wales and co-workers; however, here we refer to it as DNEB-HEV for simplicity. The method is implemented in the open-source Fortran package OPTIM, which can employ ORCA (or other electronic structure package) for energies and gradients via an interface. The combination of DNEB and HEVF has been found to be the top performer among the double-ended methods in a benchmark of surface and condensed matter systems.^50^ We also consider the method **QCI-HEV**, which uses only the QCI interpolated path to obtain the TS guess.

The two methods IDPP-TS­(H) and QCI-HEV are hereafter referred to as interpolation-only methods since they obtain the TS guess directly from the interpolated path. For these methods, as well as the chain-of-states calculations (NEB-TS, NEB-TS­(H), GSM-EV-ES, DNEB-HEV), the number of images was set to eight. Tight convergence criteria were used for the final TS refinement across all methods to facilitate the comparison of TS geometries. See Section SI-1 for further details on the settings and convergence criteria used for each method. We reiterate that molecularGSM, autodE and OPTIM packages are wrapper codes that use DFT energies and gradients supplied by ORCA.

### 2.2 Electronic Structure Calculations

Quantum chemical calculations were run using the ORCA suite of programs^6,64,65^ (version 5.0.4). All benchmark calculations were performed at the PBE0-D3BJ/def2-SVP level of theory,^66,67^ using the resolution of the identity approximation (RIJCOSX),^68^ with the def2/J auxiliary basis set^69^ to speed up the calculations. Here, PBE0-D3BJ indicates the use of the PBE0 functional with the D3­(BJ) empirical dispersion correction.^70,71^ “Tight” wave function convergence criteria (tightSCF i.e., 1 × 10^–8^ *E*_h_ tolerance) and a larger grid (defgrid3) were also used in all calculations. Geometry optimizations in ORCA were performed using “tight” convergence criteria. Further details on settings used for each double-ended method and the final TS optimization can be found in Section SI-1.

To compare timings consistently, all benchmark calculations were run using eight cores on an Intel Xeon Platinum 8268 CPU (2.9 GHz). Other calculations were run on a variety of CPU architectures.

### 2.3 Data Set

A set of 30 single-step, closed-shell TM-catalyzed organometallic reactions (Table 1) was compiled to assess the performance of different double-ended methods. Of these, 23 were extracted from the MOBH35 data set,^53^ while the remaining seven were obtained directly from publications where the DFT-computed TS geometries were available. To reduce computational cost, six reactions were simplified by replacing bulky alkyl/aryl groups not involved in the reaction with smaller groups (details in Section SI-3.2). We have confirmed that the simplifications do not affect the reaction, i.e., the TS leads to the expected reactant and product. In addition, reaction-24 was modified to be consistent with our level of theory. See Section SI-3.2 for further details.

**Table 1:**
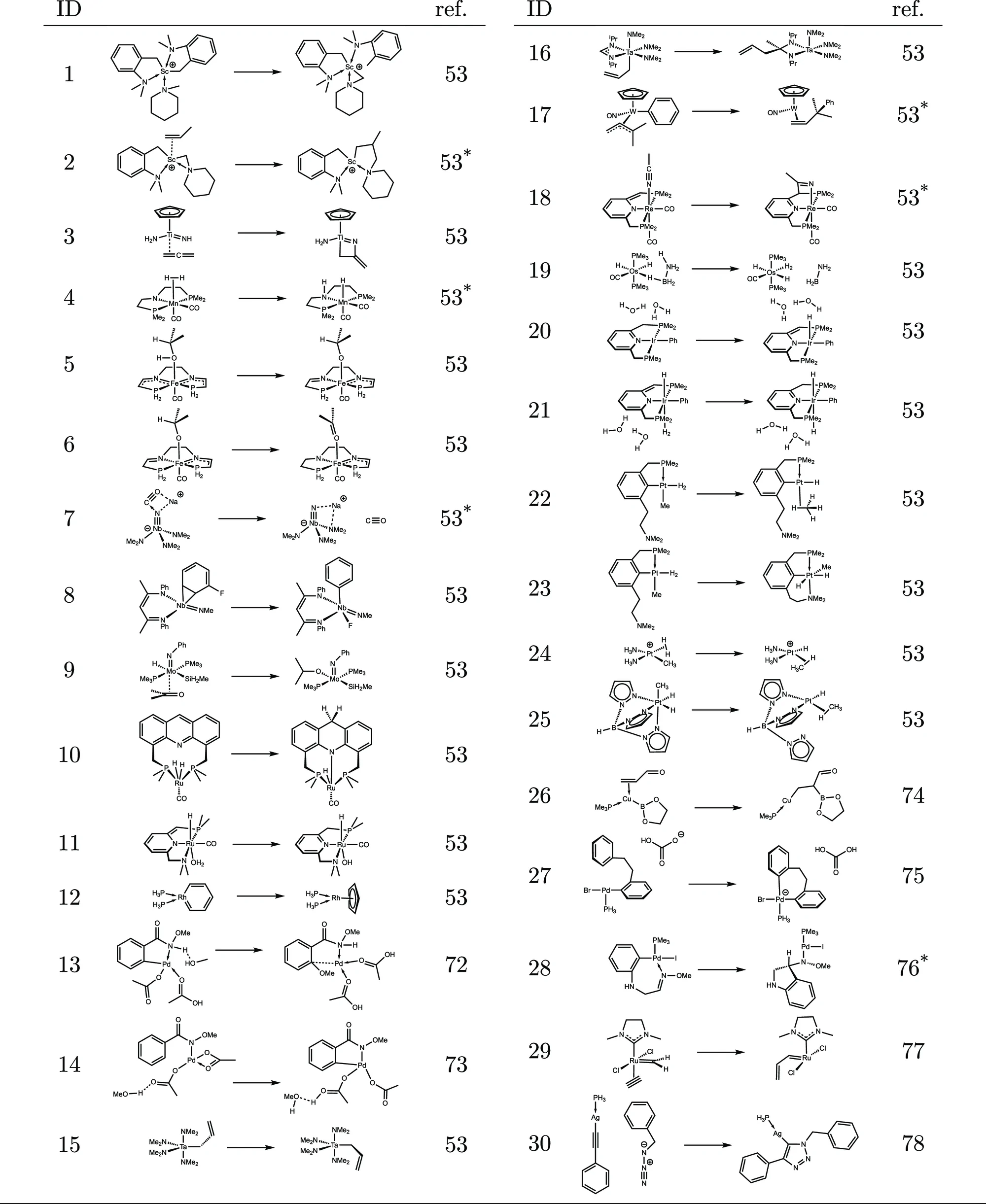
Set of 30 TM-catalyzed Reactions Extracted from Ref. 53, 72–78 Used to Assess the Performance of Double-ended Methods^a^

a To reduce computational cost, six reactions have been modified with substitutions of alkyl groups. The modified reactions are marked with *, with further details in Section SI-3.2.

The data set was prepared as follows:

1. The Cartesian coordinates (*xyz*) of the TS geometries were extracted from the original publication(s) and reoptimized at the PBE0-D3BJ/def2-SVP level of theory.
2. The imaginary mode in the TS is followed downhill on both sides to generate the IRC path.
3. The end-points from the IRC were minimized with tight convergence criteria to provide the geometries for the RS and PS.
4. The geometries of the optimized RS and PS were visualized and checked to ensure that they correspond to the correct chemical species.
5. These RS and PS geometries were then used as starting points for double-ended methods. Double-ended TS searches initiated from these minima are expected to reach the original TSs.

It should be noted that in our study, the words ”RS” and ”PS” are used interchangeably to represent two distinct minima connected by a TS. Further details on the data set preparation can be found in SI-3. A summary of the data set preparation procedure and the methods tested is presented in Figure 2.

**Figure 2:**
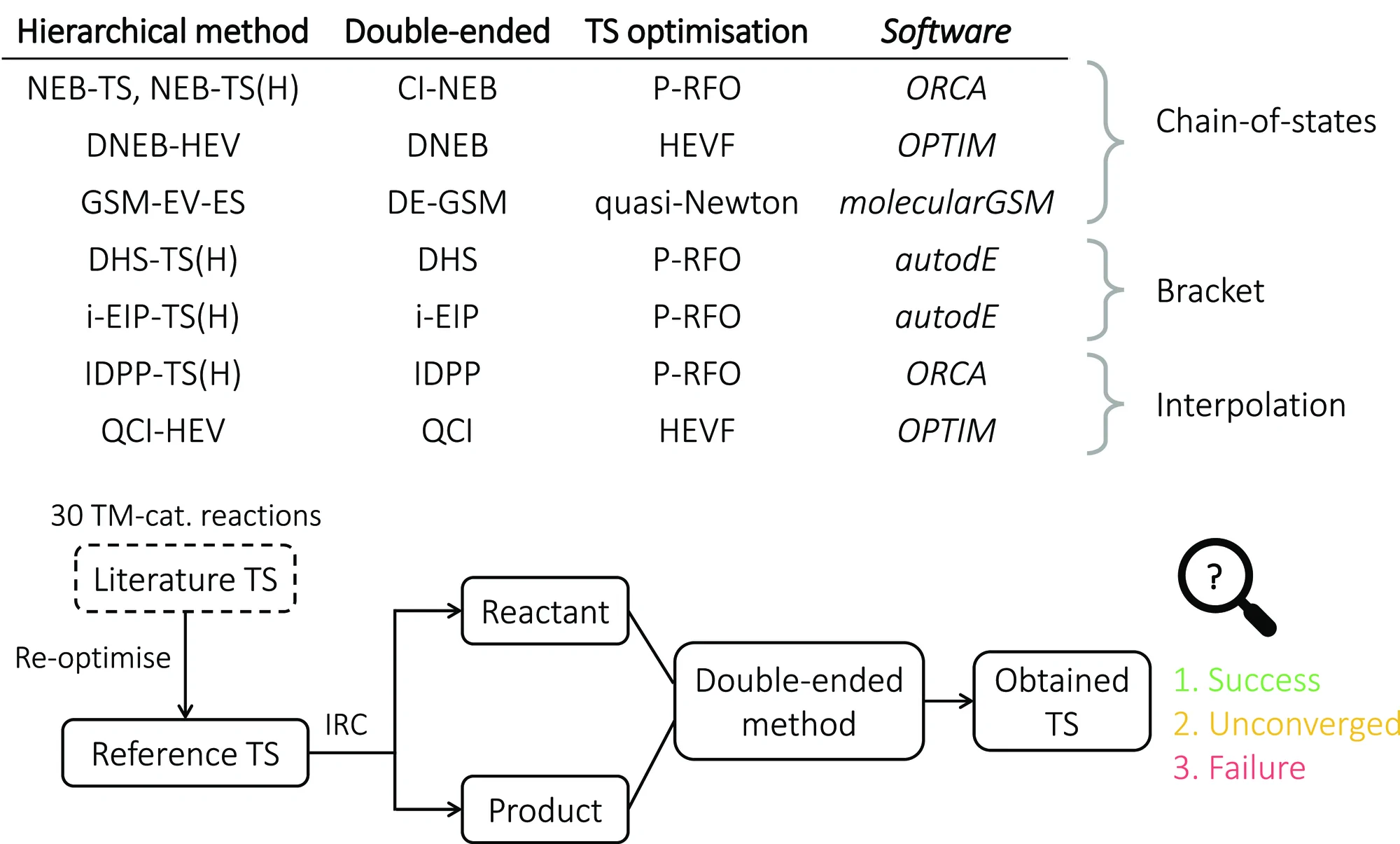
**(Top)** Summary of the hierarchical double-ended methods used in this study; please see Section-2.1 for details. **(Bottom)** Protocol for generation of data and testing.

### 2.4 Assessment Criteria for TS Search

We evaluated whether the hierarchical double-ended methods found the correct TS for the benchmark data set, and classified them into three categories—(i) successful, (ii) unconverged and (iii) failed. A TS search is considered successful if the obtained TS corresponds to the required chemical reaction *and* it satisfies our criteria for saddle points—(a) low gradient, with root-mean-squared gradient less than 5 × 10^–4^ *E*_h_ *a*_0_^–1^, and (b) only one imaginary frequency (ω_*i*_). However, TS searches often return geometries that do not meet these strict criteria, but are nevertheless close to the correct TS. These are classified as unconverged TSs since it is possible to recover the correct TS by running another cycle of optimization, or displacing along an unwanted secondary imaginary mode and then optimizing. The distinction between successful and unconverged TSs is necessary since additional intervention is required in the latter cases, after the hierarchical TS search is finished.

Note that the assessment was done on TSs obtained from the final eigenvector-following optimization run on the guess from double-ended methods. If the final TS optimization was halted due to reaching the maximum number of iterations, we took the last geometry from the optimization trajectory as the TS. The automated screening procedure for the assessment is described below.

We check if the TS from hierarchical double-ended methods matches the benchmark reference TS; the obtained TS is considered a match if it has (i) a root-mean squared deviation (RMSD) with the reference TS less than 0.15 Å and (ii) overlap greater than 0.9 between the *min-mode* eigenvectors of the obtained and reference TS. The RMSD is calculated over heavy atoms, but hydrogen atoms are included if they are involved in the reaction. All RMSD values reported hereafter are calculated using this protocol. The overlap is calculated as the dot product of normalized eigenvectors.

If a double-ended TS matches the benchmark reference and is a saddle point, it is assigned as successful. If it matches but is not a saddle point, it is assigned as an unconverged TS. In other cases, i.e., where the TS does not match the reference, manual analysis is required. For example, a conformer of the reference TS will have a high RMSD and cannot be assigned by the above procedure. Further details can be found in Section SI-4.2.

## 3 Results and Discussion

### 3.1 Benchmark Results

As described above, we classified the outcomes of the hierarchical double-ended methods into three categories: (i) successful, (ii) unconverged, and (iii) failed. Our benchmark set comprises 30 reactions and eight methods. However, we have removed reaction 8 from our analysis since the TSs could not be properly classified as either successful or failed. Further analysis and justification can be found below, in Section-3.2.1. All results presented below are based on 29 reactions with eight methods, i.e., 232 runs in total.

Of the 232 TS searches, (i) 201 were successful, (ii) 19 unconverged and (iii) only 12 were classified as failures. These results highlight that double-ended methods, in general, have a good success rate in TM-catalyzed reactions. Notably, these 12 failing cases arise from only eight reactions.

When examining the failure rates across methods (Figure 3a), we found that NEB-TS­(H) had the lowest failure rate (0/29), followed by DHS-TS­(H), i-EIP-TS­(H), QCI-HEV, GSM-EV-ES (all 1/29), and DNEB-HEV, IDPP-TS­(H) (both 2/29). In contrast, NEB-TS had the highest rate of failure (4/29). The lower performance of NEB-TS, when compared to NEB-TS­(H), is attributed to the use of an approximate Hessian in the TS optimization. This is in line with previous work by Jónsson and co-workers, on a main-group benchmark.^40^ GSM-EV-ES, which also uses an approximate Hessian for TS refinement, has a lower failure rate (1/29). However, in 12/30 cases, the TSs from GSM-EV-ES remained unconverged (i.e., high gradient or more than one imaginary mode), which indicates suboptimal performance of the TS optimizer in molecularGSM. As mentioned before, an accurate Hessian matrix is required to obtain the correct *min-mode* corresponding to the reaction coordinate, so that the TS optimizer can take uphill steps in the correct direction. QCI-HEV and DNEB-HEV, which calculate the *min-mode* by finite differentiation of gradient, also perform well, with only 1 and 2 failures out of 29 reactions, respectively. Overall, accurate information on the *min-mode*, either by finite difference or from an accurate Hessian, is important for successfully obtaining the refined TS from the double-ended TS guess.

**Figure 3:**
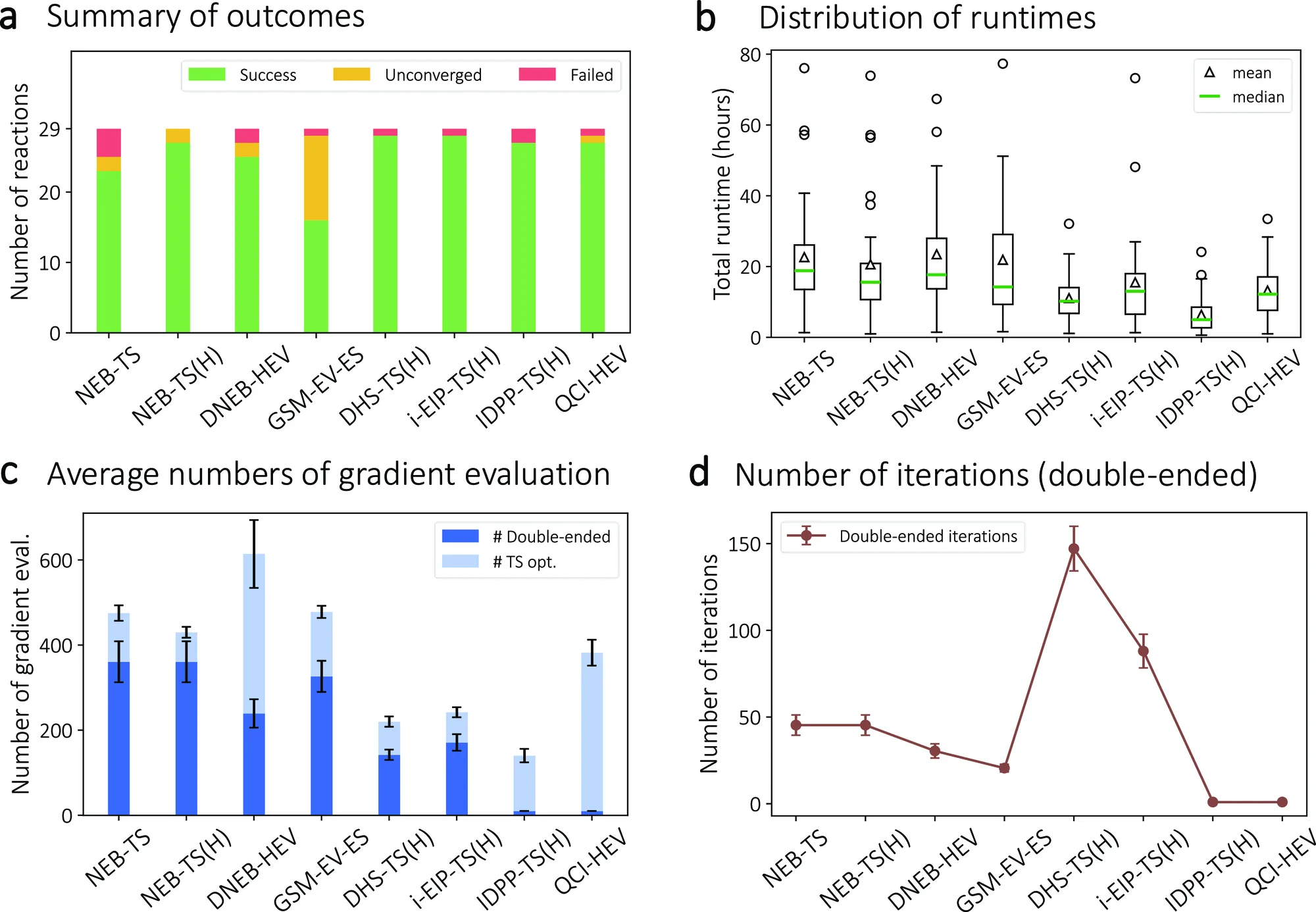
Benchmark results. (a) Number of successes (green), unconverged TSs (yellow) and failures (red) for each double-ended TS search method on the benchmark set. (b) Distribution of runtimes across methods, also showing the mean and median for each method. The boxplots show the range between the 75th and 25th percentiles of runtimes, and the whiskers show the farthest data points within 1.5 × interquartile range. (c) Average number of gradient evaluations during the double-ended (dark blue) and TS refinement (pale blue) step. (d) Average number of method iterations during the double-ended step. Error bars represent the standard error of the mean in both **c** and **d**.

#### 3.1.1 Efficiency

We compare the efficiency of the methods across 29 reactions in two ways: first, by comparing total run times on the same hardware (Figure 3b) and second, by considering the number of gradient evaluations during the double-ended and TS refinement steps (Figure 3c). For methods that calculate a Hessian matrix at the beginning of the TS optimization, we add a correction factor defined as the ratio between the wall-times required to calculate one Hessian and one gradient for that system i.e., the number of gradients equivalent to one Hessian in cost. Here, we have included all runs regardless of success, as excluding failed runs would introduce bias in the comparison; the success of a method is not known *a priori* to the end-user. Perfect comparisons of efficiency are impossible due to the different implementations for each method, especially the convergence criteria. For example, the P-RFO TS optimizer in ORCA uses gradient and step size thresholds in internal coordinates and energy change as convergence thresholds, while the HEVF optimizer in OPTIM checks only the gradient and step sizes in Cartesian coordinates. These thresholds cannot be matched since the transformation of Cartesian to internal coordinates is system-dependent. Similar differences also exist for the double-ended step e.g., CI-NEB in ORCA uses gradient component thresholds for all images, and a separate threshold for the climbing image, while GSM-EV-ES uses only the RMS gradient for the path. These disparities will invariably lead to differing numbers of iterations required to converge. The total run-times are also affected by the size of the chemical species, which determines the time required for each energy, gradient or Hessian calculation.

Nevertheless, it is still informative to compare the distributions across the same data set for different methods on the same hardware. Analysis of the run-times shows that chain-of-states methods, especially DNEB-HEV and NEB-TS, are the most costly, with median run-times >18 h. IDPP-TS­(H) and DHS-TS­(H) are the fastest and second fastest methods with median run-times of 4.9 and 10.2 h. The whiskers and the outliers in Figure 3d show the span of run-times. IDPP-TS­(H), DHS-TS­(H) and QCI-HEV have a low data spread, with no run-times above 48 h. This indicates that, for these methods, the computational cost is more stable across different reaction types and systems. The other methods have a larger spread of run-times and a number of outliers.

Next, we compared the average number of gradient evaluations for each method, separated into the double-ended step and the TS optimization step (Figure 3c). The chain-of-states methods are the most expensive, and most of the computational effort is spent in the double-ended part, i.e., optimizing the path. In the double-ended step, the four chain-of-states methods use between 239 and 361 gradients on average, for DNEB-HEV and NEB-TS, respectively; in comparison, the bracket methods are less expensive, using 142 and 171 gradients for DHS-TS­(H) and i-EIP-TS­(H), respectively. Interpolation-only methods IDPP-TS­(H) and QCI-HEV incur negligible costs in the double-ended step as they only calculate energies over the interpolated path.

In the final TS optimization step, the P-RFO algorithm, used in NEB-TS, NEB-TS­(H), DHS-TS­(H), i-EIP-TS­(H), uses around 69–130 gradient calls on average. Similarly, the quasi-Newton method used in GSM-EV-ES requires 151 gradients. In comparison, the HEVF optimizer in QCI-HEV and DNEB-HEV uses an average of 372 and 375 gradients, respectively. The higher cost of HEVF is due to the multiple gradient evaluations required for finite differentiation at each step, while P-RFO and quasi-Newton methods require just one gradient in each step. We also note that the TS optimization in NEB-TS­(H) is less expensive (69 gradients after correcting for Hessian) compared to NEB-TS (114 gradients). This indicates that the additional cost of an analytic Hessian is offset by the faster convergence of the TS optimization.^40^

Overall, considering both the double-ended step and the TS optimization, IDPP-TS­(H) is the least expensive method, with an average of 140 gradient calls in total, and DNEB-HEV is the most expensive with 614 gradients in total.

When discussing efficiency in terms of run-times, it is important to note that this quantity can depend strongly on the parallelization strategy used in a particular implementation. Chain-of-states methods have a natural advantage because the energy and gradient of individual images can, in principle, be computed in parallel. The current ORCA implementation of NEB-TS, NEB-TS­(H) and IDPP-TS­(H) exploit this advantage. In contrast, while GSM-EV-ES (molecularGSM), DNEB-HEV, and QCI-HEV (OPTIM) could also benefit from image-level parallelization, this is not currently implemented. Among the bracket methods, only i-EIP-TS­(H) in autodE parallelizes the optimization of the image pair, whereas DHS-TS­(H) proceeds sequentially.

Consequently, NEB-TS, NEB-TS­(H), IDPP-TS­(H) and i-EIP-TS­(H) would be expected to scale more favorably with increasing system size and number of CPUs. Of course, the calculation of the wave function itself (i.e., energy/gradient) can also benefit from parallelization, which is the case in ORCA. Assuming abundant computational resources and optimal image-level parallelization, the efficiency of a double-ended method is reflected by the number of iterations required for convergence. Here one iteration indicates each step in optimizing the entire path of images for chain-of-states methods, and each step where the image-pair is moved in bracket methods. Therefore, we also compare the average number of iterations in the double-ended step (Figure 3d). Under this idealized scenario, the chain-of-states methods require substantially fewer iterations (<50 iterations) compared to bracket methods (>85), suggesting superior scalability. DHS requires the highest iteration count (142), reflecting its sequential optimization strategy, in which only a single image is updated at a time. Interpolation-only methods require only one iteration to identify the highest energy image along the interpolated path.

#### 3.1.2 Analysis of Geometric Parameters

We also analyzed the link between the type of reaction and the efficiency and success of the tested methods using various metrics, including the system size, magnitude of activation barrier and imaginary frequency, lateness of the TS in the reaction path, as well as RMSD between the geometries of reactant and product (RMSD­(RS-PS)), to quantify geometrical change during the reaction, and RMSD between TS guess and the reference TS (RMSD­(TS_ref_ – TS_guess_)) to indicate the quality of the TS guess; see Section SI-4.7 for further details.

No noticeable trends were observed with the metrics, except with RMSD­(RS-PS) and RMSD­(TS_ref_– TS_guess_). The number of failing cases is lower than the number of successful and unconverged TSs found, which makes it difficult to draw strong conclusions. Nevertheless, most reactions with successful and unconverged TSs had RMSD­(RS-PS) values distributed below ∼0.5 Å while failures were associated with higher values (Figure 4a). A positive correlation was also observed (Figure 4b) between the RMSD­(RS-PS) and RMSD­(TS_*guess*_ – TS_*ref*_), suggesting that larger structural changes along the reaction make it harder to generate good TS guesses, thereby increasing the failure rate of the TS search.

**Figure 4:**
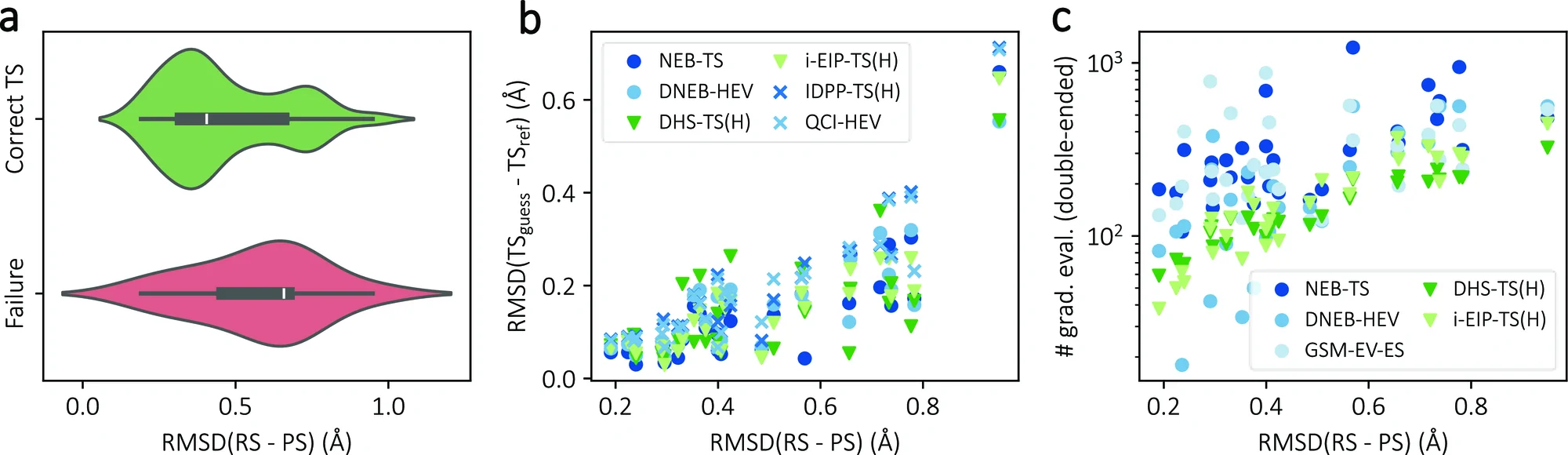
Impact of RS and PS geometries on TS search: (a) Distribution of RMSD (RS - PS) for runs that found correct TSs (success and unconverged) vs failures. (b) Correlation between RMSD­(RS - PS) and RMSD­(TS_guess_ – TS_ref_). (c) Correlation between RMSD­(RS – PS) and the number of gradient evaluations during double-ended step. *Methods omitted:* (b) GSM-EV-ES – TS guess not available in output, (c) IDPP-TS­(H) and QCI-HEV - Constant no. of gradient evaluations for double-ended step, (b and c) NEB-TS­(H) - identical to NEB-TS.

Larger RMSD­(RS-PS) also increases computational cost, especially in the double-ended step, evidenced by the correlation with the number of gradient evaluations in that step (Figure 4c). The correlation is stronger for bracket methods (i-EIP-TS­(H) and DHS-TS­(H)) compared to the chain-of-states methods, which show a larger spread of values. The reason for this difference is unclear; one possibility is that for chain-of-states methods, good interpolation can reduce the number of gradient evaluations. However, the quality of interpolation does not directly depend on the RMSD­(RS-PS).

Overall, our analysis indicates that the similarity of the end-point geometries correlates with both the success and the computational expense of double-ended TS search. This suggests that when the RS and PS geometries are similar, finding the TS using double-ended methods is easier compared to when larger RMSD (RS-PS) values are observed. In the latter case, a more careful evaluation of the double-ended methods and their parameters is needed, possibly even at the expense of a more costly computational approach.

### 3.2 Further Analysis of Benchmark Reactions

#### 3.2.1 Failures in TS Search

We analyzed reactions where one or more methods fail to identify the TS. In most cases, these methods returned TSs associated with unproductive imaginary modes (e.g., low frequency molecular “breathing”, or methyl rotation). Alternatively, they converged to a minimum with no imaginary modes or did not converge to a meaningful stationary point.

An unusual case is Reaction 8, which involves C–F activation with a niobium catalyst (Table 1). In this case, all tested methods failed to obtain the reference single-step TS, which has a barrier of 37.7 kcal mol^–1^ (ω_*i*_ = −359.9 cm^–1^) at the benchmark level of theory. The reaction was extracted from the MOBH35 data set,^53^ which in turn obtained it from a study by Gianetti et al., who also reported only the single-step mechanism.^79^ Interestingly, out of the eight hierarchical methods tested, NEB-TS, NEB-TS­(H), DHS-TS­(H), i-EIP-TS­(H), and QCI-HEV converged to valid saddle points associated with the breaking of the C–F bond but were lower in energy compared to the reference TS.

For example, the NEB-TS (ω_*i*_ = −263.2 cm^–1^) is 26.9 kcal mol^–1^ lower in free energy compared to the reference TS. An IRC pathway calculation showed that this TS connects a conformer of the reference PS on one end and leads to an intermediate (not the RS) on the other. By repeatedly applying CI-NEB and P-RFO, we discovered a two-step mechanism starting from an RS conformer (1.2 kcal mol^–1^ lower than the reference RS) and ending at a PS conformer (6.7 kcal mol^–1^ higher than the reference PS). The first TS in the two-step path, **TS1** (Δ*G* ^‡^ = 23.8 kcal mol^–1^) connects the RS conformer (Δ*G* = 0 kcal mol^–1^) to an intermediate **INT** (Δ*G* = 7.0 kcal mol^–1^), and **TS2** (Δ*G* ^‡^ = 12.0 kcal mol^–1^) connects this intermediate to the PS conformer (Δ*G* = −44.4 kcal mol^–1^). Relative to the RS conformer, the single-step, reference TS has a Δ*G* ^‡^ of 38.9 kcal mol^–1^, indicating that the two-step pathway is energetically favored (Figure 5).

**Figure 5:**
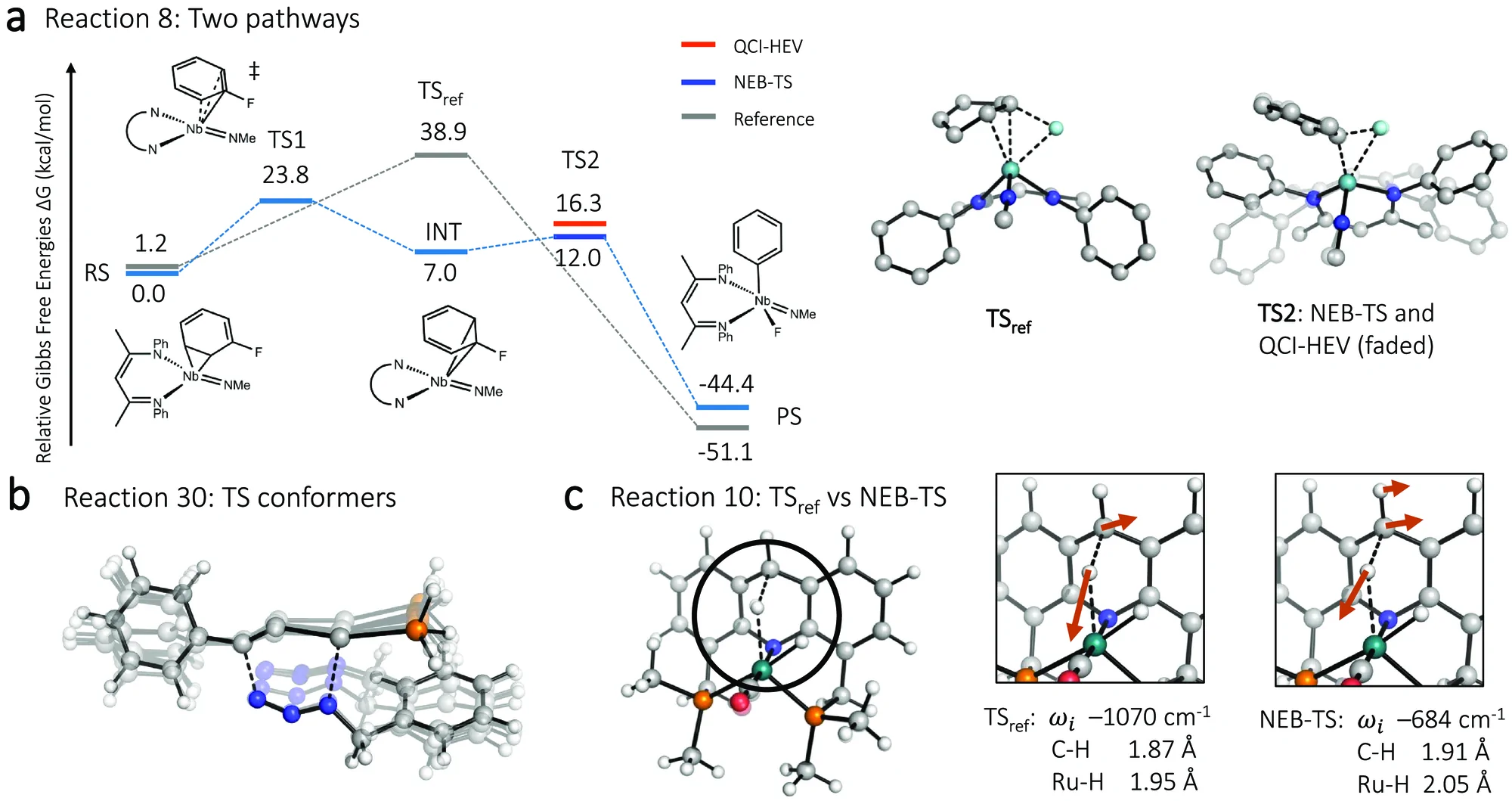
Further analysis of Reactions 8, 10, 30. (a) Energy diagram showing the different pathways for reaction-8, with TSs from NEB-TS and QCI-HEV indicated with blue and red, respectively, in the two-step path. The reference TS and the single-step path are shown in gray. The geometries of the reference TS, as well as a superposition of the NEB-TS and QCI-HEV (faded) TSs, are shown on the right. (b) TS conformers (faded) for reaction 30, from IDPP-TS­(H), GSM-EV-ES, DHS-TS­(H), i-EIP-TS­(H), NEB-TS, NEB-TS­(H) and QCI-HEV superimposed against ref TS (solid). (c) Superposition of the reference TS (solid) and NEB-TS (faded) geometries for reaction 10 (left) and significant normal mode displacement vectors shown with red arrows for both TSs (right).

DHS-TS­(H) and i-EIP-TS­(H) also converged to **TS2**, both differing by less than 0.04 kcal mol^–1^ in electronic energy, and 0.01 Å RMSD, compared to NEB-TS. The NEB-TS­(H) geometry was not completely converged since it had two imaginary frequencies (ω_*i*_ = −263.8 and −51.8 cm^–1^); however, it closely resembled the TS2 identified with NEB-TS (RMSD = 0.04 Å).

The TS from QCI-HEV was 4.3 kcal mol^–1^ higher in free energy than **TS2** from NEB-TS (16.3 kcal mol^–1^ free energy barrier), with IRC analysis confirming it is a conformer of **TS2**.

Note that we cannot justify counting these cases as failures since TS2 is part of a chemically valid pathway. These cases also cannot be counted as successes since TS2 does not connect the reactant and product in an elementary step, which is inconsistent with the rest of the set. Therefore, we have decided to remove it from the statistical analyses performed on the benchmark set.

These results also highlight the difficulty in comparing different TS search algorithms in terms of their success and failure rates when multiple pathways exist between an RS and a PS. Nevertheless, none of the other failing reactions in our benchmark arise from chemically meaningful secondary pathways, which means that our comparison of double-ended methods is robust in the majority of TM-catalyzed reactions.

#### 3.2.2 TS Conformers in Benchmark Results

As mentioned in Section-2.3, the RS and PS geometries were obtained by minimizing the IRC end-points from the reference TS. This approach is expected to ensure that double-ended methods converged to the same reference TS, as no conformer sampling was performed. However, of the (201 + 19 = ) 220 TSs, including successful or unconverged, we identified 14 cases where a conformer of the reference TS was returned. For DHS-TS­(H), there were four instances of TS conformers, for QCI-HEV, three; for NEB-TS and IDPP-TS­(H) two; for NEB-TS­(H), GSM-EV-ES, and i-EIP-TS­(H), one each; and none for DNEB-HEV.

For Reaction 30, which corresponds to Ag-catalyzed alkyne–azide cycloaddition,^78^ all successful methods (NEB-TS, NEB-TS­(H), GSM-EV-ES, i-EIP-TS­(H), DHS-TS­(H), IDPP-TS­(H) and QCI-HEV) found TS conformers of the reference TS. DNEB-HEV failed to produce a valid TS. For NEB-TS, NEB-TS­(H) and GSM-EV-ES, we observed additional weak imaginary frequencies (i.e., unconverged). Therefore, we reoptimized them in ORCA to obtain tightly converged geometries. The six distinct TSs (from the six methods) arise from differing orientations of the phosphine ligand on Ag and the benzene ring on the azide molecule. These conformers are close in energy and correspond to the same reaction, as confirmed by IRC calculations (Figure 5b), with the lowest energy TS coming from reoptimized NEB-TS­(H) and GSM-EV-ES geometries (Δ*G* ^‡^ = 19.0 kcal mol^–1^ relative to the reference RS), 0.1 kcal/mol lower than the reference TS (Δ*G* ^‡^ = 19.1 kcal mol^–1^), and the highest one from QCI-HEV (Δ*G* ^‡^ = 19.4 kcal mol^–1^).

Our results demonstrate that even if started from the same RS and PS, different methods can locate distinct but valid TS geometries, especially for flexible systems. This highlights the importance of performing conformer sampling within double-ended TS search approaches.

#### 3.2.3 Effect of Empirical Dispersion

In analyzing reaction 10, a Ru-catalyzed hydride transfer to an acridine pincer complex,^80^ we found that none of the TSs met the criteria outlined in Section-2.4. All double-ended TSs showed a first eigenvector overlap of 0.77 with the reference TS (below the threshold of 0.9), despite their geometries being similar (RMSD of 0.03 Å).

The reference TS optimized at the PBE0-D3BJ/def2-SVP level of theory has one imaginary frequency of −1070 cm^–1^, while the NEB-TS obtained at the same level of theory had an imaginary frequency of only −684 cm^–1^. Visualization of the imaginary mode reveals that for NEB-TS there is additional movement in the C–H moiety accepting the hydride, in contrast to the reference TS, for which only the transferring H and accepting C predominantly move (Figure 5). Therefore, they appear to represent distinct saddle points on the PES, despite being close in energy (ΔΔ*E* ^‡^ = 1.0 kcal mol^–1^) and leading to the expected RS and PS following IRC calculations.

To understand this difference, we reoptimized both TSs with and without dispersion corrections and with different functionals. At the PBE0/def2-SVP level of theory without dispersion, the two TSs collapse into a single TS (ω_*i*_ = −1084 cm^–1^). Using ωB97X/def2-SVP also returns the same TS in both cases (ω_*i*_ = −1355 cm^–1^). However, optimizing with ωB97X-D3BJ/def2-SVP yielded two distinct TSs with imaginary frequencies of −1256 and −907 cm^–1^ for the reference and NEB-TS, respectively. Using the nonlocal VV10 dispersion model,^81^ which has been shown to perform well for organometallic reactions,^53^ with the PBE0 functional resulted in a single TS (ω_*i*_ = −1053 cm^–1^), suggesting that the differences stem from the use of the D3­(BJ) dispersion model. While dispersion corrections are generally needed for accurate energies and geometries, they may lead to issues in the PES. As noted by Head-Gordon and others, using D3, D3­(BJ), or D4 corrections can lead to unphysical minima and incorrect vibrational frequencies, particularly for organometallic systems.^82,83^ The origin of these artifacts stems from the dependence of dispersion energies on the coordination number, which leads to sharp jumps in the energy with small changes in geometry. The VV10 dispersion uses only the electron density and thus does not suffer from this issue. This example illustrates the importance of evaluating the effect of dispersion on TS search, as it can result in spurious saddle points on the PES. Although beyond the scope of this study, a more systematic analysis of such effects is encouraged.

#### 3.2.4 Choice of Method Settings

In this study, we focused on evaluating the performance of various hierarchical double-ended methods on metal systems, with recommended or default settings that were tuned primarily on organic reactions. For example, for GSM-EV-ES, the parameters were tested on 72 organic reactions.^19^ Using 9 intermediate images and default settings, a total of 468 gradients were required to locate all TSs successfully. In our case, with 8 intermediate images, 326 gradients were required, resulting in just one failure.

The default settings for NEB-TS and NEB-TS­(H) were tuned on a set of 121 main-group organic reactions, primarily single-step.^40^ For NEB-TS­(H), these default settings correspond to 8 intermediate images (excluding end-points), and a convergence criterion of 1 × 10^–3^ *E*_h_ *a*_0_^–1^ for root-mean-squared (RMS) force and 2 × 10^–3^ *E*_h_ *a*_0_^–1^ for maximum component of force on the climbing image. For other images in the path, the convergence thresholds are 1 × 10^–2^ *E*_h_ *a*_0_^–1^ for RMS and 2 × 10^–2^ *E*_h_ *a*_0_^–1^ for the maximum component of perpendicular force. With these settings, 94% of the TSs were found within 0.5 kcal mol^–1^ of the reference, indicating success.^40^ The CI-NEB step and the TS optimization step required an average of 501 and 33 gradient evaluations, respectively. In our case, the CI-NEB step required 361 gradients and the TS optimization 69 gradient evaluations.

It is difficult to directly compare results between the data sets of organic reactions and our smaller set of 30 metal-catalyzed reactions, especially when different levels of theory are applied. In both GSM-EV-ES and NEB-TS­(H) the number of gradient evaluations is within the same order of magnitude for both organic and metal sets. The values are slightly lower for our reaction set, which might be due to the use of minimized IRC end-points as RS and PS. This guarantees that there is only one smooth barrier between the RS and PS. Overall, the parameters tuned on organic reactions prove effective when applied to the reactions on our data set. This is in contrast to Ruttinger et al., who tuned the parameters for the NEB method on a wide range of reactions, including the MOBH35 data set. They found that the performance on metal reactions was relatively poor compared to other organic reactions.^84^ However, the difference might be attributed to the much improved efficiency of the EW-CI-NEB, as well as the use of linear interpolation by Ruttinger et al. in starting the NEB. The IDPP method, used with EW-CI-NEB in our case, is known to provide better starting paths than simple linear interpolation.^36^

We recognize that parameter fine-tuning can potentially improve performance. However, exhaustive testing of multiple parameter combinations for every method is outside the scope of this study. Consequently, we selected the NEB-TS­(H) approach as a representative method to explore the impact of parameter choices, as it achieved the highest success rate in our benchmark (no failures), and it is a popular method.^57–59^ To explore this more systematically, we conducted three sets of tests:

i) Varying the number of CINEB images: Because the computational cost of NEB-TS­(H) scales with the number of images in the double-ended (CI-NEB) step, we investigated the effect of reducing the number of intermediate images from eight to five (excluding the fixed reactant and product end points). Using five images led to reaction-6 failing, compared to none originally. Additionally, it increases the average number of gradient evaluations in the CI-NEB step, from 361 to 480 (Figure 6a). These results suggest that reducing the number of images degrades the resolution of the reaction path and consequently the convergence of CI-NEB, increasing the overall computational cost.
ii) Varying CINEB convergence thresholds: We doubled (i.e., loosened) all gradient convergence thresholds from their default values mentioned above, i.e., 2 × 10^–3^ *E*_h_ *a*_0_^–1^ and 4 × 10^–3^ *E*_h_ *a*_0_^–1^ for RMS and max. force on CI and 2 × 10^–2^ *E*_h_ *a*_0_^–1^ for RMS and 4 × 10^–2^ *E*_h_ *a*_0_^–1^ for max. perpendicular force on other images. We kept all other settings the same, including using 8 intermediate images. Loosening the convergence threshold did not degrade the success rate, showing no failures on the data set (Figure 6a). In fact, it resulted in a significant decrease in the computational cost of the double-ended step by 40%, from 361 to 214 gradient calls on average. This indicates that using coarser convergence criteria can effectively reduce computational cost without degrading performance. This is expected since the TS guess from CI-NEB only needs to be sufficiently close to the true TS for its first mode to correspond to the correct reaction coordinate. This need for proximity can be satisfied even if the path is not fully converged.
iii) Energy-weighted vs uniform-spring version of CI-NEB: We compared the default energy-weighted CI-NEB in ORCA with the uniform-spring version. The energyweighted spring scheme^40^ allows images to accumulate near high-energy regions of the path, thus improving resolution. While this is the default in ORCA, other software, such as ASE,^85^ use the uniform-spring version. Our results show that using uniform spring constants instead of energy-weighting heavily degrades the performance of NEB-TS­(H), resulting in eight failed reactions. The computational cost is also much higher, around 863 gradients on average (Figure 6a). The issues in the uniform spring CI-NEB arise from cases where the CI-NEB calculation failed to converge, resulting in a large number of gradient evaluations. We suggest that energy-weighted springs are used with CI-NEB whenever possible to improve convergence and success rates.

**Figure 6:**
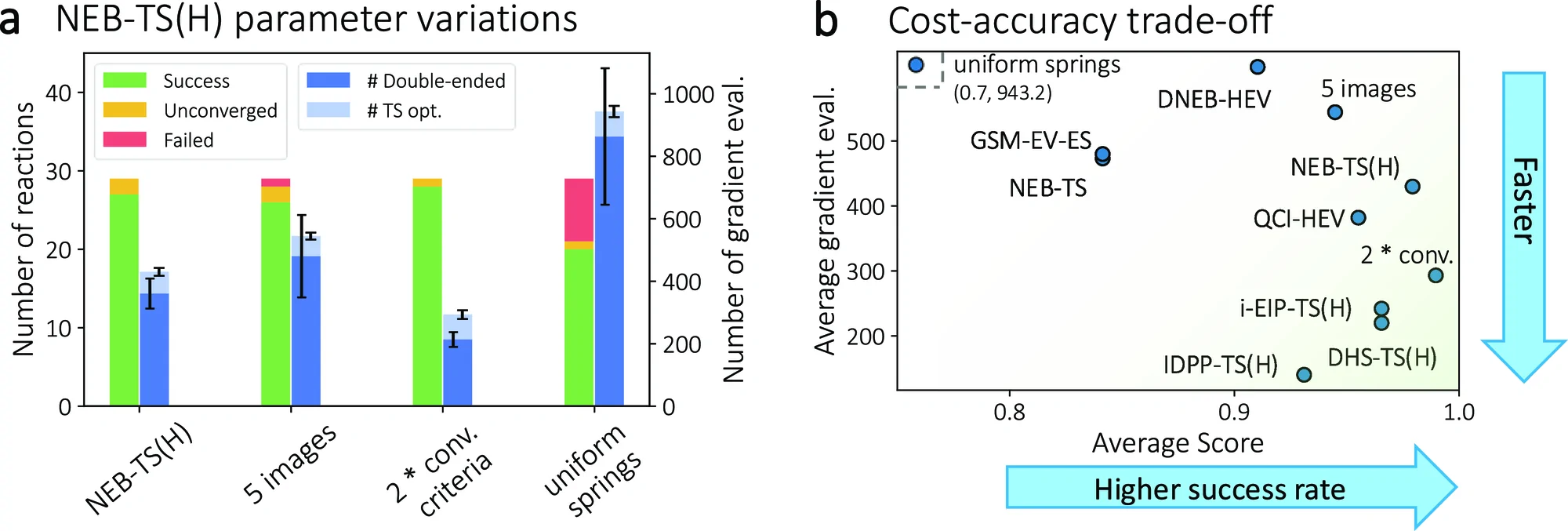
(a) Success rates and average number of gradient evaluations for NEB-TS­(H) under different settings, varying one parameter at a time (from left to right): (i) default setting with 8 images, (ii) 5 images (iii) 8 images but convergence thresholds relaxed by a factor of 2, (iv) uniform springs instead of energy-weighted springs. Reported image numbers exclude the fixed reactant and product end points. (b) Average number of gradient evaluations versus success score for various methods including the variations described above for NEB-TS­(H). The Pareto optimal region is shaded in green.

Overall, using only coarser convergence criteria is a valuable strategy for reducing the computational cost without degrading the success rate.

#### 3.2.5 Cost-accuracy Trade-off

To compare the performance of all methods tested, including the variations of NEB-TS­(H), we analyzed the trade-off between success rate and computational cost. We defined a success score of 1 for a successfully located TS, 0.7 for an unconverged TS, and 0 for failures. Since a robust TS search workflow ultimately requires obtaining converged TSs, unconverged cases are penalized despite not being complete failures. The score is averaged over the benchmark set to yield a normalized performance metric for each method. Since the score assigned to unconverged TSs is inherently subjective, we repeated the analysis using values of 0.6 and 0.8 (Figure S4 in the SI). Although changing this value slightly alters the relative positions of some methods, the overall conclusions remain unchanged.

Comparing the average number of (Hessian-corrected) gradient evaluations against the success score provides an indication of cost-accuracy trade-off (Figure 6b). In the lower-right part of the plot, corresponding to high success rate and low computational costs, are IDPP-TS­(H), DHS-TS­(H), i-EIP-TS­(H), QCI-HEV and NEB-TS­(H) with coarse convergence settings. In contrast, GSM-EV-ES and NEB-TS have suboptimal performance, arising primarily from the poor performance of the TS optimizer as discussed above. NEB-TS­(H) with uniform spring also has poor performance due to convergence failures in the double-ended step. DNEB-HEV achieves a good success rate but is significantly slow, and therefore computationally more costly than the other methods tested. Overall, IDPP-TS­(H), DHS-TS­(H), i-EIP-TS­(H) and NEB-TS­(H) with coarse convergence emerge as optimal choices, while QCI-HEV and NEB-TS­(H) with default settings also perform well but are more computationally expensive.

### 3.3 Case Studies

The benchmark study above focused on idealized cases in which the RS and PS geometries were generated by following the IRC path downhill from the known TS, ensuring a single-barrier (i.e., elementary) reaction and well-aligned RS and PS conformations. In a practical scenario, however, reaction pathways may involve multiple elementary steps and/or conformational changes. Therefore, to evaluate the performance of hierarchical double-ended methods under more realistic conditions, we consider two representative TM-catalyzed reactions as case studies - (i) multistep CH activation of benzene catalyzed by Pd­(OOCH)_2_,^86^ and (ii) predicting the regioselectivity of an Ir-catalyzed borylation reaction.^87^

#### 3.3.1 Multistep Pd-catalyzed C–H Activation

This multistep reaction was computationally studied by Biswas et al.,^86^ and they identified a two-step mechanism. Initial 3D geometries were drawn in Avogadro 1.2.0 ^89^ and optimized using ORCA at the PBE0-D3BJ/def2-SVP level of theory, which was used throughout. Starting from the optimized RS and PS, hierarchical double-ended methods were applied to locate one TS, then the IRC was followed in both directions to obtain the intermediate(s), which were then used for further iterative double-ended runs to find the other TSs until the complete path between RS and PS was found. We have tested only the methods with high success rate and good computational efficiency i.e., IDPP-TS­(H), DHS-TS­(H), i-EIP-TS­(H), QCI-HEV and NEB-TS­(H) with default and doubled convergence thresholds.

All methods, except NEB-TS­(H), located three TSs along the reaction path. These included the two TSs reported by Biswas et al. and a third TS corresponding to the conformational change between *syn*- and *anti*-orientations of the carboxylate −OH in the product. This additional barrier arises because the drawn PS geometry corresponds to the higher-energy *anti*-carboxylate conformation whereas the reported PS in Biswas et al.^86^ had the lower energy *syn-*orientation. Surprisingly, this conformational barrier is higher than the other two TSs, highlighting the necessity of conformational sampling, which was not considered here.

The order in which TSs were identified differed across methods. IDPP-TS­(H), i-EIP-TS­(H) and QCI-HEV found the C–H activation TS (**TS**_**B**_) in the first run (Figure 7a), partitioning the path into two remaining elementary steps. In contrast, DHS-TS­(H) and NEB-TS­(H) first identified the conformer interconversion peak (**TS**_**C**_) in the first run. For NEB-TS­(H), successive runs failed to find the other TSs, which is surprising, given that the simpler IDPP-TS­(H) approach successfully identified all TSs. Inspection of NEB-TS­(H) trajectories revealed that the TS guess generated during the second run had the correct imaginary mode corresponding to the partial acetate dissociation barrier (**TS**_**A**_), yet the subsequent optimization failed to converge to this TS. Instead, it converged to an alternative TS in which the dissociated acetate lies away from the C–H bond, and is therefore not suited for the C–H activation (Figure 7b). The same issue was observed when NEB-TS­(H) was used with coarser convergence thresholds.

**Figure 7:**
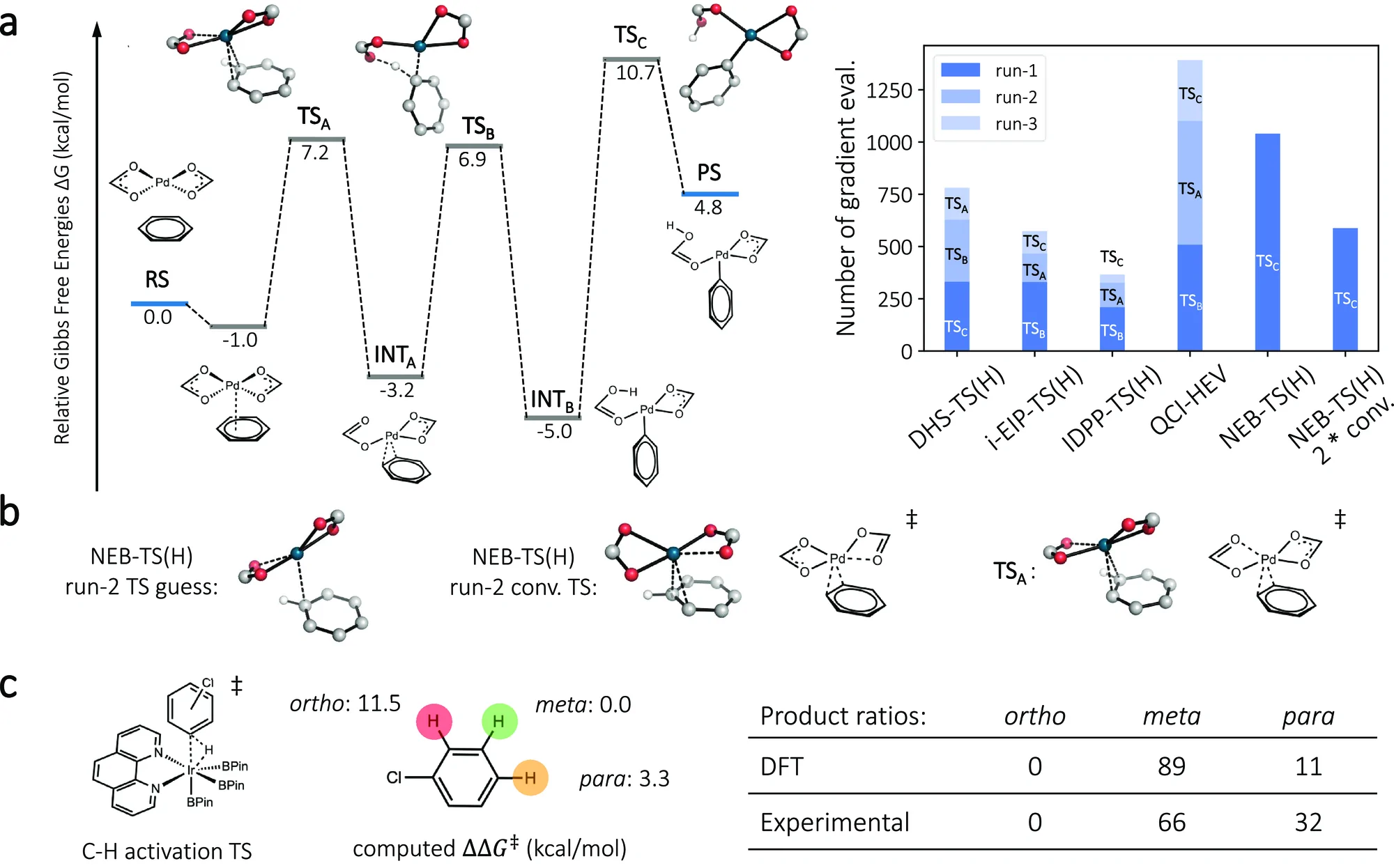
**Case studies**. (a) (left) Pd-catalyzed benzene C–H activation - reaction free energy profile computed at the PBE0-D3BJ/def2-SVP level and (right) number of gradient evaluations for the TS searches through different double-ended methods. (b) The TS guess and optimized TS from NEB-TS­(H) (left and middle) compared to **TS**_**A**_ (right) showing the bonds forming and breaking. (c) The relative Gibbs free energies of activation and product ratios (assuming kinetic control) for the Ir-catalyzed borylation of chlorobenzene at the *ortho*-, *meta*- and *para*- positions, compared to separated catalyst and reactant; computed at B2K-PLYP/def2-TZVP//B97-D3/def2-SV­(P) level of theory with CPCM­(THF) solvation at 80 °C.

The origin of this failure of NEB-TS­(H) might be due to the different order in which the TSs were found. In this case, **TS**_**C**_ is located first, meaning that the subsequent search must consider a longer pathway with two barriers between **INT**_**B**_ and RS (Figure 7a), which is challenging. In addition, the **TS**_**A**_ has a weak imaginary mode (−125.8 cm^–1^), indicating a shallow PES compared to the other two TSs (**TS**_**B**_ −754.9 cm^–1^ and **TS**_**C**_ −622.4 cm^–1^). Converging TSs with such low PES curvature is more difficult, especially when the Hessian is calculated only once at the start of the optimization, as is the case here.

Although both NEB-TS­(H) and DHS-TS­(H) find **TS**_**C**_ in the first run, the subsequent searches proceeded differently. The second NEB-TS­(H) run generated a guess corresponding to **TS**_**A**_, while DHS-TS­(H) generated a guess closer to **TS**_**B**_. As a result, DHS-TS­(H) successfully identified all remaining TSs, while NEB-TS­(H) did not. However, tightening the CI-NEB convergence thresholds in NEB-TS­(H) to half of the default, enabled the successful location of **TS**_**A**_, indicating that the failure arises from the quality of the TS guess and the reliability of the optimization rather than the ability of the method to identify the correct path. This example shows that the parameters required for successful TS search may vary with different reactions, especially multistep reactions, encouraging further work in this area. Moreover, it also highlights the utility of using a range of double-ended methods instead of relying on a specific choice.

The computational cost for finding each TS (Figure 7a) was evaluated in terms of gradient evaluations (including corrections for Hessian calculations). Among the tested methods, IDPP-TS­(H) is the most efficient, requiring a total of 366 gradient evaluations. It is followed by the bracket methods i-EIP-TS­(H) and DHS-TS­(H) with 574 and 781 gradient evaluations, respectively, while QCI-HEV is the most computationally demanding (1392 gradients). These results are consistent with the benchmark results for elementary reactions. In most cases, the computational cost of the second or third run is lower than that of the first run, reflecting the shorter path lengths involved. This is consistent with our findings in Section-3.1.2.

Overall, this case study demonstrates that hierarchical double-ended methods can successfully recover multistep reaction mechanisms, even in the presence of additional conformational barriers. The consistency of computational cost with the benchmark set further supports the generality of our results.

#### 3.3.2 Ir-catalyzed Borylation

We next consider the Ir-catalyzed borylation of aryl C–H bonds, an important transformation for functionalization of arenes. Norrby and Hartwig have reported a hybrid semiempirical (GFN2-xTB) and machine learning (ML) framework to predict regioselectivity ratios for this reaction.^87^ While fast once trained, such models rely on pre-existing data sets of TS and/or activation barriers. Here we test whether double-ended methods can provide a direct and rapid prediction of regioselectivity. As a test case, we considered the borylation of chlorobenzene with Ir­(Me4Phen)­(BPin)_3_ catalyst, for which an experimental *meta*:*para* selectivity ratio 66:32 was reported, with no *ortho-*product.^87^

Reactant complexes were generated by placing a chlorobenzene molecule in close proximity to the catalyst. Product geometries were obtained in an automated workflow by enforcing distance constraints to force the breaking of the C–H bond and the formation of the Ir–H and Ir–C bonds (see Section SI5.2 for further details). Given the system size and the requirement for computational efficiency, we used the IDPP-TS­(H) method, identified as the most efficient in our benchmark. Calculations were performed at the CPCM­(THF)­B2K-PLYP/def2-TZVP//CPCM­(THF)­B97-D3/def2-SV­(P) level of theory,^67,71,90,91^ also chosen keeping in mind the efficiency of B97-D3, and good performance of B2K-PLYP in calculating organometallic barrier heights.^53^ Using the default IDPP-TS­(H) settings, the *ortho*-TS could not be converged. In addition, the optimized *para*-TS had two imaginary frequencies (−687 and −8 cm^–1^). Correct TSs with single imaginary modes were only obtained when the Hessian was recalculated every 20 steps during the P-RFO optimization. Therefore, this modified protocol was used to obtain the TSs. The computed activation barriers for CH activation, which is irreversible and determines the regioselectivity, were 39.3, 22.2, and 23.8 kcal mol^–1^ at the *ortho*, *meta* and *para* positions, respectively (Figure 7c).

Assuming kinetic control, the computed *meta*:*para* selectivity (derived from *Δ*Δ*G* ^‡^) is 89:11, in qualitative agreement with the experimental ratio of 66:32.^87^ The calculated fraction of *ortho* product is negligible (ΔΔ*G* ^‡^ = 11.5 kcal mol^–1^) which also matches the experimental results.

This example demonstrates that while we were successful in the qualitative prediction of selectivity with hierarchical double-ended methods, adjustments to default parameters may be necessary in cases involving more complex surfaces. Automating this approach further could make this strategy useful for mechanistic analysis as well as for generating training data for ML models in catalyst optimization.

## 4 Conclusion

In this study, we systematically benchmarked the out-of-the-box performance of eight hierarchical double-ended TS search methods for metal-catalyzed reactions in terms of both success rates and computational costs.

Most workflows achieved high success rates on the benchmark set. IDPP-TS­(H), DHS-TS­(H), i-EIP-TS­(H), QCI-HEV, NEB-TS­(H) and DNEB-HEV all achieved high success rates, indicating that modern double-ended approaches are generally robust for locating TSs in single-step organometallic reactions. Only GSM-EV-ES and NEB-TS showed lower success rates. Most GSM-EV-ES failures arose from unconverged TS optimizations rather than unsuccessful path generation, suggesting that further assessment using alternative TS optimizers may be required to fully assess this approach. The lower performance of the NEB-TS primarily arose from failures in the TS optimization, highlighting the importance of using calculated Hessian for TS refinement. Because success rates were similar for most methods, computational efficiency provided the most meaningful basis for comparison. IDPP-TS­(H) emerges as the most efficient method overall, with the bracket methods DHS-TS­(H) and i-EIP-TS­(H) also showing competitive performance.

Beyond the performance ranking, the benchmark revealed several practical insights:

(i) success in the TS refinement stage depends strongly on the use of accurate Hessian (or explicitly calculated lowest Hessian eigenvalue and eigenvector).
(ii) bracket methods, such as i-EIP-TS­(H), DHS-TS­(H), and interpolation-only methods (IDPP-TS­(H), QCI-HEV) can be more efficient than widely used methods like NEB-TS­(H) or GSM-EV-ES.
(iii) different workflows may converge to different TSs, even when all methods start from identical reactant and product geometries, highlighting the value of applying multiple methods when exploring complex PESs.
(iv) reactions that involve larger structural changes between reactant and product geometries, as quantified by RMSD­(RS-PS), are generally associated with lower success rate and higher computational costs.
(v) empirical dispersion corrections can occasionally give rise to spurious saddle points (reaction 10), requiring further investigation on their use in TS optimization.

To evaluate potential gains from parameter optimization, we evaluated the influence of the number of images, convergence criteria and the use of energy-weighted springs in the CI-NEB step for NEB-TS­(H), noting that the default settings were originally developed for organic reactions. Reducing the number of images and using uniform spring constants reduced the success rate and increased the computational cost. In contrast, using coarser convergence criteria reduced the overall computational cost by 40% without affecting success rates. Considering both reliability and efficiency, IDPP-TS­(H), DHS-TS­(H), i-EIP-TS­(H) or NEB-TS­(H) with coarser convergence criteria offer the most favorable cost-accuracy trade-offs.

Although these findings are based on a relatively modest benchmark of elementary organometallic reactions, they provide practical guidance for selecting double-ended TS search methods. Validation on a larger and more diverse test set, particularly incorporating multistep reactions, will be important to assess the generality of observed trends.

Finally, two case studies demonstrate the practical use of these methods to more complex reactions, including a multistep Pd-catalyzed C–H activation process. These examples illustrate how hierarchical double-ended methods can be incorporated into semiautomated reaction modeling workflows, also highlighting current limitations. In particular, the examples demonstrate the importance of conformational sampling for both minima as TSs, which is relevant for flexible systems. A promising avenue to improve performance is the use of machine learned interatomic potentials (MLIPs), both in the double-ended and TS refinement steps, which has been reported to increase efficiency with NEB and DE-GSM.^43,92^

While several challenges remain, our benchmark shows that hierarchical double-ended methods can be applied successfully to metal-catalyzed reactions. In particular, less widely used methods, like DHS or i-EIP, provide a competitive and often more efficient alternative than popular methods like CI-NEB. We hope that our benchmark provides useful guidance for the selection of double-ended methods for TS search and further development of TS search method.

## Supplementary Material



## Data Availability

The Python package autodE is an open-source package, freely available at https://github.com/duartegroup/autodE. The coordinates of the optimized geometries and input files for all ORCA calculations can be accessed via 10.19061/iochem-bd-6-566.

## Notes

The Supporting Information includes (1) details of parameters and settings used for each method, including convergence criteria and template input files for molecularGSM and OPTIM, (2) details on the autodE implementation of DHS and i-EIP, (3) further details on benchmark set, including choice of reactions, modifications (showing reaction schemes) and the procedure for generation of RS and PS geometries, (4) detailed benchmark results: automated and manual assignments of TSs, showing heatmaps of assignments, TS data used for assignment, raw data for efficiency analysis, further results for NEB-TS­(H) parameter choices, discussion on choice of success scores, energy and thermochemical data on TS conformers, data and analysis plots of various reaction descriptors, (5) additional information on case studies, including energies and thermochemical data on all stationary points, and procedure for preparation of RS/PS geometries and calculation of rate constant ratio for Ir-borylation (PDF)
