# Supplementary material for: Benchmarking Hierarchical Double-Ended Transition-State Search Methods for Metal-Catalyzed Reactions
Source: J Chem Theory Comput. 2026 Sep 25;22(19):10227–43. doi: 10.1021/acs.jctc.6c00992 (PMC13671179; doi:10.1021/acs.jctc.6c00992)
Supplement: Supplementary file 1 [file ct6c00992_si_001.pdf]

Supplementary Information: Benchmarking hierarchical double-ended transition state search methods for metal-catalyzed reactions

Shoubhik R. Maiti,† David Buttar,‡ and Fernanda Duarte*,†

†*Department of Chemistry, University of Oxford, Mansfield Road, Oxford OX1 3TA, United Kingdom*

‡*Data Science and Modelling, Pharmaceutical Sciences, R&D, AstraZeneca, Macclesfield*

E-mail: fernanda.duartegonzalez@chem.ox.ac.uk

1 Description of the methods and packages

A brief description of the methods used and the settings for each package is given below. Note that the number of TS refinement steps is constrained to a fixed number in each case to prevent non-convergent cases from skewing the statistics.

1.1 NEB-TS

NEB-TS calculations were conducted in ORCA v5.0.4. We use the keyword `NEB-TS` to invoke the hierarchical double-ended method. Default settings were used for the CI-NEB part — which sets 8 images (excluding end points) and convergence criteria of $0.001\,E_\mathrm{h}\,{a_0}^{-1}$ for the root mean squared (RMS) gradient and $0.002\,E_\mathrm{h}\,{a_0}^{-1}$ for the maximum gradient component perpendicular to the NEB path acting on the climbing image (highest energy image) For other images, the convergence settings were $0.01\,E_\mathrm{h}\,{a_0}^{-1}$ RMS forces and $0.02\,E_\mathrm{h}\,{a_0}^{-1}$ maximum

component of forces.In the P-RFO TS optimization, the maximum number of iterations was set to 300. For the optimization, the maximum component of each step was constrained to 0.1 in internal coordinate units. This disallows large step sizes, which may hamper the convergence of the optimizer.

1.2 DNEB-HEV

The combined algorithm is invoked with the keywords `QCI` and `NEWCONNECT` for interpolation using the QCI algorithm and DNEB path calculation, respectively, and `BFGSTS` for HEVF TS optimization. The number of images for both QCI and DNEB was set to 8. For DNEB, the maximum number of iterations was set to 70, and the spring constant was set to $1.0\,E_\mathrm{h}\,\text{Å}^{-2}$. The spring constant was adjusted every 20 steps by a factor of 1.03 to keep the average deviation of interimage distances $< 2.0\%$ (`ADJUSTK 20 2.0 1.03`). The convergence criterion for the DNEB optimization was set to $5 \times 10^{-3}\,E_\mathrm{h}\,\text{Å}^{-1}$ for the RMS forces on the entire path of images.

For HEVF, each step was allowed to use a maximum of 60 gradient evaluations to calculate the *min-mode* by finite difference. The eigenvalue was considered converged when the RMS forces perpendicular to the eigenvector was less than $1 \times 10^{-3}\,E_\mathrm{h}\,\text{Å}^{-1}$ and the change in eigenvalue between two steps was less than 1%. The maximum step size along the *min-mode* was set to 0.2 Å, and along all perpendicular directions to 0.1 Å. The maximum number of optimizer iterations was set to 70, since each step uses multiple gradient evaluations (compared to other TS refinement algorithms used here).

1.3 GSM-EV-ES

The GSM-EV-ES calculations were run using the package molecularGSM (`https://github.com/ZimmermanGroup/molecularGSM`), using ORCA v5.0.4 for the energies and gradients. The original paper suggested using a total of 11 images (i.e., 9 intermediate images). Here, we use 8 intermediate images for consistency with other chain-of-states methods. The rec-

ommended RMS force convergence tolerance of $5 \times 10^{-4}\, E_\mathrm{h}\,\text{Å}^{-1}$ was used for the string optimization. For the final TS optimization, the convergence criteria were set to $5 \times 10^{-5}\, E_\mathrm{h}\,\text{Å}^{-1}$ for the RMS and $1 \times 10^{-4}\, E_\mathrm{h}\,\text{Å}^{-1}$ for the maximum component of the atomic forces to obtain tightly converged TSs. The maximum number of iterations for the TS optimization was set to 300.

1.4 DHS-TS(H)

The method is implemented in the `DHS` class in `autode.bracket.dhs` in autodE v1.4.3. The default options are used—which sets the size of the linear interpolation step to 0.2 Å when the distance between the image-pair is > 1.5 Å and to 0.05 Å otherwise. For the constrained optimization, the maximum step size was set to 0.1 Å or the interpolation step size, whichever is minimum. The method stops when the distance between two images is less than 0.5 Å (we noticed a tendency for one image to jump over the barrier if the distance convergence tolerance is set lower). The TS guess obtained from DHS was optimized with ORCA's P-RFO TS optimizer, using a tight optimization criterion and starting with one analytic Hessian calculation. The maximum step size was set to 0.1, and the maximum number of iterations was set to 300.

1.5 i-EIP-TS(H)

The i-EIP calculations were run with autodE v1.4.3 using the `IEIP` class in `autode.bracket.ieip`. The default convergence criteria are used as described in the original publication.[1] The TS guesses from i-EIP were optimized with ORCA's TS optimizer, using tight optimization criteria, 0.1 maximum step size, and 300 maximum iterations. An analytic Hessian is calculated to start the optimization. Compared to the original publication, our implementation does not check the overlap between the lowest Hessian mode and the image-pair vector (due to the cost of calculating the Hessian matrix). At the end, the TS optimization is started from the TS guess without any further checks. The P-RFO TS optimizer in ORCA is used,

with a tight optimization criterion, starting from an analytic Hessian, 0.1 maximum step size and 300 maximum iterations.

1.6 Summary of convergence criteria

Table S1: Convergence criteria for the hierarchical double-ended methods.

**Method**	**Double-ended**	**TS opt.**
NEB-TS	Max. iteration: 500<br>Climbing image:<br>$\mathrm{RMS}(g) < 0.001\,E_\mathrm{h}\,{a_0}^{-1}$<br>$\max(g) < 0.002\,E_\mathrm{h}\,{a_0}^{-1}$<br>Other images:<br>$\mathrm{RMS}(g^\perp) < 0.01\,E_\mathrm{h}\,{a_0}^{-1}$<br>$\max(g^\perp) < 0.02\,E_\mathrm{h}\,{a_0}^{-1}$	Algorithm: P-RFO<br>Max. iterations: 300<br>Max. step size: 0.1<br>$\Delta E < 1 \times 10^{-6}\,E_\mathrm{h}$<br>$\mathrm{RMS}(g) < 3 \times 10^{-5}$<br>$\max(g) < 1 \times 10^{-4}$<br>$\mathrm{RMS}(\Delta q) < 6 \times 10^{-4}$<br>$\max(\Delta q) < 0.001$
GSM-EV-ES	Max. iteration per step: 80<br>Climbing image:<br>$\mathrm{RMS}(g) < 5 \times 10^{-4}$	Algorithm: quasi-Newton<br>Max. iterations: 300<br>$\mathrm{RMS}(g) < 5 \times 10^{-5}$<br>$\max(g) < 1 \times 10^{-4}$
DNEB-HEV	Max. iteration: 70<br>All images:<br>$\mathrm{RMS}(g^\mathrm{DNEB}) < 0.005\,E_\mathrm{h}\,\text{Å}^{-1}$	Algorithm: HEVF<br>Max. iterations: 70<br>Max. step size: 0.2 Å<br>Max. Rayleigh-Ritz steps: 60<br>$\mathrm{RMS}(g^\perp)$ for lowest eigenvalue < 0.001<br>$\mathrm{RMS}(g) < 1 \times 10^{-4}\,E_\mathrm{h}\,\text{Å}^{-1}$<br>$\lVert\Delta x\rVert < 0.05\,\text{Å}$
DHS-TS(H)	Max. iteration: 1000<br>Distance-constrained minimisation:<br>$\mathrm{RMS}(g^\perp) < 0.001\,E_\mathrm{h}\,\text{Å}^{-1}$<br>Image-pair:<br>$R_\mathrm{tol} < 0.5\,\text{Å}$	Algorithm: P-RFO<br>Max. iterations: 300<br>Max. step size: 0.1<br>Same as NEB-TS
i-EIP-TS(H)	Max. iteration: 1000<br>Image-pair:<br>$\mathrm{RMS}(g^\perp) < 0.01\,E_\mathrm{h}\,\text{Å}^{-1}$<br>$R_\mathrm{tol} < 0.3\,\text{Å}$	Algorithm: P-RFO<br>Max. iterations: 300<br>Max. step size: 0.1<br>Same as NEB-TS
IDPP-TS(H)	Spring constant $= 1.0\,\text{Å}^{-4}$<br>$\max(g^\perp_\mathrm{IDPP}) < 0.01\,\text{Å}^{-3}$	Algorithm: P-RFO<br>Max. iterations: 300<br>Max. step size: 0.1<br>Same as NEB-TS

1.7 Template input files

The following is the template input file for GSM-EV-ES calculations in molecularGSM package. The template is based on examples provided in the GitHub repository of the package: `https://github.com/ZimmermanGroup/molecularGSM`.

```
# GSM-EV-ES input file (inpfileq)

------------- QCHEM Scratch Info ------------------------
$QCSCRATCH/    # path for scratch dir. end with "/"
GSM_go1q       # name of run
---------------------------------------------------------

------------ String Info --------------------------------
SM_TYPE                 GSM    # SSM, FSM or GSM
RESTART                 0      # read restart.xyz
MAX_OPT_ITERS           80     # maximum iterations
STEP_OPT_ITERS          30     # for FSM/SSM
CONV_TOL                0.0005 # perp grad
ADD_NODE_TOL            0.1    # for GSM
TS_FINAL_OPT            300   # final TS refinement maxiter
TS_CONV_TOL             0.00005 # final TS refinement tol rms grad
TS_GRAD_MAX_TOL         0.0001  # final TS refinement tol max grad
SCALING                 1.0    # for opt steps
SSM_DQMAX               0.8    # add step size
GROWTH_DIRECTION        0      # normal/react/prod: 0/1/2
INT_THRESH              2.0    # intermediate detection
MIN_SPACING             5.0    # node spacing SSM
BOND_FRAGMENTS          1      # make IC's for fragments
INITIAL_OPT             0      # opt steps first node
FINAL_OPT               150    # opt steps last SSM node
PRODUCT_LIMIT           100.0  # kcal/mol
TS_FINAL_TYPE           1      # any/delta bond: 0/1
NNODES                  10      # including endpoints
---------------------------------------------------------
```

To run `molecularGSM` an `ograd` file is required to drive ORCA jobs. The `ograd` file which we used is given below.

```
#!/bin/bash
# The path to ORCA should be added to .bashrc or exported in command line


if [ -z $2 ]
then
  echo " need two arguments! "
```

```
  exit
fi

# echo " in ograd: $1 $2 "
ofile=orcain$1.in
ofileout=orcain$1.out
molfile=structure$1
ncpu=$2

#echo " ofile: $ofile ofileout: $ofileout molfile: $molfile ncpu: $ncpu"
# keep a counter of the number of gradient calls
printf "*" >> total_grad_counter.txt

cd scratch/
########## DFT settings: ################
echo '! PBE0 D3BJ def2-SVP def2/J TightSCF defgrid3 RIJCOSX ENGRAD ' > $ofile
echo '! nomoprint nopop' >> $ofile
echo '%maxcore 4000' >> $ofile
echo '%pal' >> $ofile
echo "  nprocs $ncpu" >> $ofile
echo 'end' >> $ofile
echo '%scf' >> $ofile
echo '  maxiter 350' >> $ofile
echo 'end' >> $ofile
echo '%elprop' >> $ofile
echo ' dipole false' >> $ofile
echo 'end' >> $ofile
# charge and spin multiplicity
echo '* xyz 1 1' >> $ofile
cat $molfile >> $ofile
echo '*' >> $ofile
cd ..

# echo "running: orca $ofile > $ofileout"
orig=$PWD
cp scratch/orcain$1.in $TMPDIR/
cd $TMPDIR
$EBROOTORCA/orca orcain$1.in > $ofileout
cp $ofileout $orig/scratch/
cd $orig
```

The following is the template odata file for QCI-HEV runs with OPTIM package. For actual runs, the variable atom_num should be replaced with the number of atoms in the specific reaction, and the line beginning with ORCAMPI should be edited with the values of charge, multiplicity of the system and the path to the ORCA binary. The coordinates of the RS in xyz format should be appended to this file, underneath POINTS. Finally, only the

coordinates of the PS (without elements) should be added as a new file named `finish` in the same directory.

```
! Input file for QCI-HEV
DEBUG

! Stop after TS, do not calculate paths
TSSTOP

! QCI interpolation parameters -
! based on recommendations from Wales group
CONCUTABS 0.2 0.05
QCIRESET 2000
QCI 1.D-3 1.0 1.0 9.0 0.0 atom_num 0 300000 1000 400000 1.0 1.5
CHECKREP 100 1.25
! Parameter 2 sets max number of images
QCIIMAGEDENS 50.0 8
KINT 3.0
QCIADJUSTK 5 12.0 1.01 1.0 3.0
INTSPRINGACTIVE
INTFREEZE 0.02D0 5
MAXCON 10
QCICONCUT 50.0
QCICYCLES 0.0 200

! HEVF search parameters
! (1) maximum iters in each step for converging to min mode
! (2) # perp. steps when eigenvalue overlap is small
! (3) # perp. steps when eigenvalue overlap is close to 1.0
! (4) RMS grad threshold for Rayleigh-Ritz phase
BFGSTS 60 3 8 0.001
! HEVF search percentage change threshold for eigenvalue
BFGSTSPC 1.0
! Maximum number of BFGSTS steps
STEPS 70
! Dump xyz files at each step of BFGSTS
HEFPRINT
! HEVF use one-directional derivative
! (saves time, if the numbers have enough precision)
USEDIAG 3
! HEVF size for finite difference
SECDIFF 0.006
! Turn off explicit Hessian calculation
NOHESS
! Maximum number of steps in the LBFGS minimiser for pathways
BFGSSTEPS 1000
! energy difference and geometry difference tolerances for
! recognising stationary points
EDIFFTOL  1.0D-4
```

```
GEOMDIFFTOL  0.1D0
! Convergence criteria for LBFGS minimiser (initial minima and path runs)
BFGSMIN 5.0D-4
! Convergence criteria for BFGSTS (1) step size length
! - it is safe to set this large and just use the RMS gradient
! (2) RMS gradient
CONVERGE 0.05 1.0D-4
UPDATES 50 50 15 15
MAXERISE 1.0D-5 1.2D0
NEBMAXERISE 1.0D-1
! Maximum stepsize for (1) HEVF and (2) steepest descent
MAXSTEP  0.2
! Trust ratio
TRAD     0.2
MAXMAX   0.2
MINMAX 0.005
! Maximum stepsize for (1) LBFGS minimisation, (2) Rayleigh-Ritz
! ratio minimization (3) not used any more (4) DNEB minimisation
MAXBFGS 0.1 0.15 0.15 0.15

DUMPALLPATHS
! Minimum number of minimisation steps
! - to prevent premature convergence at the ts
STEPMIN 5
! Do not calculate frequencies
NOFRQS

! Double-ended TS search
! - parameter 4 sets max. iter * number of images <= set to zero
! parameter 5 sets max number of DNEB images
! parameter 7 sets the RMS gradient convergence threshold (not used here)
NEWCONNECT 1 1 50.0 0.0 8 1.0 0.005
! Spring constant and adjustment for DNEB
NEBK 0.5
ADJUSTK 20 2.0 1.02D0
! Minimise endpoints at the beginning if they are outside tolerance
REOPTIMISEENDPOINTS

ORCAMPI charge mult /path/to/orca

POINTS
```

For DNEB-HEV runs, only the parameters related to the double-ended part (`NEWCONNECT`) are different.

```
! Input for DNEB-HEV
...
! Double-ended TS search
```

```
! - parameter 4 sets max. iteration * number of images <= maxiter = 70
! parameter 5 sets max number of DNEB images
! parameter 7 sets the RMS gradient convergence threshold
NEWCONNECT 1 1 50.0 8.75 8 1.0 0.005
! Spring constant for DNEB
NEBK 1.0
! Adjust spring constant by factor 1.03 every 20 steps
! if avg. dev. of interimage distances < 2.0%
ADJUSTK 20 2.0 1.03D0
...
```

To run the OPTIM package, an `ORCA.template` file, with the ORCA input template must also be present in the same directory.

```
# ORCA template for OPTIM
! PBE0 D3BJ def2-SVP def2/J TightSCF defgrid3 RIJCOSX EnGrad pal8
! nomoprint nopop

%maxcore 4000
%elprop
 dipole false
end
```

2 AutodE implementation details

To the best of our knowledge, no publicly available, free implementations of the DHS and the i-EIP methods are available. Therefore, we have implemented them in the open-source Python package autodE. The details of the implementations and how they differ from the original publications are described below.

2.1 DHS

The original algorithm proposed by Dewar, Healy and Stewart[2] acts on the distance between the two images

$$R = \left|\mathbf{x}_R^k - \mathbf{x}_P^k\right| \tag{1}$$

where $\mathbf{x}_R^k$ and $\mathbf{x}_P^k$ are the Cartesian coordinates of the images on the reactant side and product side at iteration k, respectively. At the first iteration ($k = 0$), the images start from the given RS and PS geometries. At each step, the distance is reduced by moving the lowest energy image along the vector connecting the two images (i.e. linear interpolation), and then minimizing the energy while holding R constant. Dewar *et al.* suggest reducing the distance by a percentage. Here we use a fixed step size instead:

$$\begin{aligned} \mathbf{x}_R^{k+1} &= \mathbf{x}_R^k + s\frac{\mathbf{x}_P^k - \mathbf{x}_R^k}{\left|\mathbf{x}_P^k - \mathbf{x}_R^k\right|}, \text{ if } E_R < E_P \\ \mathbf{x}_P^{k+1} &= \mathbf{x}_P^k + s\frac{\mathbf{x}_R^k - \mathbf{x}_P^k}{\left|\mathbf{x}_R^k - \mathbf{x}_P^k\right|}, \text{ if } E_R > E_P \end{aligned} \tag{2}$$

where E_R and E_P are the electronic energies of the two images, and s is the step size. The default options set $s = 0.2\,\text{Å}$ when $R > 1.5\,\text{Å}$ and $s = 0.05\,\text{Å}$ when $R < 1.5\,\text{Å}$.

After the linear interpolation, the displaced image is then minimized by holding the distance to the other image constant. In mathematical terms, this is a minimization on the surface of a hypersphere with a center at the other image, and the radius being the distance R. The minimization is considered converged when the RMS of atomic forces reaches a threshold, set to $1 \times 10^{-3}\,E_\text{h}\,\text{Å}^{-1}$ by default. The algorithm used for this minimization is described in detail later.

When the minimization converges, the energies are calculated on the moved image. The linear interpolation step and the constrained minimization are repeated until the distance between two images is below the threshold R_tol (default 0.5 Å). At convergence, the higher-energy image from the pair is chosen as the TS guess.

Constrained minimization

After a DHS linear interpolation step, the image which has been displaced must be minimized under the constraint that the distance to the other image remains fixed. In the original publication,[2] the Davidon-Fletcher-Powell algorithm in internal coordinates was recommended.[3]

However, it cannot strictly obey the distance constraint in each step. In addition, it uses internal coordinates, which require complicated and extensive coding to set up. Therefore, we use a simple quasi-Newton minimization in Cartesian coordinates inspired by the hypersphere optimization algorithm proposed by Gonzalez and Schlegel.[4]

The minimizer iteration is denoted with index j, which starts at $j = 0$ after every linear interpolation step (index k). At step j, the coordinates of the image being minimized is $\mathbf{x}_j$ and the coordinates of the other DHS image is $\mathbf{x}^{\mathrm{L}}$ (it stays fixed throughout minimization). At $j = 0$, $R_0 = |\mathbf{x}_0 - \mathbf{x}^L|$ is the distance between the two images.

The potential energy surface around the current set of coordinates is expressed as a second-order Taylor expansion.

$$E(\mathbf{x}_j + \Delta\mathbf{x}) = E(\mathbf{x}_j) + \mathbf{g}_j^T\Delta\mathbf{x} + \frac{1}{2}(\Delta\mathbf{x})^T\mathbf{H}_j\Delta\mathbf{x} \tag{3}$$

where $\Delta\mathbf{x}$ is the displacement vector in Cartesian system, $\mathbf{x}_j$ is the vector of Cartesian coordinates of the image, while $\mathbf{g}_j$ and $\mathbf{H}_j$ are the gradient vector and the Hessian matrix at iteration j. After the DHS linear interpolation displacement step, the Hessian matrix is obtained with an update scheme from the Hessian matrix of the previous point. If no previous Hessian is available, an approximate matrix is calculated with GFN2-xTB method. For subsequent minimization steps ($j > 0$), the Hessian matrices are always updated. The update scheme uses a weighted combination of the BFGS and SR1 formula, proposed by Farkas and Schlegel.[5]

At each minimizer step, the minimum on the Taylor surface (Eqn. 3) is sought, subject to two constraints, using the `SLSQP` algorithm implemented in the Python package `scipy`. The first constraint ensures that the distance (R) between the image being minimized and the other DHS image does not change i.e. $|(\mathbf{x}_j + \Delta\mathbf{x}) - \mathbf{x}^L| = R_0$. The second constraint sets a maximum limit for the total size of the quasi-Newton step, i.e. $|\Delta\mathbf{x}| < t_{max}$. The trust radius t_{max} is set to 0.1 Å or the current DHS step size s, whichever is lowest. The new set of

coordinates are set from the minimum on Taylor surface: $\mathbf{x}_{j+1} = \mathbf{x}_j + \Delta\mathbf{x}$, and the minimizer iteration continues until RMS($\mathbf{g}_j^{\perp}$) is less than a threshold (set to $1\times10^{-3}\,E_\mathrm{h}\,\text{Å}^{-1}$ by default). The perpendicular gradient component is obtained by projecting out the component parallel to $\mathbf{x}_j - \mathbf{x}^L$ vector:

$$\mathbf{g}_j^{\perp} = \mathbf{g}_j - \frac{\mathbf{g}_j^T(\mathbf{x}_j - \mathbf{x}^L)}{|\mathbf{x}_j - \mathbf{x}^L|} \tag{4}$$

In between successive quasi-Newton steps, polynomial fitted line search is used to accelerate convergence, based on the method described by Gonzalez and Schlegel.[4]

In some steps, the `SLSQP` is unable to converge and obtain the quasi-Newton step within the trust radius t_{max}. In that case, a steepest descent step along $\mathbf{g}^{\perp}$ is taken with maximum size set to $t_{max}/2$. Note that this does not exactly obey the distance constraint ($|(\mathbf{x}_j + \Delta\mathbf{x}) - \mathbf{x}^L| = R_0$). However, in our experience, the steepest descent step is taken very rarely, so it does not degrade the performance of the optimizer.

2.2 i-EIP

Our implementation of i-EIP in autodE v1.4.3 closely follows the original method reported by Lei and co-workers.[1] The procedure begins by generating an image-pair between the given RS and PS geometries. An initial path is constructed using IDPP between the RS and PS. The number of images set to $\max\left(\lfloor R_{init}\rho_{im} - 1\rfloor, 7\right)$, where $\lfloor R_{init}\rfloor$ indicates the floor function and R_{init} is the initial Euclidean distance between RS and PS coordinates expressed in units of Ångstrom, and ρ_{im} is the approximate image density in units of Å^{-1}. By default ρ_{im} is set to $1.0\,\text{Å}^{-1}$. A minimum of seven images is always used, so that at least five intermediate images describe the path. While the original publication used LST[6] interpolation for this path, we have used IDPP since it has been shown to provide better paths.[7]

Energies are then calculated for all images along the interpolated path. A cubic spline is

fitted to the Cartesian coordinates of each image, and another spline with the energies using the normalized cumulative inter-image Euclidean distance as the parameter. If there are N image, the spline parameter t_n for the n-th image is defined as (with $t_0 = 0$ and $t_N = 1$)

$$t_n = \frac{\sum_{i=1}^{n} |\mathbf{x}_i - \mathbf{x}_{i-1}|}{\sum_{i=1}^{N} |\mathbf{x}_i - \mathbf{x}_{i-1}|} \tag{5}$$

The splines are required to satisfy $S_{\mathbf{x}}(t_n) = \mathbf{x}_n$ for Cartesian coordinates and $S_E(t_n) = E_n$ for energies. Note that for fitting Cartesian coordinates, each x, y and z coordinates of each atom are essentially treated with separate 1D cubic splines. The `CubicSpline` class from `scipy.interpolate` module is used for all fitting.

The maximum along the interpolated path is located by evaluating $S_E(t)$ within $t \in [0, 1]$, and finding all values where the derivative with respect to t is zero. The stationary point with the highest energy is selected as t_{peak}, applying the extreme value theorem. If $t_{\text{peak}} = 0$ or 1 i.e., the peak is located at the RS and PS geometries, an exception is raised and the algorithm stops. The total length of the interpolated path, l_{total} is obtained by integrating $S_{\mathbf{x}}(t)$. The image-pair is generated at $l_{\text{total}} \times 0.25$ distance away from t_{peak} on both sides.

After the image-pair is generated, energies and gradients are evaluated at the DFT level of theory. An approximate Hessian is also obtained at the GFN2-xTB level of theory. Micro-iterations are performed on a second order Taylor approximation of the PES constructed from the energy, gradient and approximate Hessian. The objective of these micro-iterations is to minimize (i) the component of RMS gradient perpendicular to the image-pair vector, (ii) the energy difference between two images, and (iii) the distance between the two images. We follow the exact procedure for micro-iterations as outlined in the original publication; therefore, we direct interested readers to ref.[1] for further details. After each set of micro-iterations the gradients are recalculated and the Hessian approximation is updated using the formula proposed by Bofill.[8]

The iterations continue until the distance between the two images is less than R_{tol}, set to 0.3 Å by default, and RMS($\mathbf{g}^{\perp}$) i.e. the gradient component perpendicular to the image-pair

vector, is less than a threshold, set to $1 \times 10^{-2}\, E_\mathrm{h}\, \mathrm{\AA}^{-1}$ by default. The original publication recommended $0.6\, a_0$ (≈ 0.31 Å) and $0.012\, E_\mathrm{h}\, a_0^{-1}$ ($\approx 2.3 \times 10^{-2}\, E_\mathrm{h}\, \mathrm{\AA}^{-1}$). We have tightened the gradient threshold to obtain better converged TS guesses.

At convergence, the mid-point between the two images, obtained in Cartesian coordinates, is taken as the TS guess. The original publication recommended checking the quality of the TS guess by obtaining the overlap of the image-pair vector with the *min-mode* of the calculated Hessian, and continuing the i-EIP iterations with tightened criteria. In addition, it also suggested a TS guess refinement procedure by choosing internal coordinates with high components in the *min-mode* eigenvector. After the refinement, another Hessian matrix is required to start the TS optimization. Due to the computational cost of calculating the Hessian matrix, these stages are skipped, and we directly start the TS optimization after initial convergence.

3 Benchmark dataset

3.1 Choice of reactions

Our dataset is mostly based on the Metal–Organic Barrier Heights, 35 reactions (MOBH35) dataset collated by Iron and Janes.[9] MOBH35 provides reactant, TS, and product geometries for 35 organometallic reactions. However, we revised the dataset by removing similar reactions. For example, we removed reactions 4, 16, 24, and 32 as they correspond to the same reaction type as reactions 3, 15, 25 and 15, respectively. We also excluded reactions 8 and 9 due to their multireference character, as identified by Martin et al.[10] This initial filtering led to a total of 29 reactions.

We further removed reactions 17-20 and 35 because the literature TS optimized at our level of theory did not yield the reported reactants and products. For reaction 26, we were unable to obtain a converged TS starting from the literature TS. This left us with 23 reactions from the original MOBH35 set. To increase the dataset size, we added seven

additional organometallic reactions identified during our literature review, resulting in a final dataset of 30 reactions (details in the main text).

3.2 Modifications to reactions

Some reactions were simplified compared to the original literature in order to reduce computational effort. In addition, for reaction 24, the literature reactant (with two H coordinated to Pt) was not a stable minimum at PBE0-D3BJ/def2-SVP level of theory, and collapsed to a modified reactant (η^2-H_2 Pt complex). Therefore, we used the η^2-H_2 reactant as our reference (see below). The modifications are as follows:

Reaction 2

Original reaction:

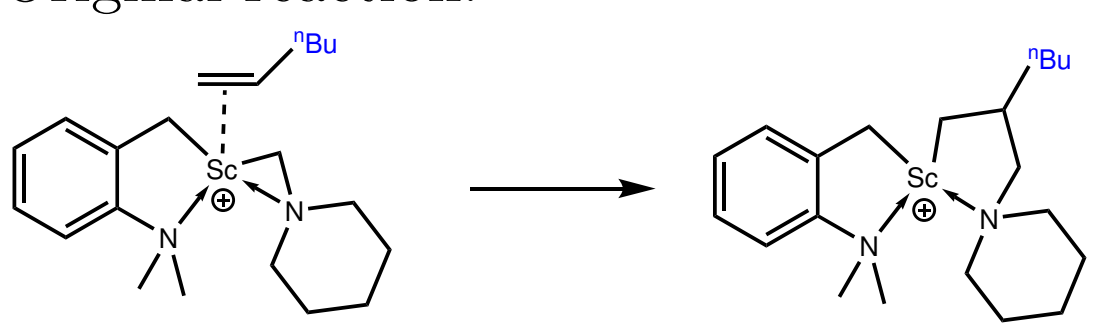

Simplified reaction:

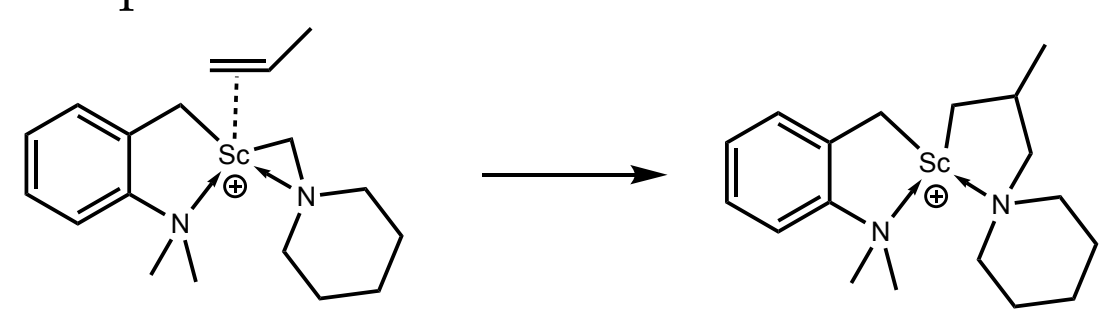

Reaction 4

Original reaction:

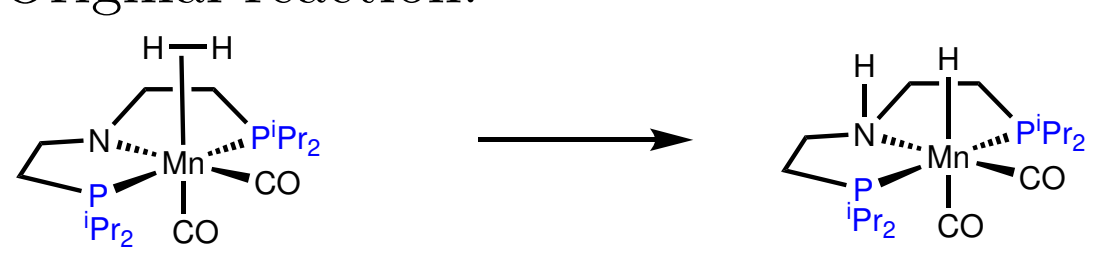

Simplified reaction:

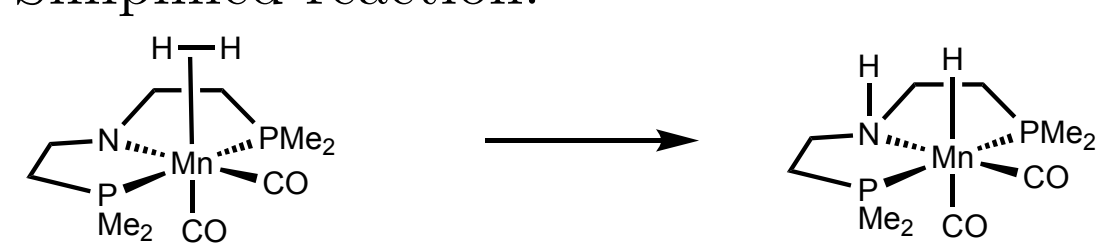

Reaction 7

Simplified reaction:

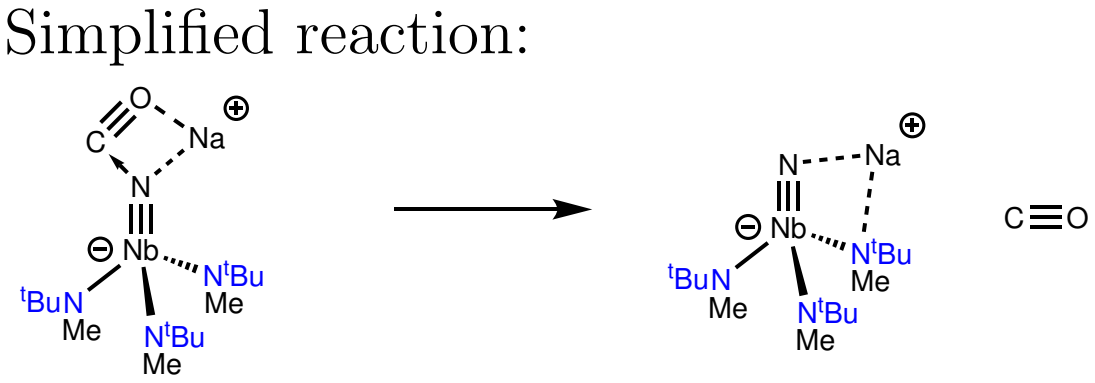

Modified reaction:

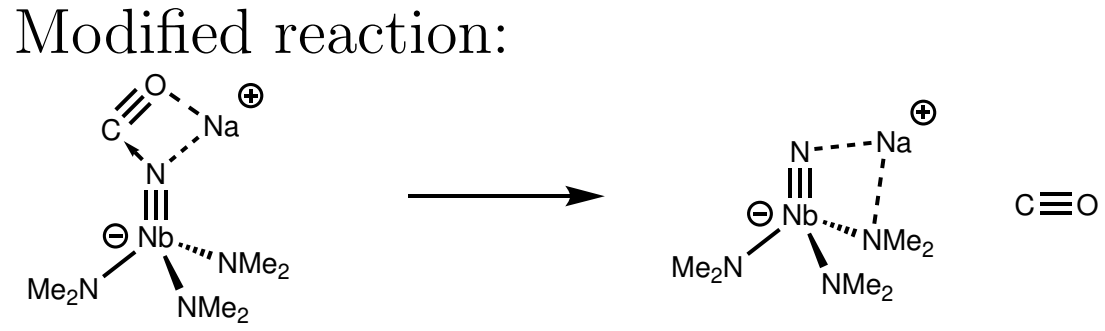

Reaction 17

Original reaction:

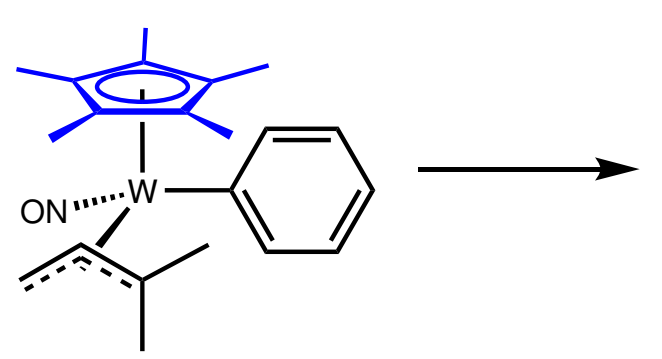

Modified reaction:

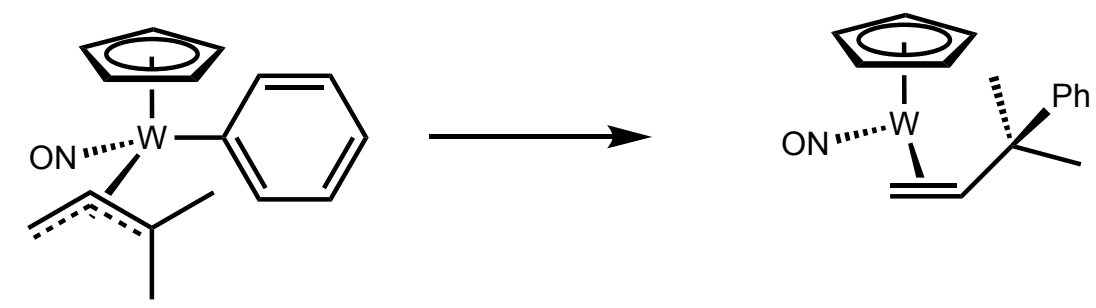

Reaction 18

Original reaction:

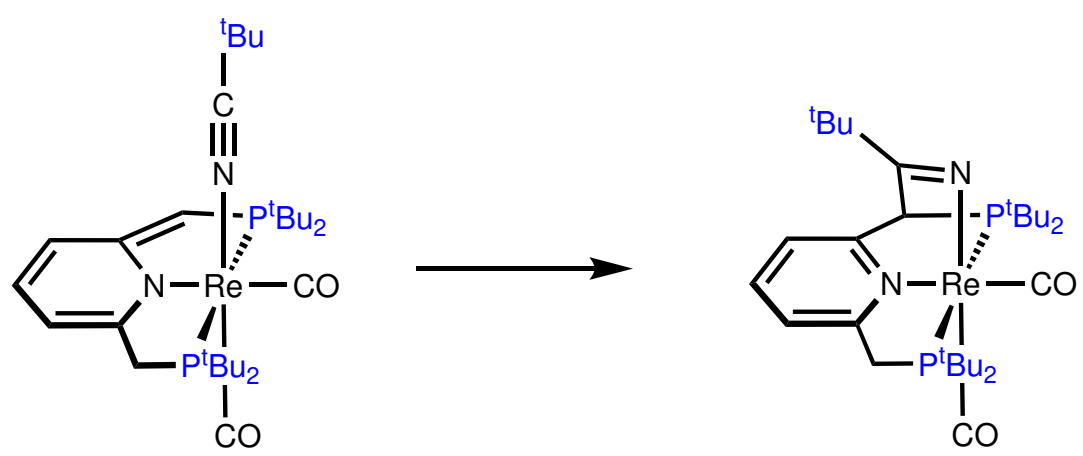

Modified reaction:

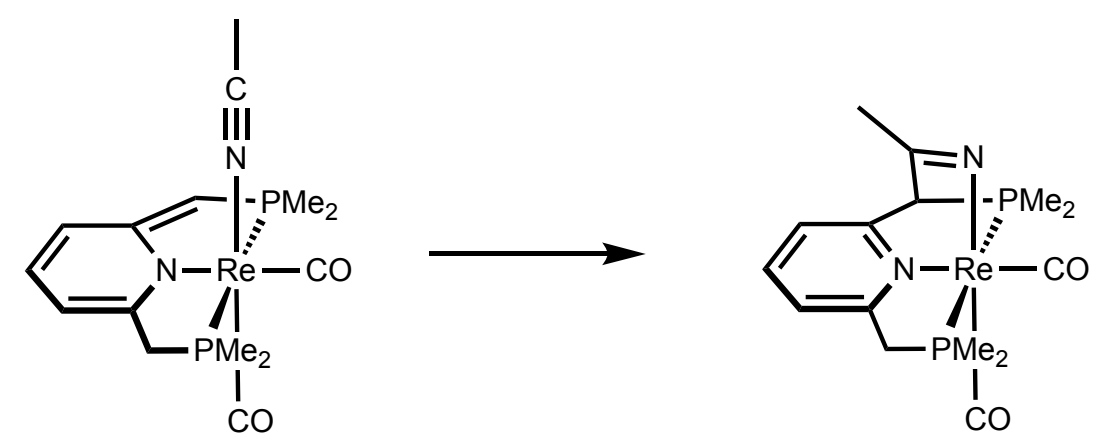

Reaction 24

Original reaction:

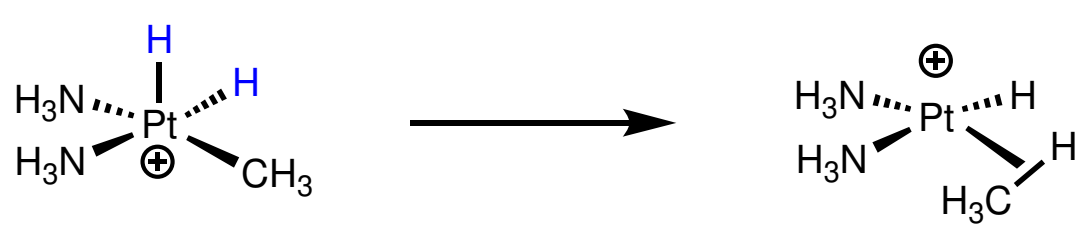

Modified reaction:

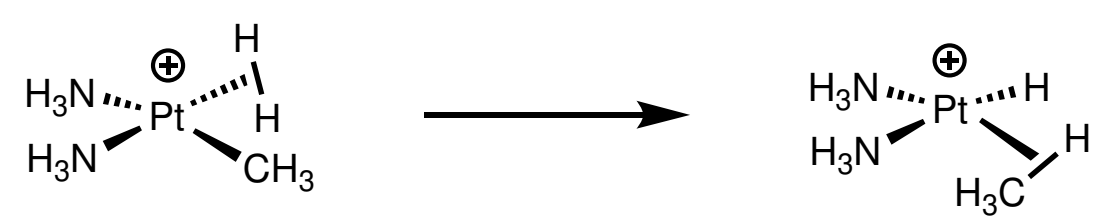

Reaction 28

Original reaction:

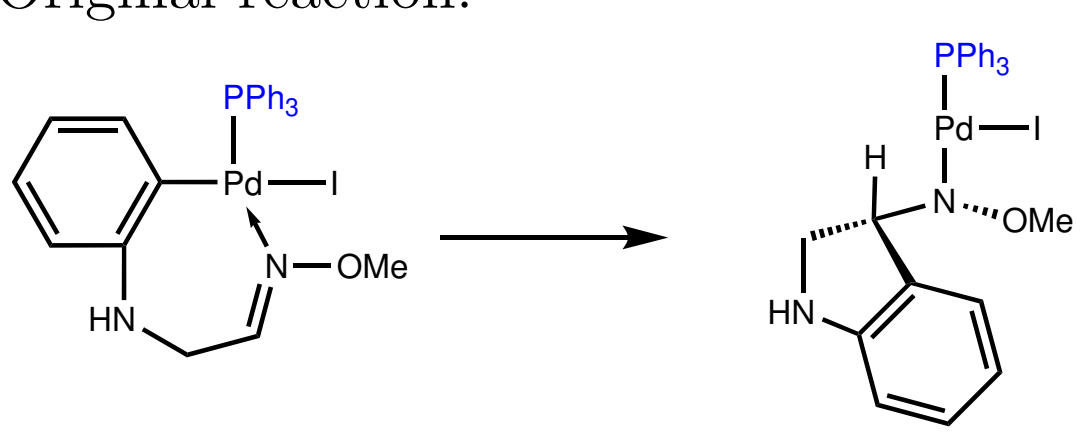

Modified reaction:

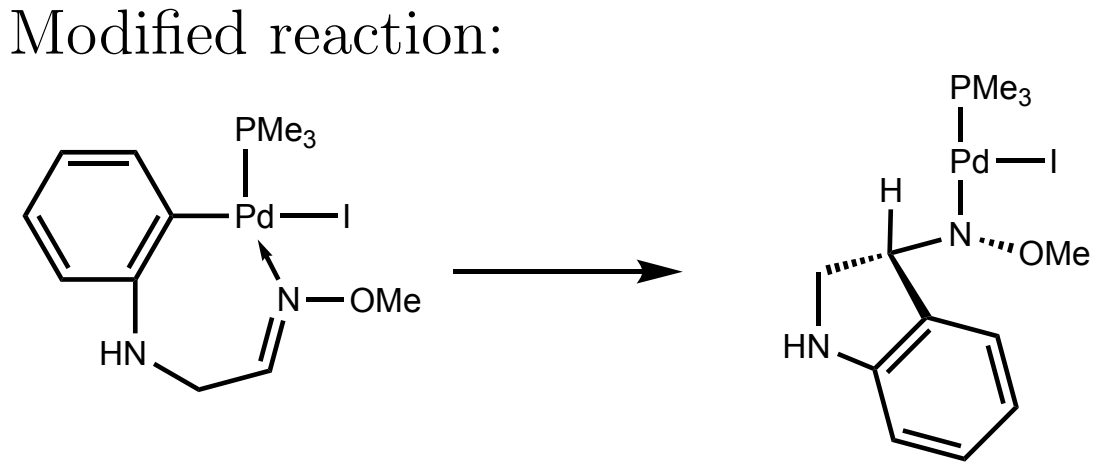

3.3 Preparation of benchmark set

The procedure for preparing the benchmark dataset is described in the main text (Section-2.3); here, we provide details about the calculations and modifications to the default protocol required for specific reactions.

(1) In the first step, *.xyz files for all TSs were extracted, or generated from Cartesian coordinates from literature. These are then considered as guess TSs for optimization at

our level of theory. In cases where a reaction was simplified, the `xyz` file was edited with Avogadro v1.2.0 to obtain the corresponding simplified guess TS.

These were re-optimized at PBE0-D3BJ/def2-SVP level with tight optimization criteria in ORCA. For TS optimization, the Hessian matrix was calculated in the first iteration and every 10 steps after that. Trust radius was set to `-0.2`, where the negative sign indicates that the trust update is turned off. The maximum number of iterations was set to 600. At the end of the optimization, frequencies (Hessian) were calculated to confirm that a saddle point was obtained.

In case of reaction 15, there was a weak secondary imaginary frequency ($\omega_i = -123.7$ and $-20.9\,\mathrm{cm}^{-1}$) after initial optimization. Therefore, we displaced the geometry along the second imaginary mode and re-optimized with very tight convergence criteria, and a trust radius of `-0.1` to obtain the final TS.

(2) Starting from the optimized TSs, IRC calculations were performed in ORCA with a maximum number of iterations set to 600 and the initial displacement from the TS corresponding to an energy difference of $1.0\,\mathrm{m}E_\mathrm{h}$. In Reactions 14, 15, 19, 21, and 24, the initial displacement was too large, leading to increases in energy. This means the obtained IRC is no longer a fully descending path and may therefore be qualitatively incorrect. Therefore, smaller step sizes were used in order to obtain a consistently downhill path: for reaction 14, initial displacement at TS was set to $0.15\,a_0$. For reactions 15, 19, 21 and 24 we set the initial displacements to 0.25, 0.15, 0.2 and $0.2\,a_0$ respectively; in addition, the RMS and maximum component of gradient convergence criteria for IRC were set to $5 \times 10^{-5}\,E_\mathrm{h}\,{a_0}^{-1}$ and $1 \times 10^{-4}\,E_\mathrm{h}\,{a_0}^{-1}$ respectively to avoid premature convergence near the TS. For reactions 15, 19, and 21, the maximum number of iterations was set to 100; for reaction 24, it was set to 150. The IRC trajectories for all reactions were visualized to check if they represent the valid reaction, especially in cases where the reaction was simplified.

(3) The IRC end-points were minimized in ORCA with tight optimizer convergence criteria. The maximum number of iterations was set to 600. For both end-points in reaction 4,

and one end-point for reactions 12 and 19, the obtained minima had imaginary frequencies, indicating they had not converged correctly. At first, the optimizations were restarted with very tight convergence criteria, which provided minima with no imaginary modes for reaction 19. For reactions 4 and 12, the geometries from the preliminary minimization were displaced along the imaginary mode and then minimized again with very tight convergence criteria, which returned valid minima with no imaginary modes.

4 Benchmark results

4.1 Automatic assignment of TS search outcomes

The procedure for automatic assignment has been described in the main text. The result of this initial assignment is shown below.

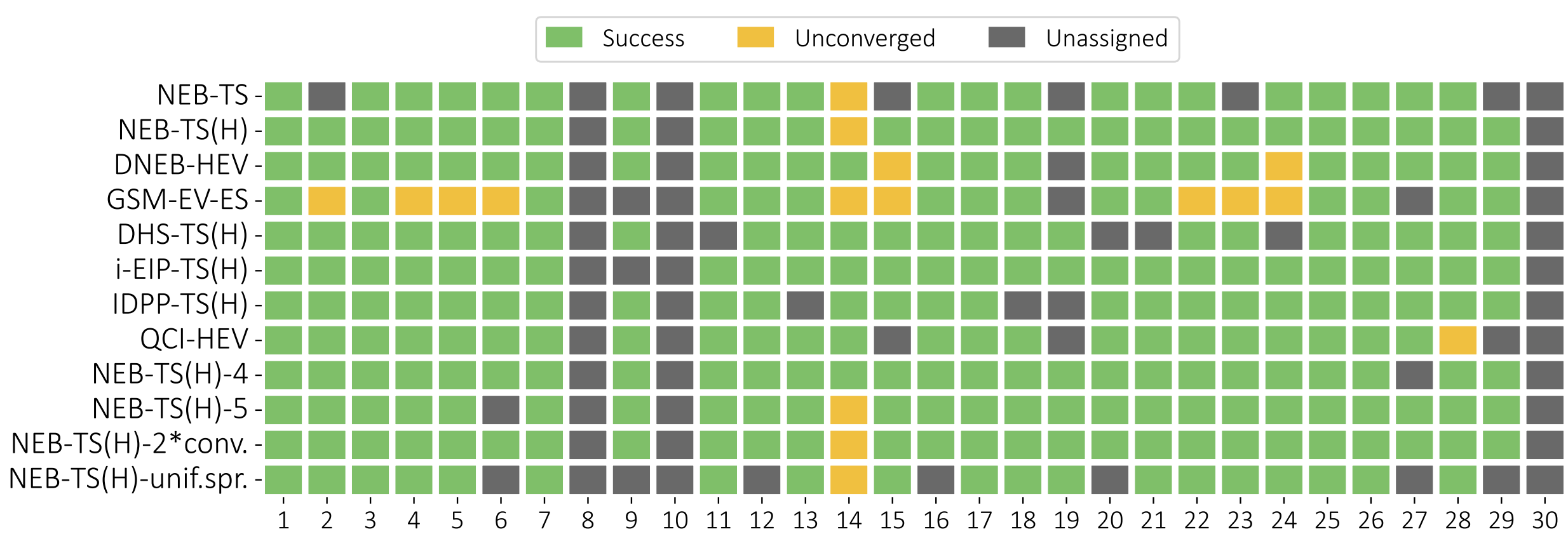

Figure S1: Heatmap showing assignments of the outcome of hierarchical double-ended TS searches, obtained from the automated protocol. The TSs are classified as success (green) or unconverged (yellow); remaining TSs are unassigned (grey).

4.2 Manual assignment of remaining TSs

The remaining geometries from double-ended methods were assigned manually. The justifications for each TS, grouped by the method, are given below.

1. **NEB-TS**

- Reaction 2 – The obtained geometry had two weak imaginary modes, $-41.99\,\mathrm{cm^{-1}}$ and $-31.03\,\mathrm{cm^{-1}}$, corresponding to molecular breathing and no bond breaking. The residual RMS gradient is high ($0.004\,95\,E_\mathrm{h}\,{a_0}^{-1}$). Attempting to optimize this to a valid saddle point fails. (Assigned as failure)

- Reaction 8 – The TS is part of a multi-step pathway; a detailed description is in the main text. (Assigned as failure, removed from data analysis)

- Reaction 10 – Running the IRC calculation confirms that the obtained TS leads to the same reactant and product. The TS is very similar in geometry to the reference TS (RMSD = 0.028 Å); however, the overlap of the first imaginary mode is 0.77, which leads to failure in assignment by the automated algorithm. The first imaginary frequency for this TS is $-683.54\,\mathrm{cm^{-1}}$ while for the ref. TS is $-1069.77\,\mathrm{cm^{-1}}$. Further discussion can be found in the main text in Section-3.2.3. (Assigned as success)

- Reaction 15 – The obtained geometry had nine imaginary modes ranging from $-340.88\,\mathrm{cm^{-1}}$ to $-51.39\,\mathrm{cm^{-1}}$. The first three imaginary modes all correspond to methyl rotations. The residual RMS gradient is high, $0.010\,89\,E_\mathrm{h}\,{a_0}^{-1}$. This is not close to any valid stationary point. (Assigned as failure)

- Reaction 19 – The geometry had two weak imaginary modes ($\omega_i = -29, -13\,\mathrm{cm^{-1}}$). When the IRC is calculated, the first step shows an energy increase in one direction, even with a small step size. We were unable to obtain a valid downward IRC path. Reoptimizing the geometry returns a saddle point with one weak imaginary mode ($-15\,\mathrm{cm^{-1}}$) where the imaginary mode corresponds to molecular "breathing". (Assigned as failure)

- Reaction 23 – The geometry had no imaginary modes and the gradient was low ($1.0\times10^{-4}\,E_\mathrm{h}\,{a_0}^{-1}$). The optimization went to a minimum (resembles the product state) instead of a TS. (Assigned as failure)

- Reaction 29 – The geometry is a valid saddle point, with an RMSD of 0.23 Å compared to the reference TS. IRC confirms that it connects reactant and product. The difference with reference TS arises from rotation in the dihedral angle between the carbene ring and the methylene (CH_2) ligand on Ru; however, they have the same electronic energy. (Assigned as success and identified as TS conformer)

- Reaction 30 – There geometry had high RMS gradient ($1.7\,E_\mathrm{h}\,{a_0}^{-1}$) and five imaginary frequencies. Visualization of the largest magnitude imaginary mode shows that it corresponds to the correct reaction coordinate, and the overlap with the reference TS imaginary mode is 0.9. Re-optimizing obtains a correct TS, confirmed by IRC. (Assigned as unconverged and identified as TS conformer)

2. **NEB-TS(H)**

- Reaction 8 – The obtained geometry had two imaginary frequencies (-308 and $-20\,\mathrm{cm}^{-1}$). Reoptimizing leads to the same TS as obtained from NEB-TS. It is a part of a multi-step pathway. Please see further discussion in the main text. (Assigned as failure, removed from data analysis)

- Reaction 10 – The TS is correct, confirmed by IRC. See reaction 10 in the NEB-TS section above and the discussion in the main text. (Assigned as success)

- Reaction 30 – The obtained TS has two imaginary frequencies (-308 and $-20\,\mathrm{cm}^{-1}$) i.e. it is not fully converged. Reoptimizing leads to a saddle point with a single imaginary mode ($-309\,\mathrm{cm}^{-1}$) and is confirmed to be correct by IRC. (Assigned as unconverged and identified as TS conformer)

3. **DNEB-HEV**

- Reaction 8 – The geometry has a weak imaginary frequency ($-28\,\mathrm{cm}^{-1}$). Running IRC shows energy rising in one direction, even with a small step size. Visualizing the imaginary mode suggests that it is a saddle point associated with molecular

"breathing". (Assigned as failure, removed from data analysis)

- Reaction 10 – The TS has been confirmed to be correct by an IRC calculation. See reaction 10 in the NEB-TS section above and the discussion in the main text. (Assigned as success)

- Reaction 19 – The geometry shows two weak imaginary frequencies (-28 and $-19\,\mathrm{cm}^{-1}$). Running IRC shows energy rising in one direction, even with a small step size. Attempting to reoptimize leads to a saddle point associated with molecular "breathing". (Assigned as failure)

- Reaction 30 – The geometry has a weak imaginary mode ($-7\,\mathrm{cm}^{-1}$). Visualization of the normal mode shows that it is associated with a "breathing" mode. The geometry also resembles the RS state. (Assigned as failure)

4. **GSM-EV-ES**

- Reaction 8 – The geometry has one weak imaginary frequency ($-86\,\mathrm{cm}^{-1}$). Running IRC shows that it is associated with a ring sliding of the fluorobenzene on the Nb metal. (Assigned as failure, removed from data analysis)

- Reaction 9 – The geometry had two weak imaginary frequencies (-79 and $-10\,\mathrm{cm}^{-1}$). IRC calculation following the first imaginary frequency led to energy rises, even with small step sizes. Reoptimizing and then running IRC also showed energy rises in the path. The imaginary frequencies seem to be associated with "breathing" modes. (Assigned as failure)

- Reaction 10 – The overlap of the imaginary mode with reference TS is 0.77; however, IRC confirmed it is the correct TS. Please see reaction 10 in the NEB-TS section above, and the discussion in the main text (Section-3.2.3) for further details. (Assigned as success)

- Reaction 19 – The obtained geometry had three imaginary frequencies, with a

high overlap of the first imaginary mode with reference TS (0.78). Reoptimizing led to the correct TS, confirmed by IRC. (Assigned as unconverged)

- Reaction 27 – The geometry has high gradient ($2.4 \times 10^{-3}\,E_\mathrm{h}\,{a_0}^{-1}$) and one weak imaginary frequency ($-45\,\mathrm{cm}^{-1}$). However, it is close in energy ($\Delta E = 0.5\,\mathrm{kcal\,mol}^{-1}$) to the reference TS. Reoptimizing obtains the correct TS. (Assigned as unconverged)
- Reaction 30 – The obtained geometry had four imaginary frequencies. Visualization of the normal modes showed that the strongest imaginary frequency corresponded to the correct reaction coordinate. Reoptimization returns a conformer of the reference TS, confirmed by IRC. (Assigned as unconverged, identified as TS conformer)

5. **DHS-TS(H)**

- Reaction 8 – The geometry had a single imaginary frequency ($-263\,\mathrm{cm}^{-1}$) and is identical to the NEB-TS geometry (RMSD = 0.01, $\Delta E < 0.1\,\mathrm{kcal\,mol}^{-1}$). This is part of the multi-step pathway. Please see the discussion in the main text. (Assigned as failure, removed from data analysis)
- Reaction 10 – TS is correct, as confirmed by IRC. See reaction 10 in the NEB-TS section above and the discussion in the main text. (Assigned as success)
- Reaction 11 – Obtained TS had one imaginary mode ($-181.3\,\mathrm{cm}^{-1}$), corresponding to the rotation of an N-methyl group. (Assigned as failure)
- Reaction 20 – The geometry is a valid saddle point. IRC confirms that the TS connects the chemically correct reactant and product. Compared to the reference TS, the phenyl ligand lies in a different orientation, which leads to high RMSD, 0.28 Å. (Assigned as success and identified as TS conformer)
- Reaction 21 – The geometry is a valid saddle point. IRC confirms that it connects the correct RS and PS. The difference with reference TS arises from a change in

the relative position of water molecules acting as proton shuttles. (Assigned as success and identified as TS conformer)

- Reaction 24 – Obtained TS had one imaginary mode ($-844.3\,\text{cm}^{-1}$). IRC confirms that the TS connects the reactant and product. Compared to the reference TS, the obtained TS has a differing orientation of the forming C−H bond, and is $0.8\,\text{kcal mol}^{-1}$ higher in energy. (Assigned as success and identified as TS conformer)
- Reaction 30 – Obtained TS had one imaginary frequency ($-310\,\text{cm}^{-1}$) and IRC confirms it connects the correct RS and PS by IRC calculation. (Assigned as success and identified as TS conformer)

6. **i-EIP-TS(H)**

- Reaction 8 – The geometry had a single imaginary frequency ($-263\,\text{cm}^{-1}$), and is identical to the NEB-TS geometry (RMSD = 0.01, $\Delta E < 0.1\,\text{kcal mol}^{-1}$). This is part of the multi-step pathway. Please see discussion in the main text. (Assigned as failure, removed from data analysis)
- Reaction 9 – The geometry had low gradient and one imaginary frequency ($-69\,\text{cm}^{-1}$). The IRC path showed that energy rises even with a small step size, i.e., we could not obtain a steepest descent path. The imaginary frequency seems to be associated with a "breathing" mode. (Assigned as failure)
- Reaction 10 – The TS is correct, as confirmed by IRC. See reaction 10 in the NEB-TS section above and the discussion in the main text. (Assigned as success)
- Reaction 30 – IRC confirms that the obtained TS connects the correct RS and PS. (Assigned as success and identified as TS conformer)

7. **IDPP-TS(H)**

- Reaction 8 – The geometry had a high gradient ($3.2 \times 10^{-3}\, E_\mathrm{h}\, {a_0}^{-1}$) and six imaginary frequencies, with the first mode being $-807\,\mathrm{cm}^{-1}$. Visualizing the geometry shows that the F^- had detached from the metal, and the first imaginary mode corresponds to the abstraction of a methyl H by F^-. (Assigned as failure)
- Reaction 10 – The obtained TS is correct, confirmed by IRC. See reaction 10 in the NEB-TS section above and the discussion in the main text. (Assigned as success)
- Reaction 13 – The obtained geometry has a low gradient and single imaginary frequency ($-544\,\mathrm{cm}^{-1}$). IRC calculation confirms that the obtained TS leads to the same reactant and product. However, the overlap of the first imaginary mode with the reference TS is 0.81, and the RMSD is 0.26 Å. The difference with reference TS arises mainly from a different orientation of the −NOMe group. (Assigned as success and identified as TS conformer)
- Reaction 18 – The obtained geometry had two weak imaginary frequencies (-66 and $-32\,\mathrm{cm}^{-1}$). The geometry resembles RS, and both imaginary frequencies correspond to molecular "breathing" and methane rotation, respectively. (Assigned as failure)
- Reaction 19 – The geometry had no imaginary modes. The optimization converged to a minimum (resembling the product state) instead of the TS. (Assigned as failure)
- Reaction 30 – The obtained TS has been confirmed to connect the correct RS and PS through an IRC calculation. (Assigned as success and identified as TS conformer)

8. **QCI-HEV**

- Reaction 8 – The obtained TS has a single imaginary frequency ($-249\,\mathrm{cm}^{-1}$). IRC shows that it is, in fact, a conformer of the NEB-TS geometry, i.e. a

part of the multi-step pathway. It is slightly higher in electronic energy (ΔE = 4.4 kcal mol^{-1}) than NEB-TS. (Assigned as failure, removed from analysis)

- Reaction 10 – The TS is correct, confirmed by IRC. See reaction 10 in the NEB-TS section above and the discussion in the main text. (Assigned as success)
- Reaction 15 – A valid TS was found, which connects the correct reactant and product, as confirmed by IRC. The difference with the reference TS is due to a slightly different position of an N−Me group attached to the metal. The overlap of first imaginary mode is 0.9 Å which leads to not being assigned by the automated algorithm. The RMSD with ref. TS is 0.12 Å (Assigned as success and identified as TS conformer)
- Reaction 19 – The obtained geometry had two weak imaginary frequencies (−29 and −17 cm^{-1}). Running the IRC calculation by following the first imaginary mode downward leads to energy increases along the trajectory, even with a small step size. Reoptimizing the geometry leads to a "breathing" mode. (Assigned as failure)
- Reaction 29 – A valid saddle point was found, which connects the chemically correct reactant and product, as confirmed by IRC. The difference with reference TS is due to the orientation of the carbene ring compared to the methylene (CH_2) ligand, which leads to an RMSD of 0.32 Å. (Assigned as success and identified as TS conformer)
- Reaction 30 – A valid TS was obtained, confirmed to connect the correct RS and PS through an IRC calculation. (Assigned as success and identified as TS conformer)

9. **NEB-TS(H) 4 images**

- Reaction 8 – The obtained geometry has a single imaginary mode (−23 cm^{-1}). Visualization shows that this imaginary mode corresponds to molecular "breath-

ing" and the geometry resembles the reactant state. (Assigned as failure, removed from analysis)

- Reaction 10 – The TS is correct, confirmed by IRC. See reaction 10 in the NEB-TS section above and the discussion in the main text. (Assigned as success)
- Reaction-27 – There are three imaginary modes -228, -70 and $-39\,\text{cm}^{-1}$. The strongest imaginary mode corresponds to the rotation of the $-PH_3$ group. (Assigned as failure)
- Reaction-30 – The geometry shows three imaginary modes -37, -18 and $-6\,\text{cm}^{-1}$. The strongest imaginary mode corresponds to molecular "breathing" and the geometry resembles the reactant state. (Assigned as failure)

10. **NEB-TS(H) 5 images**

- Reaction 6 – The CI-NEB part of the calculation did not converge in 500 iterations. (Assigned as failure)
- Reaction 8 – The TS is part of the multi-step pathway. (Removed from data analysis)
- Reaction 10 - The TS is correct, as confirmed by IRC. See reaction 10 in the NEB-TS section above and the discussion in the main text. (Assigned as success)
- Reaction 30 – The obtained TS had two imaginary modes (-312.1 and $-8.2\,\text{cm}^{-1}$). Visualization of normal mode showed that the strongest imaginary mode represents the correct reaction coordinate. Reoptimization returns a conformer of the reference TS, confirmed by IRC. (Assigned as unconverged and identified as TS conformer)

11. **NEB-TS(H) coarse convergence**

- Reaction 8 – The TS is part of the multi-step pathway. (Removed from data analysis)

- Reaction 10 – The TS is correct, as confirmed by IRC. See reaction 10 in NEB-TS section and the discussion in the main text. (Assigned as success)
- Reaction 30 – The obtained TS is a conformer of the reference TS, confirmed by IRC. (Assigned as success and identified as TS conformer)

12. **NEB-TS(H) uniform springs**

- Reaction 6 – The CI-NEB calculation failed to converge. (Assigned as failure)
- Reaction 8 – The fluorobenzene seems to have dissociated from the Nb catalyst. (Assigned as failure, removed from analysis)
- Reaction 9 – The geometry had a single imaginary mode $-14.6\,\mathrm{cm}^{-1}$. Visualization shows that this mode corresponds to a rotation of the $-PMe_3$ group. (Assigned as failure)
- Reaction 10 – The TS is correct, as confirmed by IRC. See reaction 10 in NEB-TS section and the discussion in the main text. (Assigned as success)
- Reaction 12 – The CI-NEB calculation failed to converge. (Assigned as failure)
- Reaction 16 – The CI-NEB calculation failed to converge. (Assigned as failure)
- Reaction 20 – The obtained geometry had no imaginary modes, and is not a TS. (Assigned as failure)
- Reaction 27 – The geometry had a single imaginary mode $-212.0\,\mathrm{cm}^{-1}$. Visualization shows that this mode corresponds to the rotation of the $-PH_3$ group, and the geometry resembles the product. (Assigned as failure)
- Reaction 29 – The geometry shows a single imaginary mode $-32.3\,\mathrm{cm}^{-1}$. Visualization shows that this mode corresponds to molecular "breathing". (Assigned as failure)
- Reaction 30 – The final geometry had three imaginary modes -39.9, -26.3 and

$-10.4\,\mathrm{cm}^{-1}$. These modes correspond to $-PH_3$ rotation and molecular "breathing". (Assigned as failure)

After manual assignment, all geometries obtained from hierarchical double-ended TS search are classified into (i) success, (ii) unconverged or (iii) failure. The results are shown in a heatmap below.

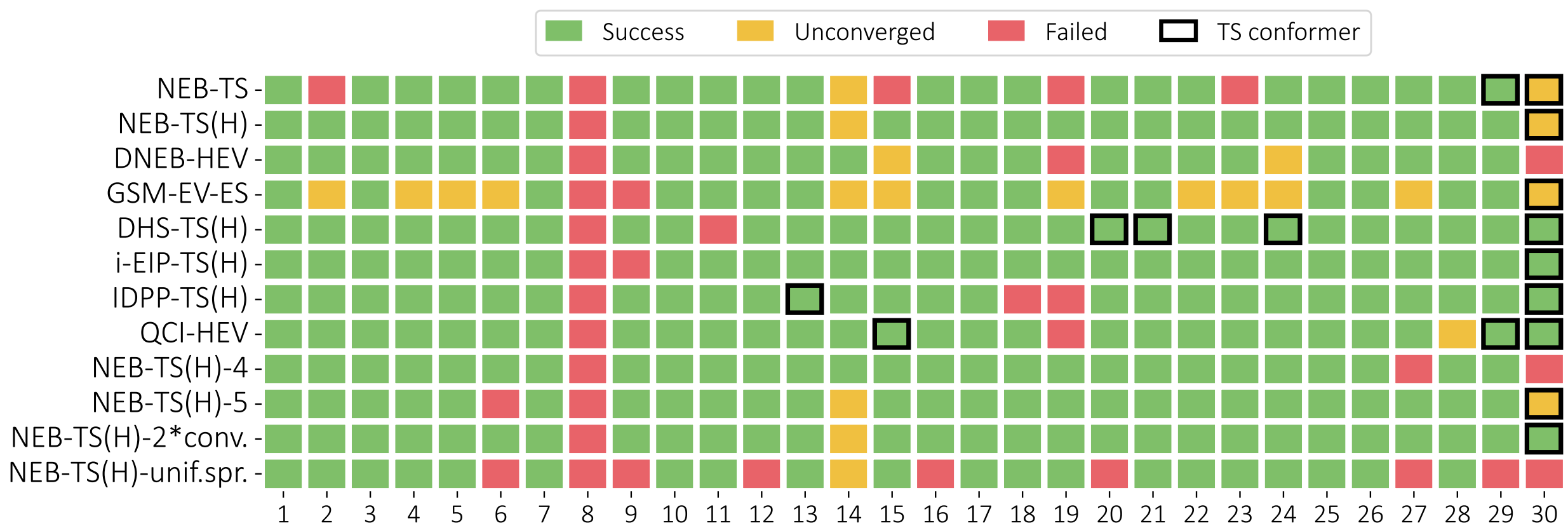

Figure S2: Heatmap showing final assignments after manual analysis. The TSs are classified as success (green), unconverged (yellow) and failure (red). TS conformers are indicated with a dark box.

4.3 Benchmark TS data

4.3.1 TS identification and comparison with reference

The data on the final TS geometries obtained from all hierarchical double-ended TS search methods are tabulated here. For each method, we note the root-mean squared gradient (RMS(g)), imaginary frequencies (ω_i), root-mean squared deviation from the reference TS (RMSD), the overlap of the first imaginary mode with the same for the reference TS (Overlap) and the difference in energy with the reference TS ($\Delta E = E - E_{\mathrm{ref}}$). The geometry is a strict TS if RMS(g) $< 1 \times 10^{-3}\, E_\mathrm{h}\, {a_0}^{-1}$ and there is only a single ω_i. The geometry matches the reference TS if RMSD < 0.15 Å and overlap of first imaginary mode > 0.9. The outcomes of these checks are also shown in separate columns with 'Y' and 'N' indicating yes or no.

Table S2: Data for TS geometries obtained with **NEB-TS** on benchmark set

ID	RMS(g) ($E_\mathrm{h}\,a_0{}^{-1}$)	ω_i (cm^{-1})	strict TS?	RMSD (Å)	Overlap	matches ref. TS?	ΔE (kcal/mol)
1	3.47E-05	-1185.76	Y	0.01	1.00	Y	0.0
2	4.96E-03	-41.99, -31.03	N	0.46	0.00	N	3.0
3	5.71E-05	-147.05	Y	0.02	1.00	Y	0.0
4	6.07E-05	-769.6	Y	0.00	1.00	Y	0.0
5	2.99E-05	-1198.1	Y	0.00	1.00	Y	-0.0
6	4.26E-05	-667.18	Y	0.00	1.00	Y	0.0
7	5.20E-05	-261.1	Y	0.00	1.00	Y	0.0
8	3.44E-05	-263.22	Y	1.22	0.50	N	-27.7
9	6.53E-05	-657.11	Y	0.03	1.00	Y	0.0
10	2.98E-05	-683.54	Y	0.03	0.77	N	-1.0
11	4.66E-05	-1205.04	Y	0.00	1.00	Y	0.0
12	5.73E-05	-488.21	Y	0.00	1.00	Y	0.0
13	2.45E-05	-232.9	Y	0.01	1.00	Y	0.0
14	4.97E-05	-420.86, -17.3	N	0.02	1.00	Y	0.0
15	1.09E-02	-340.88, -327.52, -295.73, -288.16, -219.24, -210.11, -178.01, -76.1, -51.39	N	0.40	0.30	N	59.6
16	2.29E-05	-391.28	Y	0.00	1.00	Y	0.0
17	3.60E-05	-285.03	Y	0.00	1.00	Y	0.0
18	1.89E-05	-262.82	Y	0.00	1.00	Y	0.0
19	2.96E-05	-29.17, -13.01	N	0.22	0.31	N	1.0
20	5.20E-05	-1477.18	Y	0.00	1.00	Y	0.0
21	5.19E-05	-305.05	Y	0.01	1.00	Y	0.0
22	2.03E-05	-980.3	Y	0.00	1.00	Y	0.0
23	1.01E-04	–	N	0.15	0.00	–	-10.7
24	1.82E-05	-333.76	Y	0.00	1.00	Y	-0.0
25	4.90E-05	-653.81	Y	0.00	1.00	Y	0.0
26	2.52E-05	-272.82	Y	0.01	1.00	Y	0.0
27	1.14E-04	-906.74	Y	0.01	1.00	Y	0.0
28	7.89E-05	-294.02	Y	0.01	1.00	Y	0.0
29	4.71E-05	-207.49	Y	0.32	0.89	N	-0.0
30	1.72E-02	-340.72, -101.33, -45.81, -42.49, -22.3	N	0.82	0.90	N	54.4

Table S3: Data for TS geometries obtained with **NEB-TS(H)** on benchmark set

ID	RMS(g) ($E_\mathrm{h}\,a_0{}^{-1}$)	ω_i (cm^{-1})	strict TS?	RMSD (Å)	Overlap	matches ref. TS?	ΔE (kcal/mol)
1	3.81E-06	-1183.54	Y	0.00	1.00	Y	0.0
2	1.23E-05	-201.3	Y	0.00	1.00	Y	-0.0
3	2.06E-05	-148.07	Y	0.00	1.00	Y	-0.0
4	1.01E-05	-770.33	Y	0.00	1.00	Y	0.0
5	5.38E-06	-1197.66	Y	0.00	1.00	Y	-0.0
6	1.18E-05	-664.92	Y	0.00	1.00	Y	-0.0
7	1.33E-05	-259.73	Y	0.00	1.00	Y	0.0
8	1.07E-04	-263.75, -51.84	N	1.22	0.53	N	-27.4
9	7.92E-06	-641.34	Y	0.00	1.00	Y	0.0
10	3.22E-06	-684.02	Y	0.03	0.77	N	-1.0
11	7.40E-06	-1205.7	Y	0.00	1.00	Y	0.0
12	3.50E-05	-487.69	Y	0.00	1.00	Y	-0.0
13	5.57E-06	-225.87	Y	0.00	1.00	Y	-0.0
14	1.26E-05	-443.13, -6.28	N	0.00	1.00	Y	0.0
15	5.58E-06	-111.42	Y	0.00	1.00	Y	-0.0
16	4.96E-06	-391.58	Y	0.00	1.00	Y	0.0
17	5.08E-06	-285.1	Y	0.00	1.00	Y	-0.0
18	5.49E-06	-262.88	Y	0.00	1.00	Y	0.0
19	1.45E-05	-189.24	Y	0.00	1.00	Y	-0.0
20	6.45E-06	-1478.68	Y	0.00	1.00	Y	-0.0
21	5.74E-06	-297.14	Y	0.00	1.00	Y	0.0
22	4.26E-06	-981.45	Y	0.00	1.00	Y	-0.0
23	6.51E-06	-703.74	Y	0.00	1.00	Y	0.0
24	2.35E-05	-333.49	Y	0.00	1.00	Y	-0.0
25	9.29E-06	-654.72	Y	0.00	1.00	Y	-0.0
26	2.57E-05	-272.74	Y	0.00	1.00	Y	0.0
27	3.43E-05	-925.37	Y	0.00	1.00	Y	0.0
28	6.49E-05	-293.72	Y	0.01	1.00	Y	0.0
29	2.88E-05	-239.76	Y	0.02	1.00	Y	0.0
30	1.56E-04	-308.44, -20.05	N	0.93	0.75	N	0.3

Table S4: Data for TS geometries obtained with **DNEB-HEV** on benchmark set

ID	RMS(g) ($E_\mathrm{h}\, a_0{}^{-1}$)	ω_i (cm^{-1})	strict TS?	RMSD (Å)	Overlap	matches ref. TS?	ΔE (kcal/mol)
1	1.37E-04	-1185.82	Y	0.01	1.00	Y	0.0
2	2.89E-04	-200.62	Y	0.02	1.00	Y	0.0
3	8.64E-05	-157.93	Y	0.01	0.99	Y	0.0
4	9.57E-05	-769.85	Y	0.01	1.00	Y	0.0
5	8.57E-05	-1192.91	Y	0.01	1.00	Y	0.0
6	9.21E-05	-665.08	Y	0.01	1.00	Y	0.0
7	1.06E-04	-259.97	Y	0.01	1.00	Y	0.0
8	4.91E-05	-28.3	Y	0.81	0.00	N	-31.8
9	8.38E-05	-604.18	Y	0.14	1.00	Y	0.5
10	7.99E-05	-679.76	Y	0.03	0.77	N	-1.0
11	7.58E-05	-1206.73	Y	0.00	1.00	Y	0.0
12	1.94E-04	-488.65	Y	0.00	1.00	Y	0.0
13	6.00E-05	-225.62	Y	0.02	1.00	Y	0.0
14	1.13E-04	-459.62	Y	0.02	1.00	Y	0.0
15	2.25E-04	-114.24, -20.96	N	0.05	0.98	Y	0.0
16	6.51E-05	-391.69	Y	0.01	1.00	Y	0.0
17	9.21E-05	-287.75	Y	0.02	1.00	Y	0.0
18	6.09E-05	-263.16	Y	0.01	1.00	Y	0.0
19	4.67E-05	-27.79, -18.98	N	0.23	0.46	N	1.0
20	1.14E-04	-1283.1	Y	0.04	0.99	Y	0.1
21	8.73E-05	-304.57	Y	0.02	1.00	Y	0.0
22	2.17E-04	-986.17	Y	0.03	1.00	Y	0.1
23	8.20E-05	-703.16	Y	0.01	1.00	Y	0.0
24	5.97E-05	-361.61, -72.91	N	0.01	1.00	Y	0.2
25	6.55E-05	-660.41	Y	0.01	1.00	Y	0.0
26	6.60E-05	-272.3	Y	0.02	1.00	Y	0.0
27	7.41E-05	-921.94	Y	0.01	1.00	Y	0.0
28	6.62E-05	-294.04	Y	0.10	1.00	Y	0.2
29	7.47E-05	-242.18	Y	0.01	1.00	Y	0.0
30	4.12E-05	-7.38	Y	0.72	0.11	N	-15.7

Table S5: Data for TS geometries obtained with **GSM-EV-ES** on benchmark set

ID	RMS(g) ($E_\mathrm{h}\,a_0{}^{-1}$)	ω_i (cm^{-1})	strict TS?	RMSD (Å)	Overlap	matches ref. TS?	ΔE (kcal/mol)
1	1.58E-04	-1183.55	Y	0.02	1.00	Y	0.1
2	1.00E-03	-172.76	N	0.09	1.00	Y	0.7
3	4.78E-04	-165.26	Y	0.04	1.00	Y	0.1
4	1.04E-03	-786.08, -188.04, -27.98	N	0.09	0.98	Y	5.8
5	5.82E-04	-1212.65	N	0.03	1.00	Y	0.6
6	1.21E-03	-808.91	N	0.05	0.99	Y	1.4
7	9.09E-05	-258.69	Y	0.02	1.00	Y	0.0
8	7.85E-05	-86.43	Y	0.92	0.15	N	-24.0
9	6.80E-04	-79.31, -9.68	N	0.11	0.36	N	5.3
10	1.77E-04	-682.21	Y	0.03	0.77	N	-1.0
11	1.75E-05	-1206.91	Y	0.00	1.00	Y	0.0
12	2.84E-04	-491.43	Y	0.03	0.99	Y	0.1
13	9.31E-05	-244.02	Y	0.03	1.00	Y	0.1
14	7.49E-04	-246.3	N	0.09	0.90	Y	0.5
15	1.96E-05	-113.87, -23.17	N	0.04	0.99	Y	0.0
16	4.42E-04	-389.94	Y	0.03	1.00	Y	0.2
17	3.85E-05	-285.47	Y	0.02	1.00	Y	0.0
18	1.97E-04	-262.35	Y	0.01	1.00	Y	0.1
19	9.60E-04	-145.87, -34.02, -21.12	N	0.20	0.78	N	3.1
20	5.03E-05	-1489.74	Y	0.04	1.00	Y	0.1
21	1.89E-04	-453.83	Y	0.02	0.97	Y	0.2
22	6.14E-04	-966.5	N	0.02	1.00	Y	0.2
23	7.72E-04	-439.42, -13.7	N	0.15	0.96	Y	3.8
24	6.52E-05	-345.38, -23.21	N	0.01	1.00	Y	0.1
25	7.79E-05	-679.82	Y	0.02	1.00	Y	0.1
26	4.10E-05	-273.17	Y	0.01	1.00	Y	0.0
27	2.42E-03	-45.32	N	0.23	0.10	N	0.5
28	4.51E-05	-295.82	Y	0.03	1.00	Y	0.0
29	3.83E-05	-247.33	Y	0.01	1.00	Y	0.0
30	7.35E-04	-359.07, -52.79, -32.62, -4.33	N	0.69	0.60	N	8.3

Table S6: Data for TS geometries obtained with **DHS-TS(H)** on benchmark set

ID	RMS(g) ($E_\mathrm{h}\,{a_0}^{-1}$)	ω_i (cm^{-1})	strict TS?	RMSD (Å)	Overlap	matches ref. TS?	ΔE (kcal/mol)
1	5.73E-06	-1183.52	Y	0.00	1.00	Y	0.0
2	1.64E-05	-201.35	Y	0.00	1.00	Y	0.0
3	2.67E-05	-145.28	Y	0.00	1.00	Y	0.0
4	8.05E-06	-770.04	Y	0.00	1.00	Y	0.0
5	1.61E-05	-1196.29	Y	0.00	1.00	Y	-0.0
6	1.22E-05	-664.25	Y	0.00	1.00	Y	0.0
7	1.26E-05	-259.59	Y	0.00	1.00	Y	-0.0
8	1.42E-05	-262.65	Y	1.22	0.49	N	-27.7
9	1.18E-05	-642.32	Y	0.00	1.00	Y	-0.0
10	1.74E-05	-684.13	Y	0.03	0.77	N	-1.0
11	1.52E-05	-181.31	Y	0.14	0.02	N	-4.2
12	1.49E-05	-487.74	Y	0.00	0.97	Y	-0.0
13	5.11E-06	-225.1	Y	0.00	1.00	Y	-0.0
14	1.26E-05	-446.88	Y	0.00	1.00	Y	-0.0
15	4.88E-06	-111.42	Y	0.09	0.95	Y	0.0
16	4.41E-06	-391.44	Y	0.00	1.00	Y	-0.0
17	7.08E-06	-285.17	Y	0.00	1.00	Y	0.0
18	8.99E-06	-262.88	Y	0.00	1.00	Y	0.0
19	1.47E-05	-193.98	Y	0.00	1.00	Y	0.0
20	5.48E-06	-1338.29	Y	0.28	0.98	N	-0.5
21	1.32E-05	-992.98	Y	0.19	0.43	N	-0.2
22	3.58E-06	-981.37	Y	0.00	1.00	Y	-0.0
23	3.18E-06	-703.99	Y	0.00	1.00	Y	-0.0
24	7.65E-06	-844.32	Y	0.33	0.72	N	0.9
25	2.18E-05	-653.91	Y	0.00	1.00	Y	-0.0
26	7.67E-06	-272.87	Y	0.00	1.00	Y	0.0
27	1.15E-05	-930.23	Y	0.00	1.00	Y	0.0
28	1.90E-04	-292.78	Y	0.01	1.00	Y	0.0
29	1.01E-05	-241.45	Y	0.00	1.00	Y	-0.0
30	1.41E-05	-309.89	Y	0.76	0.76	N	0.0

Table S7: Data for TS geometries obtained with **i-EIP-TS(H)** on benchmark set

ID	RMS(g) ($E_h\, a_0{}^{-1}$)	ω_i (cm^{-1})	strict TS?	RMSD (Å)	Overlap	matches ref. TS?	ΔE (kcal/mol)
1	3.76E-06	-1183.55	Y	0.00	1.00	Y	0.0
2	1.59E-05	-201.42	Y	0.00	1.00	Y	0.0
3	2.97E-05	-144.7	Y	0.00	1.00	Y	0.0
4	8.28E-06	-770.3	Y	0.00	1.00	Y	-0.0
5	6.97E-06	-1198.02	Y	0.00	1.00	Y	-0.0
6	1.95E-05	-664.35	Y	0.00	1.00	Y	0.0
7	1.26E-05	-259.58	Y	0.00	1.00	Y	-0.0
8	1.09E-05	-262.76	Y	1.22	0.50	N	-27.7
9	3.88E-05	-68.6	Y	0.18	0.34	N	3.1
10	3.94E-06	-684.02	Y	0.03	0.77	N	-1.0
11	5.58E-06	-1205.53	Y	0.00	1.00	Y	0.0
12	3.72E-05	-488.23	Y	0.00	0.98	Y	-0.0
13	1.18E-05	-225.2	Y	0.00	1.00	Y	-0.0
14	1.52E-05	-447.21	Y	0.00	1.00	Y	0.0
15	7.20E-06	-111.41	Y	0.00	1.00	Y	-0.0
16	4.09E-06	-391.43	Y	0.00	1.00	Y	-0.0
17	3.51E-06	-285.15	Y	0.00	1.00	Y	0.0
18	9.97E-06	-262.92	Y	0.00	1.00	Y	0.0
19	2.79E-05	-192.11	Y	0.00	1.00	Y	0.0
20	8.01E-06	-1478.33	Y	0.00	1.00	Y	-0.0
21	1.55E-05	-297.82	Y	0.00	1.00	Y	-0.0
22	3.01E-06	-981.33	Y	0.00	1.00	Y	-0.0
23	3.16E-06	-704.03	Y	0.00	1.00	Y	-0.0
24	1.30E-05	-333.62	Y	0.00	1.00	Y	0.0
25	3.28E-06	-654.29	Y	0.00	1.00	Y	-0.0
26	8.27E-06	-272.94	Y	0.00	1.00	Y	0.0
27	1.36E-05	-930.22	Y	0.00	1.00	Y	0.0
28	4.68E-05	-293.81	Y	0.00	1.00	Y	0.0
29	2.29E-05	-239.34	Y	0.01	1.00	Y	0.0
30	5.67E-05	-311.78	Y	0.90	0.85	N	0.3

Table S8: Data for TS geometries obtained with **IDPP-TS(H)** on benchmark set

ID	RMS(g) ($E_\mathrm{h}\, a_0{}^{-1}$)	ω_i (cm^{-1})	strict TS?	RMSD (Å)	Overlap	matches ref. TS?	ΔE (kcal/mol)
1	6.00E-05	-1183.66	Y	0.00	1.00	Y	0.0
2	6.16E-06	-201.27	Y	0.00	1.00	Y	-0.0
3	3.37E-05	-146.58	Y	0.00	1.00	Y	-0.0
4	1.05E-05	-770.39	Y	0.00	1.00	Y	0.0
5	4.99E-06	-1197.56	Y	0.00	1.00	Y	-0.0
6	5.78E-05	-667.9	Y	0.00	1.00	Y	0.0
7	1.35E-05	-259.75	Y	0.00	1.00	Y	0.0
8	3.32E-03	-807.03, -103.54, -35.15, -25.31, -18.84, -17.19	N	0.53	0.10	N	-3.5
9	5.23E-05	-648.22	Y	0.00	1.00	Y	0.0
10	6.10E-06	-683.97	Y	0.03	0.77	N	-1.0
11	2.19E-05	-1205.17	Y	0.00	1.00	Y	0.0
12	3.48E-05	-488.18	Y	0.00	1.00	Y	0.0
13	8.44E-06	-543.57	Y	0.26	0.81	N	0.7
14	1.58E-05	-446.81	Y	0.00	1.00	Y	0.0
15	6.87E-06	-111.38	Y	0.00	1.00	Y	-0.0
16	3.18E-06	-391.49	Y	0.00	1.00	Y	-0.0
17	3.95E-06	-285.16	Y	0.00	1.00	Y	-0.0
18	2.84E-04	-66.32, -32.13	N	0.35	0.08	N	-4.4
19	1.59E-03	–	N	0.26	0.00	N	-1.3
20	2.07E-05	-1477.88	Y	0.00	1.00	Y	0.0
21	2.55E-05	-300.28	Y	0.00	1.00	Y	0.0
22	1.27E-05	-981.4	Y	0.00	1.00	Y	-0.0
23	1.18E-05	-703.94	Y	0.00	1.00	Y	0.0
24	3.18E-05	-334.93	Y	0.00	1.00	Y	-0.0
25	5.72E-06	-655.41	Y	0.00	1.00	Y	-0.0
26	3.02E-05	-272.73	Y	0.01	1.00	Y	0.0
27	1.79E-05	-928.51	Y	0.00	1.00	Y	0.0
28	9.00E-06	-294.09	Y	0.00	1.00	Y	-0.0
29	4.42E-05	-239.35	Y	0.02	1.00	Y	0.0
30	7.55E-05	-310.7	Y	0.60	0.84	N	0.3

Table S9: Data for TS geometries obtained with **QCI-HEV** on benchmark set

ID	RMS(g) ($E_\mathrm{h}\,a_0{}^{-1}$)	ω_i (cm^{-1})	strict TS?	RMSD (Å)	Overlap	matches ref. TS?	ΔE (kcal/mol)
1	6.17E-05	-1188.04	Y	0.01	1.00	Y	0.0
2	1.55E-04	-200.05	Y	0.02	1.00	Y	0.0
3	5.26E-05	-151.66	Y	0.02	0.99	Y	0.0
4	9.98E-05	-559.64	Y	0.15	0.94	Y	0.8
5	8.18E-05	-1196.57	Y	0.00	1.00	Y	0.0
6	6.53E-05	-659.79	Y	0.00	1.00	Y	0.0
7	1.45E-04	-260.12	Y	0.01	1.00	Y	0.0
8	9.40E-05	-249.22	Y	0.47	0.71	N	-23.3
9	9.05E-05	-634.52	Y	0.04	1.00	Y	0.1
10	6.73E-05	-680.51	Y	0.03	0.77	N	-1.0
11	9.83E-05	-1207.06	Y	0.00	1.00	Y	0.0
12	7.97E-05	-488.55	Y	0.00	1.00	Y	0.0
13	9.23E-05	-226.08	Y	0.02	1.00	Y	0.0
14	7.59E-05	-442.71	Y	0.03	1.00	Y	0.0
15	5.14E-05	-107.9	Y	0.12	0.90	N	0.1
16	7.08E-05	-391.43	Y	0.00	1.00	Y	0.0
17	1.00E-04	-287.53	Y	0.02	1.00	Y	0.0
18	6.19E-05	-263.38	Y	0.01	1.00	Y	0.0
19	4.39E-05	-29.08, -16.96	N	0.23	0.48	N	1.0
20	8.06E-05	-1274.38	Y	0.04	0.98	Y	0.1
21	1.44E-04	-303.58	Y	0.02	1.00	Y	0.0
22	8.80E-05	-982.41	Y	0.01	1.00	Y	0.0
23	6.26E-05	-702.44	Y	0.01	1.00	Y	0.0
24	1.80E-04	-339.53	Y	0.01	1.00	Y	0.0
25	8.33E-05	-663.69	Y	0.01	1.00	Y	0.0
26	1.04E-04	-273.09	Y	0.02	1.00	Y	0.0
27	7.40E-05	-926.75	Y	0.01	1.00	Y	0.0
28	1.23E-04	-294.35, -3.04	N	0.10	1.00	Y	0.2
29	7.72E-05	-207.08	Y	0.32	0.90	N	0.0
30	2.12E-04	-314.51	Y	0.59	0.88	N	0.3

Table S10: Data for TS geometries obtained with **NEB-TS(H) with 4 images** on benchmark set

ID	RMS(g) ($E_\mathrm{h}\,{a_0}^{-1}$)	ω_i (cm^{-1})	strict TS?	RMSD (Å)	Overlap	matches ref. TS?	ΔE (kcal/mol)
1	3.65e-06	-1183.57	Y	0.00	1.00	Y	0.0
2	1.52e-05	-201.33	Y	0.00	1.00	Y	-0.0
3	2.07e-05	-147.8	Y	0.00	1.00	Y	-0.0
4	9.76e-06	-770.07	Y	0.00	1.00	Y	0.0
5	9.18e-06	-1197.6	Y	0.00	1.00	Y	-0.0
6	1.10e-05	-664.69	Y	0.00	1.00	Y	-0.0
7	1.90e-05	-259.97	Y	0.00	1.00	Y	0.0
8	2.20e-05	-23.23	Y	0.86	0.03	N	-30.7
9	6.61e-06	-642.06	Y	0.00	1.00	Y	0.0
10	1.22e-05	-684.07	Y	0.03	0.77	N	-1.0
11	5.79e-06	-1205.64	Y	0.00	1.00	Y	0.0
12	3.78e-05	-487.78	Y	0.00	1.00	Y	0.0
13	5.11e-06	-225.9	Y	0.00	1.00	Y	0.0
14	1.00e-05	-443.61	Y	0.00	1.00	Y	0.0
15	5.95e-06	-111.42	Y	0.00	1.00	Y	-0.0
16	2.64e-06	-391.51	Y	0.00	1.00	Y	-0.0
17	3.57e-06	-285.14	Y	0.00	1.00	Y	-0.0
18	3.01e-06	-262.86	Y	0.00	1.00	Y	0.0
19	1.35e-05	-193.19	Y	0.00	1.00	Y	-0.0
20	6.32e-06	-1477.97	Y	0.00	1.00	Y	-0.0
21	3.69e-06	-296.89	Y	0.00	1.00	Y	0.0
22	6.80e-06	-982.03	Y	0.00	1.00	Y	-0.0
23	3.33e-06	-703.92	Y	0.00	1.00	Y	0.0
24	1.30e-05	-335.73	Y	0.00	1.00	Y	-0.0
25	3.49e-06	-654.84	Y	0.00	1.00	Y	-0.0
26	5.31e-06	-272.94	Y	0.00	1.00	Y	0.0
27	3.17e-03	-227.61, -69.99, -39.25	N	0.27	0.01	N	-5.7
28	5.86e-05	-293.67	Y	0.00	1.00	Y	-0.0
29	2.98e-05	-242.83	Y	0.01	1.00	Y	0.0
30	7.38e-04	-37.35, -17.9, -5.83	N	0.64	0.13	N	-13.5

Table S11: Data for TS geometries obtained with **NEB-TS(H) with 5 images** on benchmark set

ID	RMS(g) ($E_\mathrm{h}\,{a_0}^{-1}$)	ω_i (cm^{-1})	strict TS?	RMSD (Å)	Overlap	matches ref. TS?	ΔE (kcal/mol)
1	4.51e-06	-1183.53	Y	0.00	1.00	Y	-0.0
2	2.48e-06	-201.33	Y	0.00	1.00	Y	-0.0
3	2.88e-05	-147.67	Y	0.00	1.00	Y	0.0
4	9.24e-06	-770.12	Y	0.00	1.00	Y	0.0
5	5.53e-06	-1197.59	Y	0.00	1.00	Y	-0.0
6	failed		–	–	–	–	–
7	1.64e-05	-259.88	Y	0.00	1.00	Y	0.0
8	7.30e-06	-66.56	Y	0.40	0.20	N	-22.9
9	6.25e-06	-641.51	Y	0.00	1.00	Y	0.0
10	8.15e-06	-684.03	Y	0.03	0.77	N	-1.0
11	6.08e-06	-1205.59	Y	0.00	1.00	Y	0.0
12	4.20e-05	-487.9	Y	0.00	1.00	Y	0.0
13	1.01e-05	-225.9	Y	0.00	1.00	Y	-0.0
14	1.14e-05	-444.68, -5.58	N	0.00	1.00	Y	0.0
15	8.19e-06	-111.4	Y	0.00	1.00	Y	-0.0
16	3.27e-06	-391.51	Y	0.00	1.00	Y	-0.0
17	3.57e-06	-285.14	Y	0.00	1.00	Y	-0.0
18	2.98e-06	-262.88	Y	0.00	1.00	Y	0.0
19	1.31e-05	-187.64	Y	0.00	1.00	Y	-0.0
20	1.25e-05	-1477.03	Y	0.00	1.00	Y	-0.0
21	6.96e-06	-296.78	Y	0.00	1.00	Y	0.0
22	3.63e-06	-981.41	Y	0.00	1.00	Y	-0.0
23	3.18e-06	-703.9	Y	0.00	1.00	Y	0.0
24	1.69e-05	-336.41	Y	0.00	1.00	Y	-0.0
25	3.68e-06	-654.63	Y	0.00	1.00	Y	-0.0
26	8.22e-06	-272.92	Y	0.00	1.00	Y	0.0
27	1.35e-05	-928.75	Y	0.00	1.00	Y	0.0
28	6.60e-05	-293.63	Y	0.00	1.00	Y	0.0
29	7.26e-05	-242.95	Y	0.01	1.00	Y	0.0
30	2.82e-04	-312.14, -8.2	N	0.79	0.97	N	0.0

Table S12: Data for TS geometries obtained with **NEB-TS(H) with coarser convergence** (i.e. doubled convergence thresholds) on benchmark set

ID	RMS(g) ($E_\mathrm{h}\,{a_0}^{-1}$)	ω_i (cm^{-1})	strict TS?	RMSD (Å)	Overlap	matches ref. TS?	ΔE (kcal/mol)
1	3.95e-06	-1183.56	Y	0.00	1.00	Y	0.0
2	2.46e-05	-201.43	Y	0.00	1.00	Y	-0.0
3	2.07e-05	-147.81	Y	0.00	1.00	Y	-0.0
4	9.97e-06	-770.35	Y	0.00	1.00	Y	0.0
5	4.25e-06	-1197.68	Y	0.00	1.00	Y	-0.0
6	1.30e-05	-664.94	Y	0.00	1.00	Y	0.0
7	1.40e-05	-259.68	Y	0.00	1.00	Y	0.0
8	5.20e-05	-254.97, -50.82	N	1.22	0.52	N	-27.5
9	8.44e-06	-641.95	Y	0.00	1.00	Y	0.0
10	4.81e-06	-683.84	Y	0.03	0.77	N	-1.0
11	5.51e-06	-1205.62	Y	0.00	1.00	Y	0.0
12	3.60e-05	-488.0	Y	0.00	1.00	Y	0.0
13	5.02e-06	-225.83	Y	0.00	1.00	Y	-0.0
14	1.14e-05	-442.81, -5.85	N	0.00	1.00	Y	0.0
15	5.00e-06	-111.41	Y	0.00	1.00	Y	-0.0
16	3.88e-06	-391.51	Y	0.00	1.00	Y	-0.0
17	6.17e-06	-285.19	Y	0.00	1.00	Y	-0.0
18	2.84e-06	-262.88	Y	0.00	1.00	Y	0.0
19	2.51e-05	-184.53	Y	0.00	1.00	Y	0.0
20	2.76e-05	-1477.06	Y	0.00	1.00	Y	0.0
21	8.14e-06	-297.01	Y	0.00	1.00	Y	0.0
22	5.29e-06	-980.99	Y	0.00	1.00	Y	-0.0
23	7.72e-06	-703.21	Y	0.00	1.00	Y	0.0
24	1.18e-05	-334.71	Y	0.00	1.00	Y	-0.0
25	4.21e-06	-654.77	Y	0.00	1.00	Y	-0.0
26	2.13e-05	-272.77	Y	0.00	1.00	Y	0.0
27	1.28e-05	-930.74	Y	0.00	1.00	Y	0.0
28	6.11e-05	-293.54	Y	0.01	1.00	Y	0.0
29	2.68e-05	-241.72	Y	0.01	1.00	Y	-0.0
30	4.51e-05	-309.11	Y	0.92	0.73	N	0.3

Table S13: Data for TS geometries obtained with **NEB-TS(H) with uniform spring** on benchmark set

ID	RMS(g) ($E_\mathrm{h}\, {a_0}^{-1}$)	ω_i (cm^{-1})	strict TS?	RMSD (Å)	Overlap	matches ref. TS?	ΔE (kcal/mol)
1	3.81e-06	-1183.59	Y	0.00	1.00	Y	0.0
2	2.70e-06	-201.35	Y	0.00	1.00	Y	-0.0
3	2.15e-05	-147.64	Y	0.00	1.00	Y	-0.0
4	9.68e-06	-769.96	Y	0.00	1.00	Y	0.0
5	4.70e-06	-1197.78	Y	0.00	1.00	Y	-0.0
6	failed		–	–	–	–	–
7	1.73e-05	-259.96	Y	0.00	1.00	Y	0.0
8	8.92e-06	-84.49	Y	0.94	0.14	N	-24.1
9	2.39e-05	-14.64	Y	0.37	0.05	N	-4.0
10	4.47e-06	-684.01	Y	0.03	0.77	N	-1.0
11	5.60e-06	-1205.64	Y	0.00	1.00	Y	0.0
12	failed		–	–	–	–	–
13	3.66e-06	-225.89	Y	0.00	1.00	Y	0.0
14	9.69e-06	-444.17, -2.69	N	0.00	1.00	Y	0.0
15	5.78e-06	-111.39	Y	0.09	0.95	Y	0.0
16	failed		–	–	–	–	–
17	5.03e-06	-285.14	Y	0.00	1.00	Y	-0.0
18	3.26e-06	-262.87	Y	0.00	1.00	Y	0.0
19	8.77e-06	-189.28	Y	0.00	1.00	Y	-0.0
20	9.64e-04	–	N	0.18	0.00	N	-10.5
21	9.15e-06	-295.92	Y	0.00	1.00	Y	0.0
22	5.12e-06	-981.27	Y	0.00	1.00	Y	-0.0
23	3.17e-06	-703.93	Y	0.00	1.00	Y	0.0
24	2.01e-05	-333.85	Y	0.00	1.00	Y	-0.0
25	2.60e-06	-654.61	Y	0.00	1.00	Y	-0.0
26	2.27e-05	-272.93	Y	0.00	1.00	Y	0.0
27	3.20e-04	-212.04	Y	0.38	0.01	N	-13.5
28	6.62e-05	-293.76	Y	0.01	1.00	Y	0.0
29	1.33e-05	-32.31	Y	0.91	0.20	N	21.4
30	4.18e-04	-39.9, -26.26, -10.39	N	0.55	0.09	N	-14.2

4.3.2 Timings and gradient calls for TS search

The data on the time required for each method to finish, the number of gradient evaluations performed during the double-ended step and the TS optimization step, and the total number of iterations during the double-ended step are shown below.

Table S14: Total run-times for hierarchical double-ended TS search methods over the benchmark reactions (in units of hours).

	NEB-TS	NEB-TS(H)	DNEB-HEV	GSM-EV-ES	DHS-TS(H)	i-EIP-TS(H)	IDPP-TS(H)	QCI-HEV
1	36.81	37.44	25.84	43.73	15.40	16.50	17.61	18.28
2	26.10	14.35	17.29	20.03	10.21	12.00	5.35	14.05
3	2.60	2.78	13.70	4.56	2.07	1.65	1.07	6.92
4	17.10	15.18	17.68	12.98	6.78	14.41	3.13	10.25
5	18.82	15.74	10.27	24.79	5.37	4.02	4.99	6.19
6	58.41	57.15	38.79	23.63	13.67	13.02	7.32	12.74
7	10.22	9.41	7.41	7.19	5.50	3.70	2.02	7.93
8	76.98	80.89	143.88	48.00	62.05	64.93	31.60	62.66
9	76.06	73.91	58.05	42.93	32.09	48.14	24.13	28.36
10	57.23	56.42	43.68	77.32	14.06	15.50	9.91	24.36
11	9.50	9.37	9.11	7.84	6.90	3.12	1.65	6.90
12	5.55	5.85	10.31	9.31	3.44	3.80	1.27	4.04
13	20.88	18.37	30.31	18.69	15.82	17.99	9.07	20.98
14	13.58	12.80	29.42	8.38	11.38	25.42	4.04	13.26
15	21.51	9.75	14.93	10.60	8.17	11.69	6.58	10.49
16	40.71	39.90	48.45	47.88	23.59	73.24	8.52	33.45
17	12.30	11.95	14.13	29.08	9.30	20.62	3.78	7.63
18	13.53	13.48	16.42	51.19	10.76	10.53	16.53	13.23
19	16.20	16.84	26.53	6.67	12.51	14.17	9.71	12.19
20	20.93	20.90	27.98	42.75	17.09	26.98	4.87	17.41
21	16.01	16.72	27.24	18.75	12.84	11.11	6.04	17.07
22	16.76	15.59	9.69	13.82	6.80	7.04	6.04	7.14
23	23.24	10.68	16.78	9.00	7.84	18.11	2.70	13.42
24	1.34	0.99	1.45	1.61	1.10	1.33	0.61	1.00
25	7.27	4.98	6.56	9.48	4.13	3.11	1.44	6.77
26	16.51	15.07	22.46	10.28	6.95	6.54	2.53	8.68
27	19.81	20.63	26.04	31.69	13.11	12.68	5.50	15.98
28	21.91	18.23	17.67	14.25	16.68	13.53	4.17	9.69
29	26.19	25.39	25.13	13.59	6.72	13.55	4.42	10.20
30	29.78	28.32	67.34	23.31	20.63	26.82	9.82	24.50

Table S15: Number of gradient evaluations in the **double-ended** step of the hierarchical double-ended TS search over the benchmark reactions. NEB-TS(H) variations - NEB-TS(H)-4/5 for 4/5 images, NEB-TS(H)-conv. for coarser convergence criteria, and NEB-TS(H)-spr. for uniform springs.

	NEB-TS	NEB-TS(H)	DNEB-HEV	GSM-EV-ES	DHS-TS(H)	i-EIP-TS(H)	IDPP-TS(H)	QCI-HEV	NEB-TS(H)-4	NEB-TS(H)-5	NEB-TS(H)-conv.	NEB-TS(H)-spr.
1	274	274	90	211	90	100	10	10	314	290	210	434
2	154	154	50	257	110	152	10	10	186	194	90	178
3	146	146	378	163	87	80	10	10	154	186	82	114
4	266	266	242	236	114	126	10	10	314	234	130	506
5	314	314	114	401	69	54	10	10	290	290	234	410
6	1226	1226	562	357	212	216	10	10	2242	4002	474	4002
7	322	322	34	127	74	74	10	10	202	122	98	154
8	490	490	434	265	291	322	10	10	306	610	354	642
9	746	746	346	385	206	330	10	10	1050	610	330	1154
10	690	690	234	874	99	88	10	10	490	714	354	778
11	186	186	82	132	59	38	10	10	218	186	154	330
12	474	474	562	566	242	284	10	10	530	514	370	4002
13	314	314	234	243	218	284	10	10	482	378	226	818
14	218	218	234	171	128	178	10	10	378	346	186	834
15	162	162	146	122	116	156	10	10	178	170	114	170
16	402	402	306	330	204	368	10	10	802	578	242	4002
17	194	194	106	453	109	122	10	10	314	258	138	210
18	210	210	42	781	105	110	10	10	266	194	146	258
19	346	346	394	195	221	278	10	10	226	250	258	354
20	218	218	162	512	126	128	10	10	218	218	122	1074
21	178	178	106	154	73	50	10	10	218	218	162	170
22	330	330	98	233	100	110	10	10	330	274	154	210
23	186	186	122	124	130	210	10	10	410	130	178	314
24	178	178	146	186	121	94	10	10	170	170	106	74
25	106	106	18	193	64	62	10	10	170	210	50	90
26	602	602	562	275	212	206	10	10	490	458	258	482
27	314	314	250	567	165	174	10	10	586	818	226	978
28	274	274	194	242	125	144	10	10	210	370	178	274
29	946	946	562	437	219	298	10	10	770	914	642	2274
30	482	482	562	539	324	446	10	10	858	626	282	386

Table S16: Number of gradient evaluations in the **TS optimization** step of the hierarchical double-ended TS search over the benchmark reactions. (Hessian-corrected for methods which calculate one analytic Hessian at the beginning i.e. methods ending with H in parentheses.) NEB-TS(H) variations - NEB-TS(H)-4/5 for 4/5 images, NEB-TS(H)-conv. for coarser convergence criteria, and NEB-TS(H)-spr. for uniform springs.

	NEB-TS	NEB-TS(H)	DNEB-HEV	GSM-EV-ES	DHS-TS(H)	i-EIP-TS(H)	IDPP-TS(H)	QCI-HEV	NEB-TS(H)-4	NEB-TS(H)-5	NEB-TS(H)-conv.	NEB-TS(H)-spr.
1	21	21	159	220	37	30	170	187	20	21	22	18
2	302	54	278	88	44	37	84	279	62	68	66	47
3	29	18	547	141	26	16	62	519	23	25	30	16
4	143	44	200	104	34	39	72	328	42	42	46	36
5	36	25	116	158	43	35	57	174	26	25	28	22
6	30	22	288	140	57	46	174	369	31	8	38	8
7	68	42	331	176	34	36	72	359	87	82	46	83
8	267	310	1461	258	204	198	310	894	202	53	310	112
9	63	73	342	199	111	88	313	380	69	77	90	222
10	39	30	332	132	64	47	128	387	34	33	45	26
11	27	22	156	67	105	25	35	181	22	22	25	22
12	149	95	290	433	53	43	118	537	83	94	90	6
13	110	63	398	182	104	77	198	526	47	66	65	61
14	53	50	376	12	105	64	85	363	39	57	49	52
15	302	44	210	120	31	68	131	304	44	33	51	38
16	73	49	274	258	50	44	102	481	49	50	61	13
17	32	23	177	240	57	25	76	194	20	22	27	22
18	28	29	286	160	27	29	312	303	30	33	29	27
19	219	240	533	42	147	170	313	514	147	168	279	175
20	74	67	245	165	118	71	71	310	48	61	79	314
21	37	45	283	127	104	107	80	280	41	38	38	33
22	49	38	158	126	54	46	154	219	35	41	63	35
23	302	44	286	96	22	28	51	411	19	45	43	24
24	119	87	221	100	44	144	94	288	94	93	99	92
25	119	42	234	124	57	26	37	235	29	28	50	28
26	109	47	405	178	55	57	98	426	47	48	63	43
27	62	67	333	120	72	62	106	414	311	51	57	311
28	294	191	254	128	311	188	96	273	209	131	205	194
29	132	127	640	179	44	100	173	570	127	91	211	38
30	302	313	2520	180	250	311	313	979	313	313	313	313

Table S17: Number of iterations in **double-ended** step of the hierarchical double-ended TS search over the benchmark reactions. NEB-TS(H) variations - NEB-TS(H)-5 for 5 images, NEB-TS(H)-conv. for coarser convergence criteria, and NEB-TS(H)-spr. for uniform springs.

	NEB-TS	NEB-TS(H)	DNEB-HEV	GSM-EV-ES	DHS-TS(H)	i-EIP-TS(H)	IDPP-TS(H)	QCI-HEV	NEB-TS(H)-5	NEB-TS(H)-conv.	NEB-TS(H)-spr.
1	34	34	11	12	90	50	1	1	36	26	54
2	19	19	6	17	110	76	1	1	24	11	22
3	18	18	47	10	87	40	1	1	23	10	14
4	33	33	30	17	114	63	1	1	29	16	63
5	39	39	14	24	69	27	1	1	36	29	51
6	153	153	70	26	212	108	1	1	500	59	500
7	40	40	4	10	74	37	1	1	15	12	19
8	61	61	54	17	291	161	1	1	76	44	80
9	93	93	43	28	206	165	1	1	76	41	144
10	86	86	29	43	99	44	1	1	89	44	97
11	23	23	10	8	59	19	1	1	23	19	41
12	59	59	70	41	242	142	1	1	64	46	500
13	39	39	29	14	218	142	1	1	47	28	102
14	27	27	29	10	128	89	1	1	43	23	104
15	20	20	18	8	116	78	1	1	21	14	21
16	50	50	38	23	204	184	1	1	72	30	500
17	24	24	13	37	109	61	1	1	32	17	26
18	26	26	5	44	105	55	1	1	24	18	32
19	43	43	49	11	221	139	1	1	31	32	44
20	27	27	20	28	126	64	1	1	27	15	134
21	22	22	13	8	73	25	1	1	27	20	21
22	41	41	12	13	100	55	1	1	34	19	26
23	23	23	15	9	130	105	1	1	16	22	39
24	22	22	18	11	121	47	1	1	21	13	9
25	13	13	2	11	64	31	1	1	26	6	11
26	75	75	70	16	212	103	1	1	57	32	60
27	39	39	31	44	165	87	1	1	102	28	122
28	34	34	24	15	125	72	1	1	46	22	34
29	118	118	70	25	219	149	1	1	114	80	284
30	60	60	70	37	324	223	1	1	78	35	48

4.4 Parameter choices for NEB-TS(H)

Along with the parameter choices of NEB-TS(H) tested in the main text, we also show results for NEB-TS(H) run with 4 images. The results are given below:

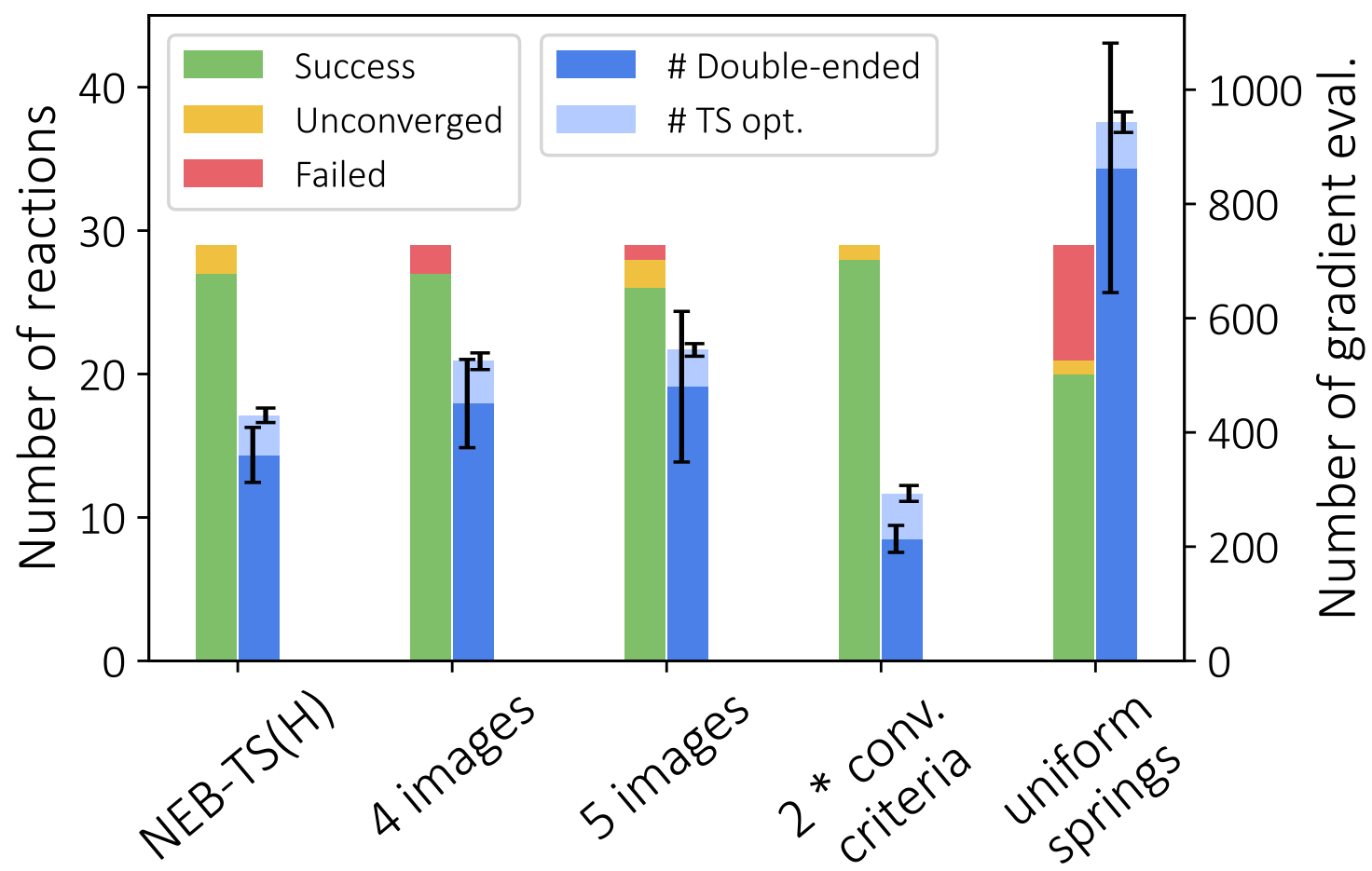

Figure S3: Success rates and average number of gradient evaluations for NEB-TS(H) under different settings, varying one parameter at a time (from left to right): (i) default setting with 8 images (ii) 4 images, (iii) 5 images (iv) 8 images but convergence thresholds relaxed by a factor of two (v) uniform springs instead of energy-weighting springs. Reported image numbers exclude the fixed reactant and product endpoints.

When NEB-TS(H) is run with four images, reaction 27 and reaction 30 fail. The computational cost is also higher, particularly in the double-ended step, as shown by the increase in average number of gradient evaluations (CI-NEB) from 361 for the default of 8 images to 480 for four-image calculations (and 451 for five-image).

4.5 Cost-accuracy trade-off

We have assigned success scores to various outcomes in order to compare all methods consistently: a successful TS is assigned 1, unconverged TSs to 0.7 and failures to 0. The average success score for a method is plotted against the average number of gradient evaluations for that method. The score for unconverged TS is somewhat arbitrary, so we have also plotted the cost-accuracy trade-off using various values (0.6 and 0.8) for the unconverged TSs.

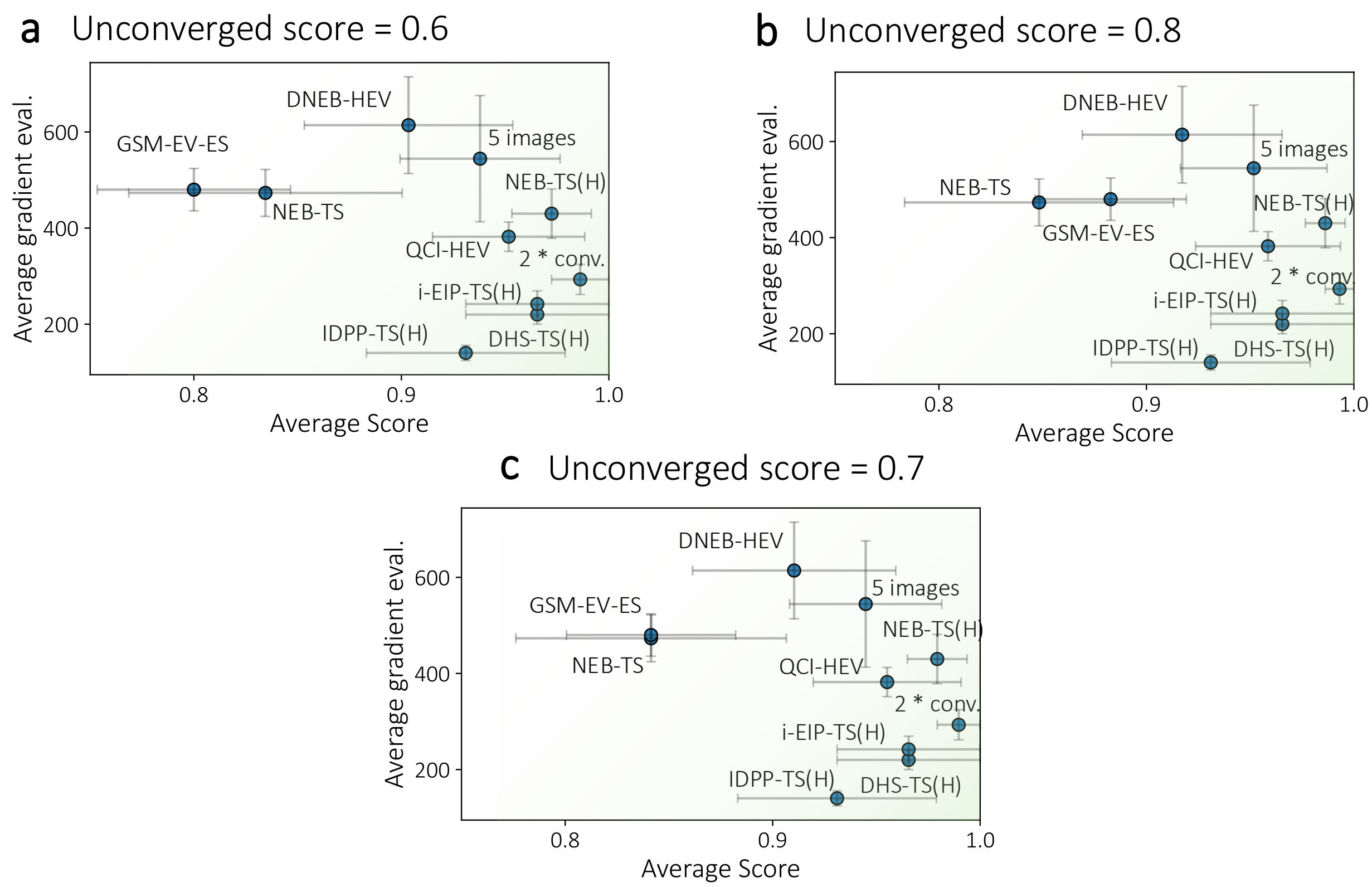

Figure S4: The cost-accuracy trade-off plotted using success scores calculated with various values for the unconverged TSs (0.6, 0.7 and 0.8). The error bars show the standard error of mean along the respective axis.

While changing the score for unconverged TSs does change relative positions of the methods on the plot, the main conclusions remain unchanged. The methods with good success rate and good efficiency are IDPP-TS(H), DHS-TS(H), i-EIP-TS(H) and NEB-TS(H) with coarser convergence. QCI-HEV and NEB-TS(H) also show good performance but are more costly. These trends are maintained in all plots.

4.6 TS conformers

Among the 220 successful or unconverged TSs, there were 14 cases of TS conformers. In case of reaction-30, calculations with the methods NEB-TS, NEB-TS(H) and GSM-EV-ES found geometries which were unconverged. However, reoptimizing them led to conformers of the reference TS and thus they are counted as TS conformers.

For all reactions where we obtain conformers, the thermochemical parameters are provided below, including relative values of electronic energies (ΔE), zero point energies (ΔZPE), enthalpies (ΔH), entropy contributions ($T\Delta S$) and Gibbs free energies (ΔG). The imaginary frequencies (ω_i) are also provided for the TSs. Note that here, RS and PS indicate the reactant and product as shown in the reaction schemes in main text in Table-1.

Table S18: **Reaction-13**. Thermochemical parameters for RS, PS and all TS conformers at PBE0-D3BJ/def2-SVP level of theory, in units of kcal mol^{-1}. Imaginary frequencies in units of cm^{-1} are also quoted for the TSs.

	ΔE	ΔZPE	ΔH	$T\Delta S$	ΔG	ω_i
RS	0	0	0	0	0	–
PS	17.2	-0.1	17.4	1.3	16.1	–
Reference TS	38.4	-1.8	36.5	0.4	36.1	-225.76
IDPP-TS(H)	39.1	-2.9	35.9	-0.4	36.4	-543.57

Table S19: **Reaction-15**. Thermochemical parameters for RS, PS and all TS conformers at PBE0-D3BJ/def2-SVP level of theory, in units of kcal mol^{-1}. Imaginary frequencies in units of cm^{-1} are also quoted for the TSs.

	ΔE	ΔZPE	ΔH	$T\Delta S$	ΔG	ω_i
RS	0	0	0	0	0	–
PS	0.0	0.0	0.0	0.0	0.0	–
Reference TS	10.8	0.3	10.4	-1.7	12.1	-111.37
QCI-HEV	10.9	0.3	10.4	-1.6	12.1	-107.9

Table S20: **Reaction-20**. Thermochemical parameters for RS, PS and all TS conformers at PBE0-D3BJ/def2-SVP level of theory, in units of kcal mol^{-1}. Imaginary frequencies in units of cm^{-1} are also quoted for the TSs.

	ΔE	ΔZPE	ΔH	$T\Delta S$	ΔG	ω_i
RS	0	0	0	0	0	–
PS	11.6	-1.7	10.1	0.6	9.5	–
Reference TS	23.7	-6.7	16.0	-1.8	17.8	−1478.59
DHS-TS(H)	23.2	-6.1	16.1	-1.9	18.0	−1338.29

Table S21: **Reaction-21**. Thermochemical parameters for RS, PS and all TS conformers at PBE0-D3BJ/def2-SVP level of theory, in units of kcal mol^{-1}. Imaginary frequencies in units of cm^{-1} are also quoted for the TSs.

	ΔE	ΔZPE	ΔH	$T\Delta S$	ΔG	ω_i
RS	0	0	0	0	0	–
PS	-12.1	1.2	-11.1	0.1	-11.2	–
Reference TS	14.0	-2.5	10.2	-2.4	12.6	−297.16
DHS-TS(H)	13.8	-3.7	8.9	-2.1	10.9	−992.98

Table S22: **Reaction-24**. Thermochemical parameters for RS, PS and all TS conformers at PBE0-D3BJ/def2-SVP level of theory, in units of kcal mol^{-1}. Imaginary frequencies in units of cm^{-1} are also quoted for the TSs.

	ΔE	ΔZPE	ΔH	$T\Delta S$	ΔG	ω_i
RS	0	0	0	0	0	–
PS	-3.8	-1.3	-2.4	0.2	-2.6	–
Reference TS	6.4	-0.8	5.5	0.1	5.5	−334.00
DHS-TS(H)	7.4	-1.3	5.9	-0.4	6.3	−844.32

Table S23: **Reaction-29**. Thermochemical parameters for RS, PS and all TS conformers at PBE0-D3BJ/def2-SVP level of theory, in units of kcal mol^{-1}. Imaginary frequencies in units of cm^{-1} are also quoted for the TSs.

	ΔE	ΔZPE	ΔH	$T\Delta S$	ΔG	ω_i
RS	0	0	0	0	0	–
PS	-36.5	2.1	-34.6	0.1	-34.6	–
Reference TS	18.9	-1.4	17.6	0.6	17.1	−241.51
NEB-TS	18.9	-1.5	17.6	0.4	17.1	−207.49
QCI-HEV	18.9	-1.5	17.6	0.4	17.1	−207.08

Table S24: **Reaction-30**. Thermochemical parameters for RS, PS and all valid TS conformers at PBE0-D3BJ/def2-SVP level of theory, in units of kcal mol^{-1}. Imaginary frequencies in units of cm^{-1} are also quoted for the TSs. The TSs marked (reopt.) have been reoptimized in ORCA starting from the double-ended TSs, which had more than one imaginary mode (i.e. not strict saddle points).

	ΔE	ΔZPE	ΔH	$T\Delta S$	ΔG	ω_i
RS	0	0	0	0	0	–
PS	-69.7	3.5	-67.9	-4.2	-63.7	–
Reference TS	17.4	-0.1	16.1	-3.0	19.1	−310.03
NEB-TS (reopt.)	17.4	-0.1	16.1	-2.8	18.9	−308.53
NEB-TS(H) (reopt.)	17.4	-0.1	16.1	-2.9	19.0	−308.87
GSM-EV-ES (reopt.)	17.4	-0.1	16.1	-2.9	19.0	−308.74
DHS-TS(H)	17.4	-0.1	16.1	-3.0	19.1	−309.89
i-EIP-TS(H)	17.7	-0.2	16.4	-2.9	19.2	−311.78
IDPP-TS(H)	17.7	-0.1	16.4	-2.8	19.2	−310.70
QCI-HEV	17.7	-0.1	16.4	-3.0	19.4	−314.51
NEB-TS	71.8	–	–	–	–	−340.72, −101.33, −45.81, −42.49, −22.30
NEB-TS(H)	17.7	–	–	–	–	−308.44, −20.05
GSM-EV-ES	25.7	–	–	–	–	−359.07, −52.79, −32.62, −4.33

4.7 Analysis of reaction parameters

The reaction descriptors analyzed for possible correlations to the success rate and efficiency of hierarchical double-ended methods are described in detail below.

1. **Number of atoms** – This describes the size of the system being modeled during the reaction

2. **RMSD(RS-PS)** – The RMSD between the reference RS and reference PS, with only heavy atoms and any hydrogen which is participating in the reaction. This descriptor indicates the similarity between the reactant and product.

3. **RMSD($\mathbf{TS_{ref}}$-$\mathbf{TS_{guess}}$)** – The RMSD between the TS guess from the double-ended method and the reference TS. This indicates the quality of the TS guess and how close it is to the true TS.

4. $\boldsymbol{\Delta G^{\ddagger}}$ – The Gibbs free energy of activation for the reference reaction. Since RS and PS are interchangeable for double-ended methods, we have calculated both forward and backwards barriers and used whichever is lower.

5. $\boldsymbol{\omega_i}$ – The imaginary frequency for the reference TS.

6. **Lateness of TS** – The lateness is defined as RMSD(RS_{ref}-TS_{ref}) – RMSD(TS_{ref}-PS_{ref}). If the TS is closer to the RS than the PS, then the parameter will be negative and vice versa.

Please note that for both RMSD(RS-PS) and RMSD(TS_{ref}-TS_{guess}), we consider only heavy atoms, and any hydrogens actively participating in the reaction. The values of the descriptors for each reaction are provided underneath, along with plots of success rate and number of gradient evaluations (double-ended step, TS optimization step, and overall). Note that we have used gradient evaluations rather than run-time, since run-time is dependent on the level of theory, and the size of the system. This means it would be difficult to separate the influence of the choice of TS search method, and the choice of level of theory or system size.

Table S25: **Reaction descriptors**. Number of atoms, RMSD(RS-PS), $\Delta G^{\ddagger}$, Lateness of TS and ω_i for each reaction.

ID	Number of atoms	RMSD(RS-PS) (Å)	$\Delta G^{\ddagger}$ (kcal mol^{-1})	Lateness of TS (Å)	ω_i (cm^{-1})
1	65	0.32	10.96	-0.03	-1183.44
2	51	0.38	5.17	0.13	-201.34
3	23	0.30	1.43	-0.15	-146.71
4	38	0.29	3.58	-0.06	-770.17
5	37	0.24	6.55	-0.07	-1197.72
6	37	0.57	12.11	-0.06	-664.83
7	32	0.35	2.35	0.07	-259.59
8	54	0.87	37.73	0.30	-359.88
9	57	0.72	9.91	0.10	-643.38
10	50	0.40	37.43	-0.02	-1069.77
11	39	0.19	4.95	-0.05	-1205.92
12	19	0.73	19.55	-0.54	-487.9
13	41	0.78	19.99	-0.39	-225.76
14	41	0.36	0.50	0.06	-445.85
15	45	0.49	12.11	-0.02	-111.37
16	60	0.66	13.72	-0.48	-391.5
17	38	0.41	21.23	-0.05	-285.15
18	43	0.29	8.48	-0.12	-262.89
19	39	0.66	3.98	-0.08	-191.8
20	51	0.33	8.33	0.08	-1478.59
21	53	0.22	12.60	0.04	-297.16
22	43	0.40	8.48	-0.07	-981.48
23	43	0.51	7.63	-0.27	-704.05
24	15	0.42	5.48	-0.20	-334
25	33	0.24	2.48	0.04	-654.38
26	31	0.74	14.91	0.02	-272.9
27	38	0.56	12.06	-0.14	-929.32
28	38	0.41	13.65	0.05	-294.25
29	27	0.78	17.09	0.56	-241.51
30	35	0.95	19.08	-0.24	-310.03

Table S26: **Reaction descriptor: RMSD($\mathbf{TS_{ref}}$-$\mathbf{TS_{guess}}$)**. The values are given in units of Å. For GSM-EV-ES, the software package does not output the TS guess geometry, therefore it is not available.

	NEB-TS	NEB-TS(H)	DNEB-HEV	GSM-EV-ES	DHS	i-EIP	IDPP-TS(H)	QCI-HEV
1	0.04	0.04	0.07	–	0.08	0.06	0.11	0.11
2	0.11	0.11	0.12	–	0.08	0.10	0.13	0.13
3	0.03	0.03	0.05	–	0.05	0.03	0.07	0.07
4	0.07	0.07	0.06	–	0.04	0.07	0.13	0.12
5	0.03	0.03	0.05	–	0.04	0.05	0.09	0.09
6	0.04	0.04	0.16	–	0.14	0.15	0.25	0.23
7	0.16	0.16	0.18	–	0.08	0.13	0.18	0.18
8	0.38	0.38	0.50	–	0.60	0.42	0.49	0.39
9	0.20	0.20	0.31	–	0.36	0.26	0.29	0.29
10	0.10	0.10	0.18	–	0.14	0.18	0.22	0.21
11	0.06	0.06	0.06	–	0.07	0.07	0.08	0.08
12	0.29	0.29	0.22	–	0.16	0.26	0.39	0.38
13	0.17	0.17	0.16	–	0.17	0.19	0.23	0.23
14	0.14	0.14	0.19	–	0.22	0.15	0.16	0.16
15	0.06	0.06	0.07	–	0.05	0.05	0.08	0.12
16	0.16	0.16	0.12	–	0.05	0.18	0.27	0.28
17	0.05	0.05	0.08	–	0.06	0.06	0.10	0.10
18	0.07	0.07	0.08	–	0.05	0.06	0.10	0.10
19	0.27	0.27	0.26	–	0.19	0.24	0.28	0.26
20	0.08	0.08	0.09	–	0.20	0.08	0.11	0.11
21	0.06	0.06	0.07	–	0.08	0.08	0.09	0.09
22	0.06	0.06	0.07	–	0.08	0.06	0.12	0.07
23	0.14	0.14	0.16	–	0.06	0.12	0.17	0.21
24	0.12	0.12	0.19	–	0.26	0.17	0.16	0.17
25	0.07	0.07	0.08	–	0.09	0.07	0.08	0.08
26	0.16	0.16	0.19	–	0.21	0.18	0.27	0.26
27	0.18	0.18	0.18	–	0.24	0.18	0.22	0.22
28	0.15	0.15	0.17	–	0.15	0.15	0.19	0.19
29	0.30	0.30	0.32	–	0.11	0.26	0.40	0.39
30	0.66	0.66	0.55	–	0.56	0.65	0.71	0.71

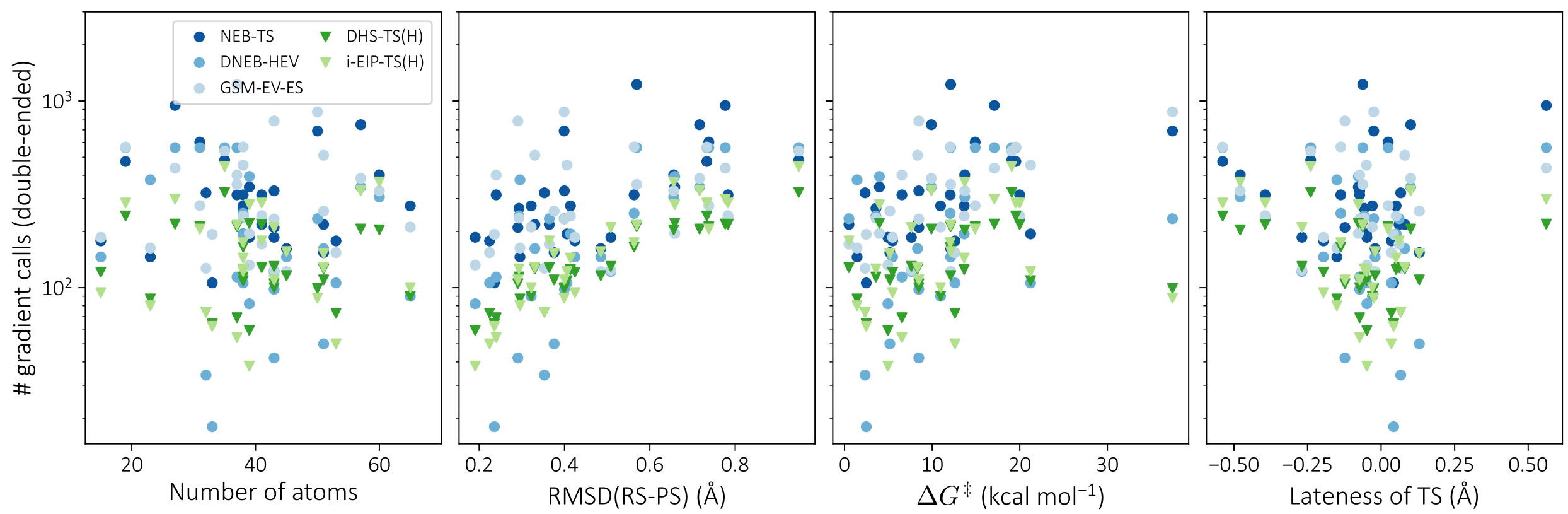

Figure S5: Number of gradient evaluations during the double-ended step plotted against various reaction descriptors.

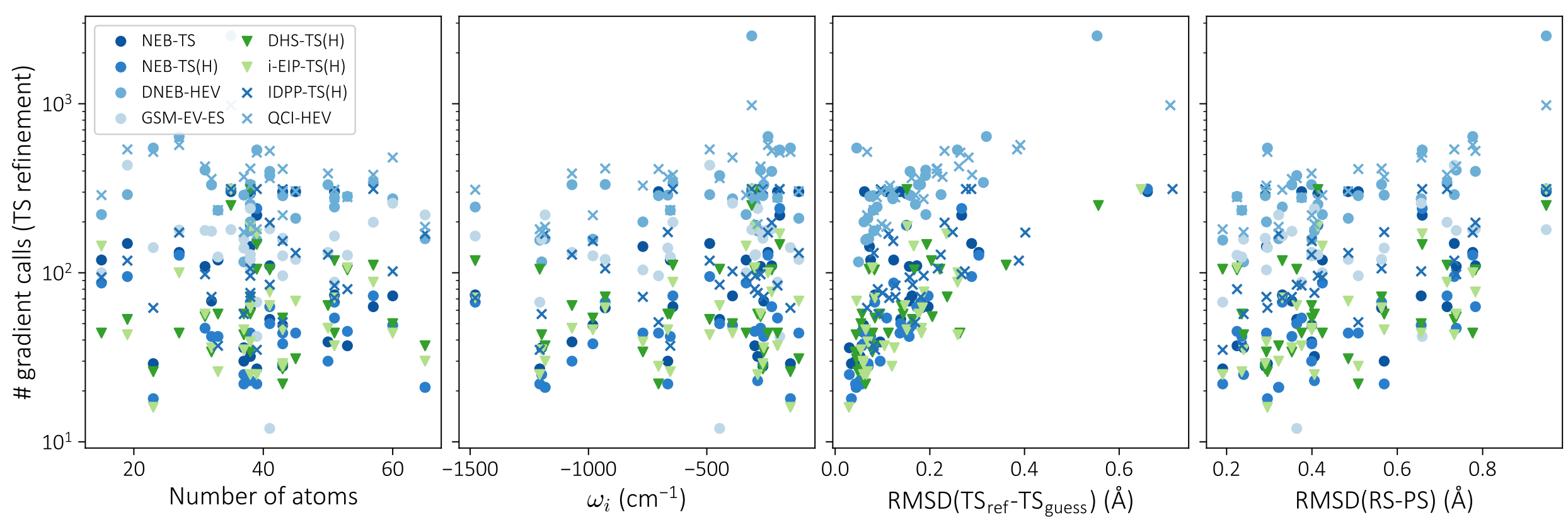

Figure S6: Number of gradient evaluations during the TS refinement step plotted against various reaction descriptors.

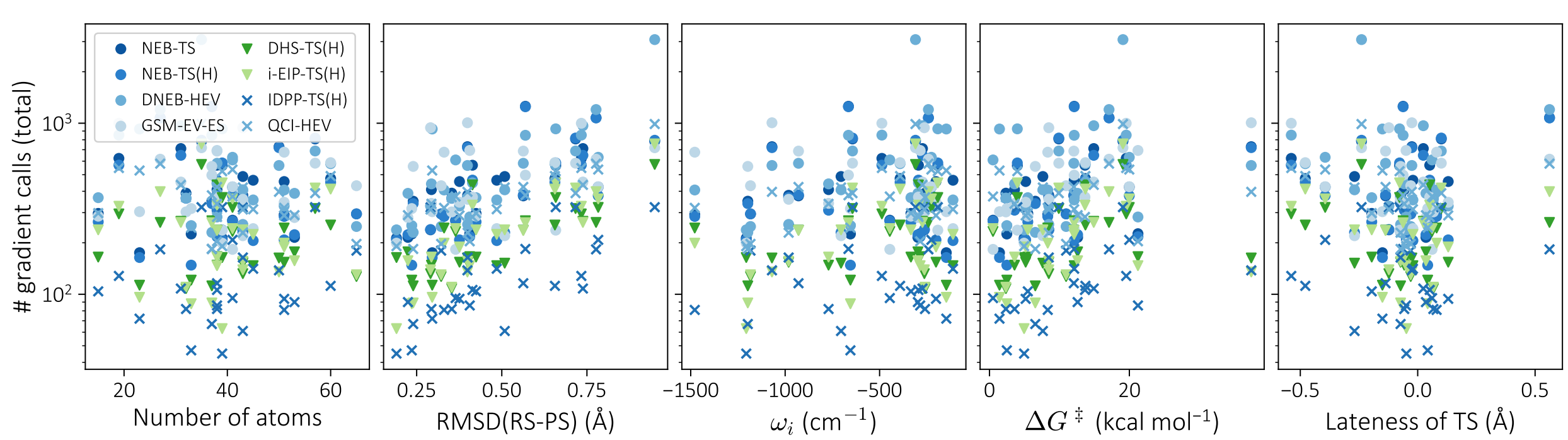

Figure S7: Total number of gradient evaluations during hierarchical TS search plotted against various reaction descriptors.

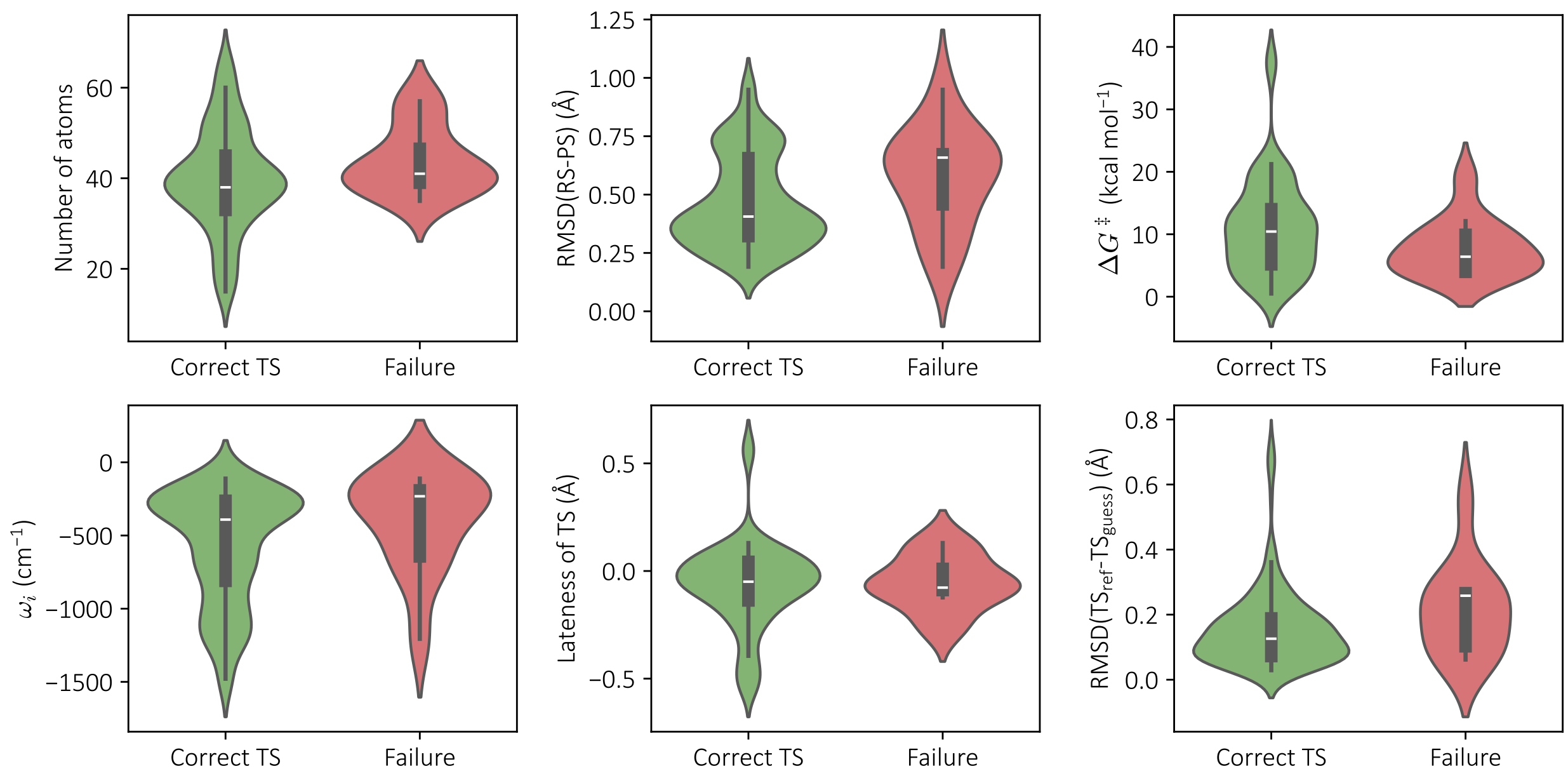

Figure S8: The distribution of various reaction descriptors for correct TSs found (successful + unconverged) and failures.

5 Case studies

5.1 Pd-catalyzed multi-step C-H activation

All calculations were performed at the PBE0-D3BJ/def2-SVP level of theory, consistent with the rest of our benchmark.

Table S27: **Pd cat. C-H activation**. Thermochemical parameters for RS, PS and TSs at PBE0-D3BJ/def2-SVP level of theory, in units of kcal mol^{-1}. Imaginary frequencies for all TSs are quoted in units of cm^{-1}. The number of gradient evaluations in double-ended step (N_{dbl}) and TS refinement (N_{ts}, Hessian-corrected) for each hierarchical method is shown, for each TS.

	ΔE	ΔZPE	ΔH	$T\Delta S$	ΔG	ω_i	N_{dbl}	N_{ts}
RS	0.0	0.0	0.0	0.0	0.0	–		
PS	4.9	0.3	5.7	0.9	4.8	–		
RS'	0.0	0.0	0.6	1.6	-1.0	–		
$\mathrm{INT_A}$	-3.3	0.2	-2.9	0.3	-3.2	–		
$\mathrm{INT_B}$	-5.3	0.4	-4.5	0.5	-5.0	–		
$\mathbf{TS_A}$								
IDPP-TS(H)	7.5	-0.3	7.3	0.1	7.2	-125.8	10	107
DHS-TS(H)	7.5	-0.3	7.3	0.1	7.2	-126.1	113	39
i-EIP-TS(H)	7.5	-0.3	7.3	0.1	7.2	-125.8	96	40
QCI-HEV (reopt.)	7.5	-0.3	7.3	0.6	6.8	-125.9	10	582
$\mathbf{TS_B}$								
IDPP-TS(H)	9.6	-3.0	6.6	-0.4	6.9	-754.9	10	200
DHS-TS(H)	9.6	-3.0	6.6	-0.4	7.0	-761.8	235	62
i-EIP-TS(H)	9.6	-3.0	6.6	-0.4	6.9	-756.4	252	79
QCI-HEV	9.6	-2.9	6.6	-0.4	7.0	-740.6	10	500
$\mathbf{TS_C}$								
IDPP-TS(H)	11.6	-0.8	11.1	0.5	10.7	-622.4		
DHS-TS(H)	11.6	-0.8	11.1	0.5	10.7	-622.0		
i-EIP-TS(H)	11.6	-0.8	11.1	0.5	10.7	-622.1		
QCI-HEV	11.6	-0.8	11.1	0.5	10.7	-617.3		

5.2 Ir-catalyzed borylation

The workflow for automated generation of the reactant complex and the product geometry was performed using autodE v1.4.4 and xTB v6.4.0.

1. An initial reactant complex was generated by placing the chlorobenzene substrate near the active catalyst molecule and displacing them to ensure there is no atomic clash.

2. Rigid body rotations and translations were performed on the substrate and catalyst molecules to align them optimally according to the forming and breaking bonds based on the methodology implemented in autodE as described by Young et al.[11]

3. This reactant complex is then optimized using GFN2-xTB. During the optimization, the catalyst geometry was frozen, as optimization at the GFN2 level often breaks Ir-boron bonds. The final geometry is optimized at B97-D3/def2-SV(P)-CPCM(THF) level.

4. The product complex is obtained by enforcing distance constraints on the xTB level reactant complex—the Ir-C bond length is set to 2.17 Å, Ir-H to 1.72 Å and C-H to 1.6 Å for the relevant C-H bond being activated. This is optimized with GFN2-xTB, again keeping the catalyst frozen. The product complex is then minimized with B97-D3/def2-SV(P)-CPCM(THF) first with a constraint of 2.0 Å for the C-H bond, and then the constraint is removed and the geometry is minimized again.

Rate constant ratio

Under the assumption of kinetic control at the C−H activation step, the ratio of the *ortho*-, *meta*- and *para*- products will be equal to the ratio of the rate constants (k_r) for the respective reactions. The Eyring-Polanyi equation is used to link the Gibbs free energy of activation to the rate constant.

$$k_r = \frac{\kappa k_{\mathrm{B}} T}{h} e^{-\frac{\Delta G^{\ddagger}}{RT}} \tag{6}$$

where, κ is the transmission coefficient, k_{B} is the Boltzmann constant, T is the absolute temperature, h is the Planck constant, $\Delta G^{\ddagger}$ is the molar Gibbs free energy of activation and R is the universal gas constant.

Table S28: **Ir-catalyzed borylation.** Thermochemical parameters for RS, PS and TSs at geometry and single-point levels of theory, in units of $kcal\,mol^{-1}$ for separately calculated catalyst and substrate, RS complex (i.e. catalyst with substrate), PS and TS for activation of different C−H positions, given at two levels of theory. Imaginary frequencies (ω_i) in units of cm^{-1} are also quoted for the TSs.

		ΔE	ΔZPE	ΔH	$T\Delta S$	ΔG	$\Delta_{soln}G$	ω_i
B97-D3/def2-SV(P), CPCM(THF)								
	catalyst + substrate	0.0	0.0	0.0	0.0	0.0	0.0	–
ortho	RS complex	-22.4	1.1	-20.7	-17.0	-3.7	-5.6	
ortho	TS	18.0	-1.5	16.6	-19.4	35.9	34.0	-472.2
ortho	PS	7.6	0.4	8.0	-19.5	27.5	25.6	
meta	RS complex	-22.4	1.0	-20.7	17.0	-3.7	-5.6	
meta	TS	8.0	-1.6	6.6	-17.8	24.4	22.5	-720.6
meta	PS	-3.4	0.2	-3.1	-18.6	15.5	13.6	
para	RS complex	-22.0	1.0	-20.3	-16.8	-3.5	-5.4	
para	TS	12.7	-2.2	11.0	-16.8	27.8	25.9	-690.7
para	PS	-2.3	0.1	-1.9	-18.4	16.5	14.6	
B2K-PLYP/def2-TZVP//B97-D3/def2-SV(P), CPCM(THF)								
	catalyst + substrate	0.0	–	0.0	–	0.0	0.0	
	ortho-TS	23.3	–	21.8	–	41.2	39.3	
	meta-TS	7.7	–	6.4	–	24.2	22.2	
	para-TS	10.6	–	8.9	–	25.7	23.8	

We assume that κ is the same in all cases, then the ratio of rate constants is

$$k_o : k_m : k_p = \exp\left(-\frac{\Delta G_o^\ddagger}{RT}\right) : \exp\left(-\frac{\Delta G_m^\ddagger}{RT}\right) : \exp\left(-\frac{\Delta G_p^\ddagger}{RT}\right) \tag{7}$$

with the subscripts o, m and p indicating the *ortho*-, *meta*- and *para*-TSs respectively. Since the *meta*-TS is the lowest in energy, multiplying each term in the ratio by $\exp\left(+\frac{\Delta G_m^\ddagger}{RT}\right)$ gives:

$$k_o : k_m : k_p = \exp\left(-\frac{\Delta G_o^\ddagger - \Delta G_m^\ddagger}{RT}\right) : 1 : \exp\left(-\frac{\Delta G_p^\ddagger - \Delta G_m^\ddagger}{RT}\right) \tag{8}$$

Substituting the values from the table, at B2K-PLYP/def2-TZVP//B97-D3/def2-SV(P), CPCM(THF) level of theory and $T = 353.15\,K$, we obtain

$$k_o : k_m : k_p = 3 \times 10^{-11} : 1 : 0.12 \tag{9}$$

$$= 0\% : 89\% : 11\% \tag{10}$$

References

(1) Liu, Y.; Qi, H.; Lei, M. Improved Elastic Image Pair Method for Finding Transition States. *J. Chem. Theory Comput.* **2023**, *19*, 2410–2417.

(2) Dewar, M. J. S.; Healy, E. F.; Stewart, J. J. P. Location of Transition States in Reaction Mechanisms. *J. Chem. Soc., Faraday Trans. 2* **1984**, *80*, 227–233.

(3) Fletcher, R.; Powell, M. J. D. A Rapidly Convergent Descent Method for Minimization. *The Computer Journal* **1963**, *6*, 163–168.

(4) Gonzalez, C.; Schlegel, H. B. An Improved Algorithm for Reaction Path Following. *J. Chem. Phys.* **1989**, *90*, 2154–2161.

(5) Farkas, Ö.; Schlegel, H. B. Methods for Optimizing Large Molecules. II. Quadratic Search. *J. Chem. Phys.* **1999**, *111*, 10806–10814.

(6) Halgren, T. A.; Lipscomb, W. N. The Synchronous-Transit Method for Determining Reaction Pathways and Locating Molecular Transition States. *Chem. Phys. Lett.* **1977**, *49*, 225–232.

(7) Smidstrup, S.; Pedersen, A.; Stokbro, K.; Jónsson, H. Improved Initial Guess for Minimum Energy Path Calculations. *J. Chem. Phys.* **2014**, *140*, 214106.

(8) Bofill, J. M. Updated Hessian matrix and the restricted step method for locating transition structures. *J. Comput. Chem.* **1994**, *15*, 1–11.

(9) Iron, M. A.; Janes, T. Evaluating Transition Metal Barrier Heights with the Latest Density Functional Theory Exchange–Correlation Functionals: The MOBH35 Benchmark Database. *J. Phys. Chem. A* **2019**, *123*, 3761–3781.

(10) Semidalas, E.; Martin, J. M. The MOBH35 Metal–Organic Barrier Heights Reconsidered: Performance of Local-Orbital Coupled Cluster Approaches in Different Static Correlation Regimes. *J. Chem. Theory Comput.* **2022**, *18*, 883–898.

(11) Young, T. A.; Silcock, J. J.; Sterling, A. J.; Duarte, F. autodE: Automated Calculation of Reaction Energy Profiles— Application to Organic and Organometallic Reactions. *Angew. Chem. Int. Ed.* **2021**, *133*, 4312–4320.
